# The oxidation of hydrocarbons by diverse heterotrophic and mixotrophic bacteria that inhabit deep-sea hydrothermal ecosystems

**DOI:** 10.1038/s41396-020-0662-y

**Published:** 2020-04-30

**Authors:** Wanpeng Wang, Zhenyu Li, Lingyu Zeng, Chunming Dong, Zongze Shao

**Affiliations:** 1 Key Laboratory of Marine Genetic Resources, Third Institute of Oceanography, Ministry of Natural Resources, Xiamen, China; 2 State Key Laboratory Breeding Base of Marine Genetic Resources, Third Institute of Oceanography, Xiamen, China; 3 Fujian Key Laboratory of Marine Genetic Resources, Xiamen, China

**Keywords:** Environmental microbiology, Microbial ecology, Biogeochemistry

## Abstract

Hydrothermal activity can generate numerous and diverse hydrocarbon compounds. However, little is known about the influence of such hydrocarbons on deep-sea hydrothermal microbial ecology. We hypothesize that certain bacteria live on these hydrocarbons. Therefore, in this study, the distribution of hydrocarbons and their associated hydrocarbon-degrading bacteria were investigated at deep-sea hydrothermal vents at the Southern Mid-Atlantic Ridge, the Southwest Indian Ridge, and the East Pacific Rise. A variety of hydrocarbon-degrading consortia were obtained from hydrothermal samples collected at the aforementioned sites after low-temperature enrichment under high hydrostatic pressures, and the bacteria responsible for the degradation of hydrocarbons were investigated by DNA-based stable-isotope probing with uniformly ^13^C-labeled hydrocarbons. Unusually, we identified several previously recognized sulfur-oxidizing chemoautotrophs as hydrocarbon-degrading bacteria, e.g., the SAR324 group, the SUP05 clade, and *Sulfurimonas*, and for the first time confirmed their ability to degrade hydrocarbons. In addition, *Erythrobacter*, *Pusillimonas*, and SAR202 clade were shown to degrade polycyclic aromatic hydrocarbons for the first time. These results together with relatively high abundance in situ of most of the above-described bacteria highlight the potential influence of hydrocarbons in configuring the vent microbial community, and have made the importance of mixotrophs in hydrothermal vent ecosystems evident.

## Introduction

Hydrothermal activity can generate a variety of reduced compounds, including low molecular weight hydrocarbons, which can be produced abiotically through water–rock interactions under high temperature and pressure [1–4]. It was recently discovered that Fe^2+^ in hydrothermal systems is oxidized by water formed oxygen to give magnetite (Fe_3_O_4_), while the water is reduced to H_2_. Ultimately, the H_2_-dependent reduction of CO_2_ leads to the generation of hydrocarbons (C_2_–C_11_), methane, and aromatics using Fe_3_O_4_ as the catalyst [5, 6]. Long carbon chain alkanes and polycyclic aromatic hydrocarbons (PAHs) can also be generated in the deep subsurface via thermogenic processes [2, 3, 7, 8]. For instance, numerous hydrocarbons have been observed in the vent fluids and sulfide deposits of the Rainbow vent field at the north Mid-Atlantic Ridge (MAR), including C_9_–C_14_ *n*-alkanes, C_9_–C_13_ branched alkanes, C_9_–C_11_ cycloalkanes, C_7_–C_12_ nonaromatic hydrocarbons, naphthalene, methyl-naphthalene, and C_13_–C_16_ PAHs (fluorene, phenanthrene, and pyrene) [1]. Similarly, a high abundance of *n-*alkanes of C_15_–C_30_ chains, and three- or four-ringed PAHs were detected in the hydrothermal sediments from the Lost City vent field at the northern part of the MAR [9].

The deep biosphere may be partially energetically supported by hydrocarbons [10]; however, we know little about this unique ecosystem. Deep-sea hydrothermal vent areas may foster harvesting of deep matter and energy, by unique extremophiles and provide clues to understand the coupling of deep-sea life and abiotic and biotic processes under the seafloor. Recently, alkane oxidation genes that encode short-chain alkane monooxygenases, degradation pathways for corresponding alcohols, and short-chain fatty acids were found to be abundant in the hydrothermal plume metatranscriptome and metagenome, and these genes may be derived from the uncultivated bacterial group SAR324 [11–13]. In addition, a high diversity of alkane monooxygenases that were phylogenetically affiliated with enzymes involved in C_1_–C_4_ alkane oxidation was observed in the Guaymas Basin hydrothermal plume [14, 15]. Moreover, genes involved in anaerobic hydrocarbon degradation were also detected among several phyla in Guaymas Basin sediments, including Bacteroidetes, Chloroflexi, Deltaproteobacteria, and the candidate phylum Latescibacteria (WS3) [16]. Metagenomic and metatranscriptomic approaches revealed the presence of diverse methyl-coenzyme M reductase-based alkane-oxidizing archaea, including the multi-carbon alkane oxidizer *Ca*. Syntrophoarchaeum spp., anaerobic methane-oxidizing archaea (ANME-1 and ANME-2c), and sulfate-reducing bacteria (HotSeep-1 and Seep-SRB2) coexisting with sulfate-reducing bacteria and showed the potential for alkane oxidization in Guaymas Basin hydrothermal sediments [17]. However, these advances are mainly based on metadata, while few hydrocarbon-oxidizing microbes have been isolated from deep ecosystems.

In the past decade, we have explored the bacterial diversity involved in PAH degradation in deep-sea sediments of the MAR [18], the west Pacific [19, 20], and the Arctic [21], as well as in the deepwater columns of the southwest Indian Ridge [22].

However, few studies have been conducted within or in the vicinity of the hydrothermal vent field. In the past 10 years, we joined oceanic cruises and collected hydrothermal vent samples from the Southern Mid-Atlantic Ridge, the Southwest Indian Ridge, and the East Pacific Rise to examine the diversity of bacteria that may be driven by hydrocarbons *in situ*. Here we report the microbial diversity of aliphatic and aromatic hydrocarbons degrading bacteria in the vent plumes, chimney sulfides, and nearby sediments, and confirm their activity under in situ conditions. The results extend the body of knowledge of the potentially hydrocarbon-utilizing microbial community inhabiting the hydrothermal vent ecosystem, and promote understanding of their interactions with extreme environments.

## Materials and methods

### Deep-sea sampling, chemicals, and enrichment media

Samples and their descriptions are provided in Table S1 and Fig. S1 and the Supplementary Materials and Methods. Detailed descriptions of the chemicals and enrichment media are also described in the Supplementary Materials and Methods.

### Hydrocarbon analysis

To determine the hydrocarbon concentrations in hydrothermal plume samples, a method that combined stir bar sorptive extraction, thermal desorption–gas chromatography–mass spectrometry, and the Hydro-CARB® software package (IFP, Rueil-Malmaison, France) was used. The details of these procedures are described in the Supplementary Materials and Methods.

### Enrichment of hydrocarbon-degrading bacteria at high pressure

Deep-sea-mimicking cultivation was conducted under high pressures and low temperatures in the chamber of a HP vessel as described in the Supplementary Materials and Methods.

### Stable-isotope probing experiments

Stable-isotope probing (SIP) experiments for the above plume, sulfide deposit, and sediment enrichments were performed with ^13^C-labeled alkanes and PAHs, yielding a total of 12 samples. The details of these procedures are described in the Supplementary Materials and Methods.

### Isolation of heterotrophic hydrocarbon-degrading bacteria

Serial dilutions of enrichments were streaked onto M2 agar plates, then incubated at 15 °C until the formation of bacterial colonies was observed. Colonies exhibiting unique morphological features were selected and re-streaked onto M2 plates to obtain pure cultures that were then preserved at −20 °C for further analyses.

### Cultivation and isolation of chemoautotrophs from hydrocarbon-degrading consortia

Cultures and isolates were identified as previously described [23, 24], with slight modifications. The details of these procedures are described in the Supplementary Materials and Methods.

### Hydrocarbon degradation by consortia and isolates under high hydrostatic pressures and low temperature

The hydrocarbon degradation procedures are described in detail in the Supplementary Materials and Methods.

### Analysis of bacterial community structures

A detailed description of the analysis of bacterial community structures procedures is described in the Supplementary Materials and Methods.

### Nucleotide sequence availability

All of the Illumina sequence data from this study were submitted to the NCBI Sequence Read Archive under accession numbers SRR067098 and SRR10061063–10061075. Sanger-sequenced 16S rDNA sequences from the isolates were submitted to GenBank under accession numbers KT581452–KT581573, KT596765–KT596769, and MT084040–MT084046.

## Results

### Hydrocarbons in deep-sea hydrothermal field samples

**Fig. 1:**
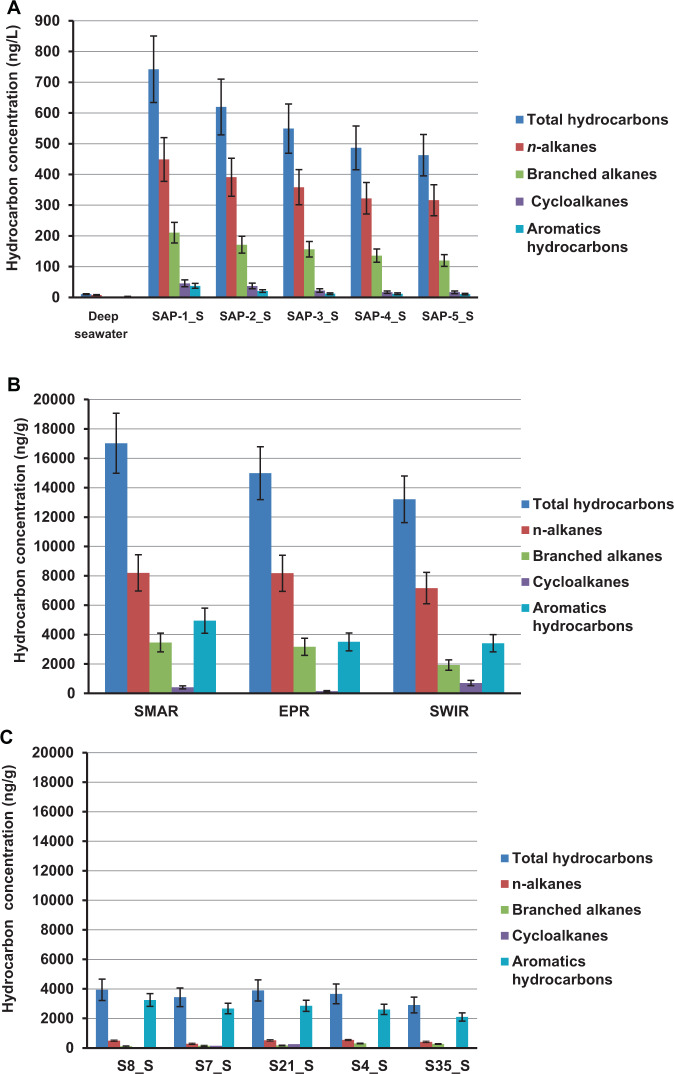
Hydrocarbon concentrations in deep-sea hydrothermal samples. Hydrothermal vent plume samples (**a**); hydrothermal chimney samples (**b**); hydrothermal sediment samples (**c**). Data are the means of three independent measurements.

The concentration and composition of hydrocarbons were measured in the samples from hydrothermal fields at the southern Mid-Atlantic Ridge (SMAR), southwest Indian Ridge (SWIR), and East Pacific Rise (EPR), and in vent plumes, sulfide chimney samples, and sediments (Table S2). In the five plume samples from SMAR, the total hydrocarbon concentrations (THC) ranged from 462.6 to 742.1 μg·L^−1^ (Fig. 1a). In contrast, THC in the nearby non-plume deep-sea water was only 10.3 μg·L^−1^ (Table S2). In the chimney sulfide samples, THCs were present at concentrations of 17.1, 15.3, and 13.2 μg·g^−1^ (dry weight) in the SMAR, EPR, and SWIR samples, respectively (Table S2), with PAHs, *n*-alkanes (C_14_–C_28_), and branched alkanes (C_16_–C_20_) as the major components (Fig. 1b). In the hydrothermal sediments, the THCs ranged from 2.9 to 3.9 μg·g^−1^ (dry weight), with high concentrations of polycyclic aromatic hydrocarbons containing 2–5 rings detected in all of the sediment samples; however, only a few types of alkanes were detected, and these were present at low concentrations (Fig. 1c and Table S2).

### Bacterial diversity in the hydrothermal plume *in situ*

**Fig. 2:**
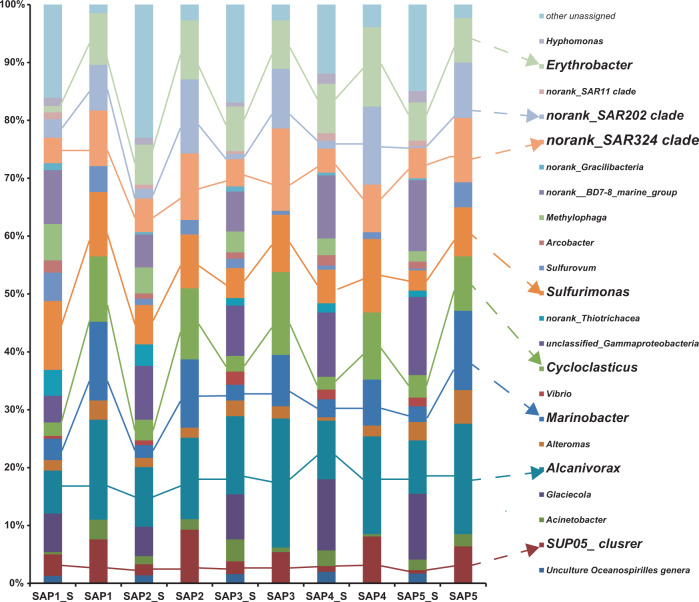
Taxonomic distribution among the five hydrothermal vent plume indigenous consortia (SAP1_S to SAP5_S) and hydrocarbon-enrichment SIP consortia (SAP1 to SAP5). Only genera that represent >3% of the communities in at least one sample are indicated. The “unassigned” categories represent all of the groups comprising <3% of the communities.

The bacterial community composition of the plume samples of the newly discovered hydrothermal field named Deyin-1 on the south MAR (15°S) is shown in Fig. 2. In the rising plume sample (SAP-1_S), the 16S rRNA gene sequences related to gamma-proteobacteria (31.5%) and epsilon-proteobacteria (19.2%) were highly abundant (Fig. 2). Among the detected gamma-proteobacteria, levels of the following genera were relatively high *Alcanivorax* (7.4% of the total), *Glaciecola* (6.7%), *Marinobacter* (3.7%), SUP05 clade sequences (3.7%), *Cycloclasticus* (2.3%), and *Alteromonas* (1.8%) (Fig. 2). Among the epsilon-proteobacteria sequences, the genera *Sulfurimonas* (11.9%), *Sulfurovum* (4.9%), and *Arcobacter* (2.1%) were present at relatively high concentrations (Fig. 2a). Additionally, the SAR324 clade (4.4%) of delta-proteobacteria and the SAR202 clade (3.2%) of Chloroflexi were detected in the rising plume sample (Fig. 2).

In the two neutrally buoyant plume samples (SAP-2_S to 5_S), which were collected far from the vent compared with the samples described above, the sequence reads mainly corresponded to gamma-proteobacteria (34.3–47.2%), epsilon-proteobacteria (5–8.8%), and alpha-proteobacteria (8.1–10.3%) (Fig. 2). The abundance of epsilon-proteobacteria decreased and alpha-proteobacteria appeared. The gamma-proteobacteria were mainly composed of *Alcanivorax* (9.2–10.3%), *Cycloclasticus* (2.2–3.9%), *Glaciecola* (5.1–11.4%), *Marinobacter* (2.2–3.1%), the SUP05 clade (0.6–2.2%), and *Alteromonas* (0.6–3.2%), among which the first two are well known for alkane and PAHs degradation. The epsilon-proteobacteria mainly were mainly composed of *Sulfurimonas* (3.5–6.8%), *Sulfurovum* (0.3–1.6%), and *Arcobacter* (0.9–1.8%). Notably, the genus *Erythrobacter* of alpha-proteobacteria also occurred as a dominant member, accounting for 6.6–8.5% of the total bacterial 16S rRNA gene sequences. In addition, clade SAR324 of delta-proteobacteria and clade SAR202 of Chloroflexi also occurred as the dominant bacteria *in situ*, comprising 4.1–5.8% and 0.9–1.7% of the total 16S rRNA sequence reads, respectively.

### Hydrocarbon biodegradation in the hydrothermal plume

To confirm the bacteria were utilizing hydrocarbons from the hydrothermal plumes, enrichment with a mixture of *n*-alkanes and PAHs as the carbon and energy sources was conducted while mimicking the deep-sea conditions of high static pressure and low temperature. Quantification showed that both alkanes and PAHs could be degraded significantly by the five plume-derived enrichment consortia (SAP-1 to SAP-5) under 20 MPa and at 10 °C. Specifically, nearly all *n*-alkanes were removed after 60 days, with the degradation percentages of 91.7–96.5% (Table S3), while 74.6–84.1% of the total PAHs were removed (Table S3).

Further, bacteria capable of hydrocarbon degradation were retrieved by SIP-sequencing. Four genera of bacteria were enriched with alkanes and the PAHs mixture as follows: *Alcanivorax* (14.1–22.3%), *Marinobacter* (7.9–13.1%), *Cycloclasticus* (9.4–14.3%), and *Erythrobacter* (7.7–13.7%) (Fig. 2). Unexpectedly, the previously recognized chemoautotrophic bacteria were also retained in all of the SIP communities, including the genus *Sulfurimonas* and the SUP05 and SAR324 clades.

### Bacterial diversity in hydrothermal chimneys

**Fig. 3:**
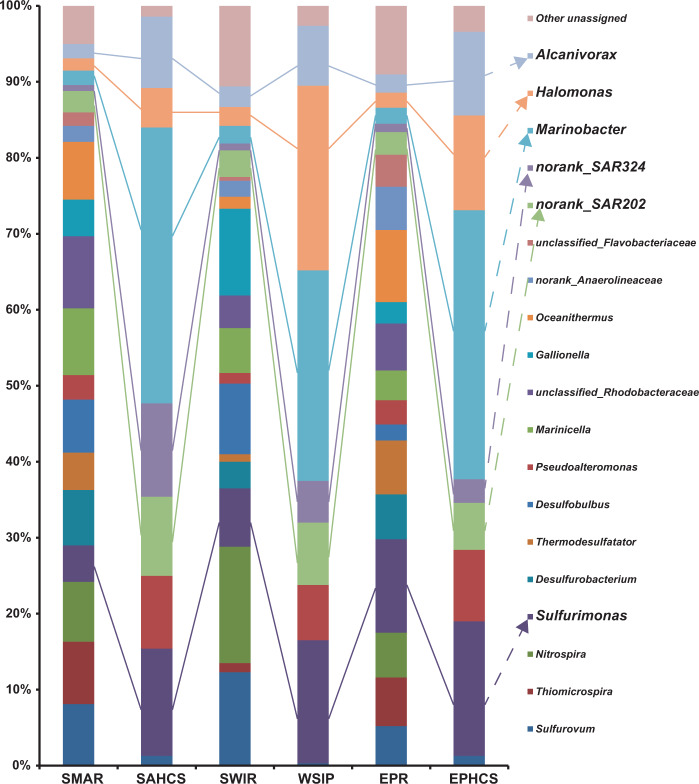
Taxonomic distribution among the three hydrothermal chimney indigenous consortia (SAMR, SWIR, and EPR) and hydrocarbon-enrichment SIP consortia (SAHCS, WSIP, and EPHCS). Only genera that represented >3% of the communities in at least one sample are indicated. The “unassigned” categories represented all of the groups comprising <3% of the communities.

The detailed bacterial diversity of black smoker chimney samples collected from three active hydrothermal fields in the SWIR, SMAR, and EPR are shown in Fig. 3. Despite the great geographic distance, the bacterial compositions at the three sites were similar, being mainly composed of *Sulfurovum*, *Sulfurimonas*, *Thiomicrospira, Nitrospira*, *Desulfurobacterium*, *Thermodesulfatator*, *Desulfobulbus*, *Pseudoalteromonas*, *Marinicella*, *Gallionella*, *Marinobacter*, *Halomonas*, and *Alcanivorax*, although they did vary to some extent. Additionally, the SAR202 clade was prevalent the indigenous consortia of all three sulfide chimneys.

### Hydrocarbon-degrading bacteria from the hydrothermal chimneys

To identify the hydrocarbon-degrading microbes inhabiting the hydrothermal chimneys, enrichment with a hydrocarbon mixture was conducted under hydrostatic pressures (20–35 MPa) and 10 °C according to the water depths and *in situ* temperatures, after which the hydrocarbon biodegradation capability of the hydrothermal chimney-derived consortia was tested. Within 20 days, 55–66% of the total *n*-alkanes and 20–35% of the total PAHs had been degraded. At day 40, 77–89% of the total *n*-alkanes and 44–52% of the total PAHs had been degraded, while at day 60, 92–97% of the total *n*-alkanes and 76–78% of the total PAHs had been degraded (Table S4). To define what types of bacteria in hydrothermal chimneys were hydrocarbon degraders, SIP-Seq data analysis was conducted after enrichment with ^13^C-labeled hydrocarbons for 60 days under 30 MPa at 10 °C. The results revealed that the SIP community mainly contained the following genera: *Marinobacter* (27.7–36.3%), *Sulfurimonas* (14.1–17.7%), *Halomonas* (5.2–24.3%), and *Pseudoalteromonas* (7.3–9.6%), as well as bacteria of clade SAR202 (6.2–10.4%) and clade SAR324 (3.1–12.3%). Among these, *Marinobacter* had the highest abundance, followed by *Sulfurimonas* and *Halomonas*. Congruent with the *in situ* diversity, despite the large geographic distance, quite similar degrading bacterial communities were obtained from the three global ocean hydrothermal chimney samples. Detailed compositions of the above hydrocarbon-degrading consortia are shown in Fig. 3.

### Bacterial diversity in hydrothermal sediments

**Fig. 4:**
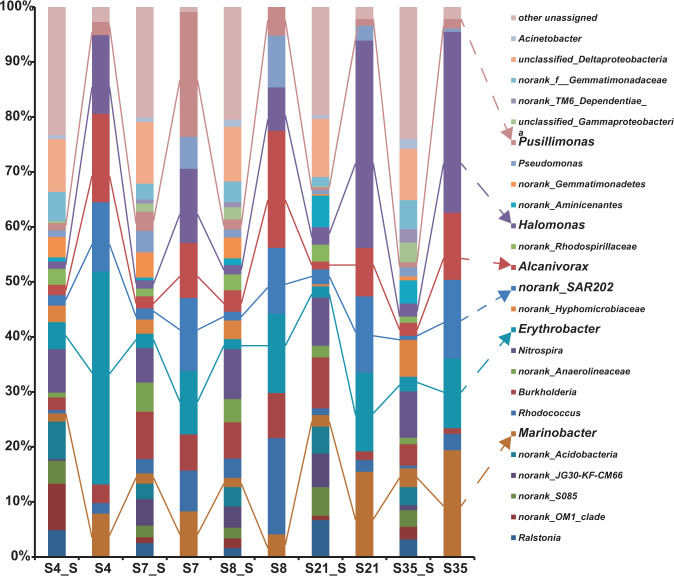
Taxonomic distribution among the five hydrothermal sediment indigenous consortia (S4_S, S7_S, S8_S, S21_S, and S35_S) and PAH-enrichment SIP consortia (S4, S7, S8, S21, and S35). Only genera that represent >3% of the communities in at least one sample are indicated. The “unassigned” categories represent all of the groups comprising <3% of the communities.

Bacterial compositions of the five hydrothermal sediment samples collected from the SWIR, SMAR, and EPR sites are shown in Fig. 4. The following bacteria were characterized as the dominant members in the five sediments based on their 16S rRNA gene abundance: *Nitrospira* (6.3–9.1%), *Erythrobacter* (1.8–4.9%), *Burkholderia* (2.3–9.3%), *Alcanivorax* (1.4–3.9%), *Marinobacter* (1.5–3.4%), *Rhodococcus* (0.5–3.5%), *Halomonas* (1.3–3.2%), *Pseudomonas* (0.7–3.9%), *Pusillimonas* (0.5–3.5%), *Acinetobacter* (0.6–1.8%), and the SAR202 clade (0.7–2.7%) (Fig. 4). In addition, the bacterial no-rank OM1 clade, S085 clade, JG30-KF-CM66 clade, *Acidobacteria*, *Anaerolineaceae*, *Hyphomicrobiaceae*, *Rhodospirillaceae*, *Aminicenantes*, *Gemmatimonadetes*, *Gemmatimonadaceae*, and unclassified gamma-proteobacteria frequently occurred in different sediment samples (Fig. 4).

### Bacterial PAHs biodegradation in hydrothermal sediments

All five hydrothermal sediment consortia enriched with PAHs showed obvious degradation activity under 35 MPa and at 10 °C (Table S5). In addition, all five types of added PAHs of 2–5 rings were significantly degraded. At day 90, naphthalene was totally degraded, while 82–89% of fluoranthene, 87–91% of phenanthrene, 80–94% of pyrene, and 38–50% of benzo[a]pyrene was degraded (Table S5).

To identify the key PAH degraders among the associated bacteria, the above SIP samples were enriched with the PAH mixture for 90 days under 35 MPa and 10 °C, then further processed by SIP-Seq. The results revealed nine bacterial genera were retained as ^13^C-labeled bacterial members in all degradation communities of the five sediment samples; namely, *Erythrobacter*, *Halomonas*, the SAR202 clade, *Alcanivorax*, *Marinobacter*, *Burkholderia*, *Pseudomonas*, *Pusillimonas*, and *Rhodococcus* (Fig. 4). However, the abundance of these organisms varied among samples, with the first four being the most abundant members. Nevertheless, these labeled bacteria were all suggested to be key PAH degraders, such as *Pusillimonas* and *Rhodococcus* in the two SWIR consortia S7 and S8 and *Marinobacter* in consortium SMAR (S21) and EPR (S35).

### Isolation of chemoautotrophs from enrichment cultures

**Fig. 5:**
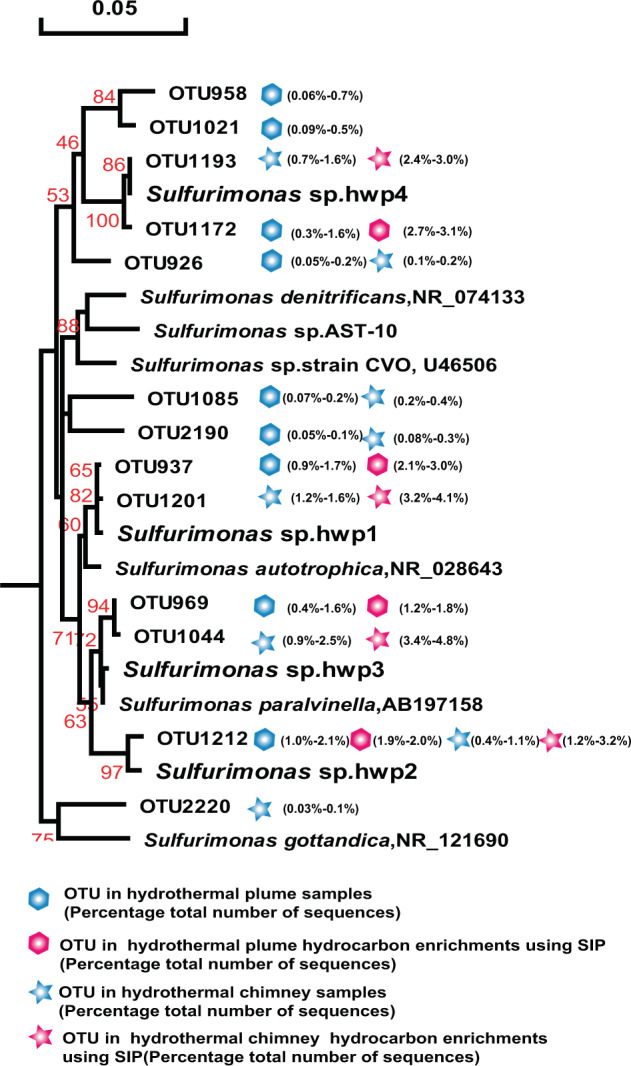
*Sulfurimonas* distribution among consortia and phylogenetic analysis of 16S rRNA gene phylotypes. Phylogenetic tree showing the diversity of 16S rRNA gene sequences from OTUs and isolates of *Sulfurimonas* identified in this study. The tree was constructed using neighbor-joining methods and the Kimura 2-parameter model, as implemented in the MEGA 5.0 software package. The tree is based on partial 16S rRNA gene sequences from this study and their closest type strains. Only bootstrap values ≥50% (based on 1000 bootstrap replicates) are shown at the nodes. The scale bar represents 0.05 nucleotide changes per site. *Sulfurimonas* OTU distribution among the different hydrocarbon-enrichment consortia and indigenous consortia. Only OTUs representing >1% of the communities in at least one sample are included in the visualization. OTU representative sequences are shown in the Supplementary Information.

**Fig. 6:**
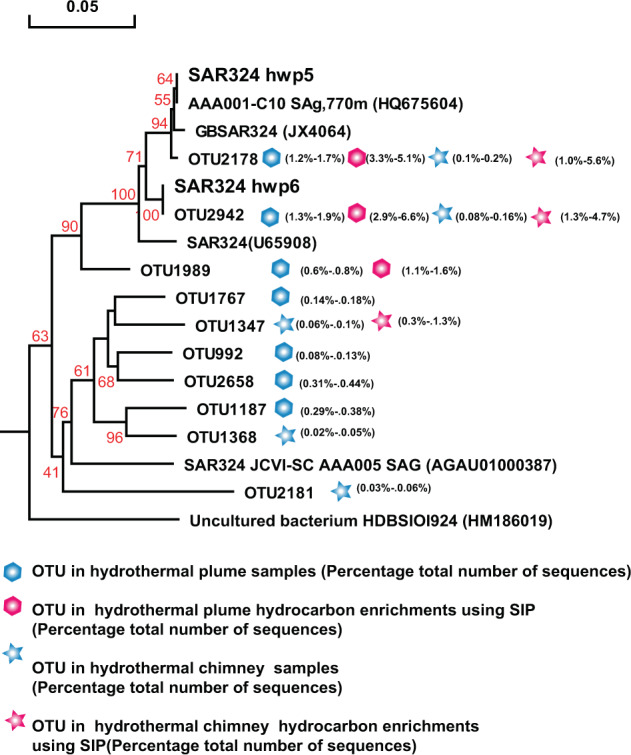
SAR324 distribution among consortia and phylogenetic analysis of 16S rRNA gene phylotypes. Phylogenetic tree showing the diversity of 16S rRNA gene sequences from OTUs and isolates of SAR324 identified in this study. The tree was constructed using neighbor-joining methods and the Kimura 2-parameter model, as implemented in the MEGA 5.0 software package. The tree is based on partial 16S rRNA gene sequences from this study and their closest type strains. Only bootstrap values ≥50% (based on 1000 bootstrap replicates) are shown at the nodes. The scale bar represents 0.05 nucleotide changes per site. SAR324 OTU distribution among the different hydrocarbon-enrichment consortia and indigenous consortia. Only OTUs representing >1% of the communities in at least one sample are included in the visualization. OTU representative sequences are shown in the Supplementary Information.

**Fig. 7:**
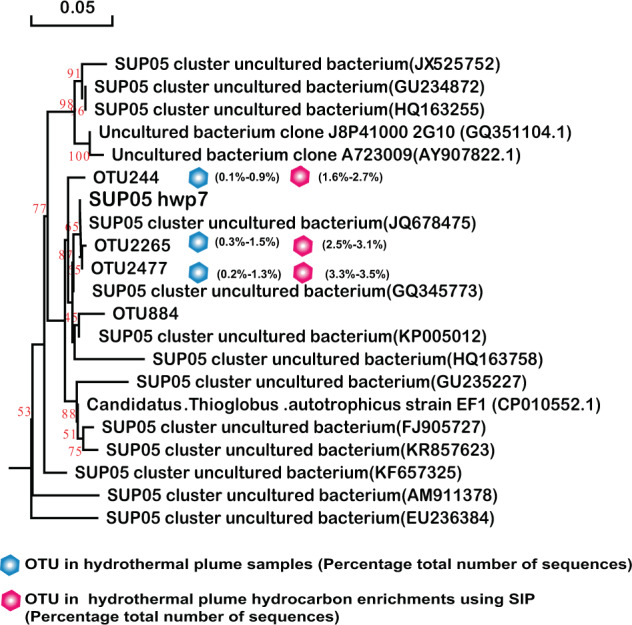
SUP05 distribution among consortia and phylogenetic analysis of 16S rRNA gene phylotypes. Phylogenetic tree showing the diversity of 16S rRNA gene sequences from OTUs and isolates of SUP05 identified in this study. The tree was constructed using neighbor-joining methods and the Kimura 2-parameter model, as implemented in the MEGA 5.0 software package. The tree is based on partial 16S rRNA gene sequences from this study and their closest type strains. Only bootstrap values ≥50% (based on 1000 bootstrap replicates) are shown at the nodes. The scale bar represents 0.05 nucleotide changes per site. SUP05 OTU distribution among the different hydrocarbon-enrichment consortia and indigenous consortia. Only OTUs representing >1% of the communities in at least one sample are included in the visualization. OTU representative sequences are shown in the Supplementary Information.

The once recognized chemoautotrophic bacteria, including the SAR324 clade, SUP05 clade, and *Sulfurimonas*, occurred as dominant members of the above hydrocarbon-degrading consortia of vent plumes and sulfides, and were confirmed by SIP-Seq. To better characterize their roles, further enrichment and pure culture isolation and further testing were conducted to determine their potential for hydrocarbon degradation. Thirty-seven out of 1536 culture wells from the above hydrothermal plume and chimney hydrocarbon enrichments were positive for growth, with sulfur oxidation occurring after 21 days. Moreover, four of the cultures had identical 16S rRNA gene sequences and were identified as members of *Sulfurimonas*, named strain *Sulfurimonas* sp. hwp1, hwp2, hwp3, and hwp4 (Fig. 5). Two cultures were identified as a delta-proteobacteria related to the SAR324 group, and named SAR324 strain hwp5 and strain hwp6 (Fig. 6). In addition, one culture was identified as a member of the SUP05 clade, named SUP05 strain hwp7 (Fig. 7).

### Hydrocarbon degradation by chemolithoautotrophs

The hydrocarbon degradation potential of pure cultures of *Sulfurimonas* spp., and the SAR324 and SUP05 clades were evaluated further under high HPs and low temperatures using hydrocarbon as the sole carbon and energy sources (adequate O_2_, remove CO_2_ and thiosulfate). All four isolates of *Sulfurimonas* and two isolates of SAR324 that were tested exhibited vigorous growth in the short chain length alkane assays using ^13^C-labeled *n-*hexane and *n*-octane under high HPs and low temperatures (Table 1 and Fig. S2). In addition, they also exhibited growth in the medium chain length alkanes of ^13^C-labeled *n-*decane and *n*-dodecane under high pressure and low temperature, but this was comparatively weak (Table 1 and Fig. S2). Only the isolates of SAR324 (strain hwp5 and hwp6) exhibited weak growth in the ^13^C-labeled *n-*hexadecane (Table 1 and Fig. S2). All four *Sulfurimonas* isolates exhibited weak growth under high HPs and low temperatures in the PAH assays using ^13^C-labeled naphthalene and phenanthrene, respectively (Table 1 and Fig. S2). None of the isolates grew with *n*-tetracosane, pyrene, or benzo(a)pyrene (Table 1). None of all tested hydrocarbons could be utilized by isolate hwp7 of SUP05 (Table 1).

**Table 1:** Mixotrophic bacteria growth capability with hydrocarbon in NM medium at high pressure and low temperature conditions.

Isolates	*n-*Hexane	*n-*Octane	*n-*Decane	*n-*Dodecane	*n-*Hexadecane	*n*-Tetracosane	Naphthalene	Phenanthrene	Pyrene	Benzo(a)pyrene
Hwp1 (*Sulfurimonas*)	**+++**	**+++**	**++**	**+**	**−**	**−**	**++**	**+**	**−**	**−**
Hwp2 (*Sulfurimonas*)	**+++**	**+++**	**++**	**+**	**−**	**−**	**++**	**+**	**−**	**−**
Hwp3 (*Sulfurimonas*)	**++**	**++**	**+**	**+**	**−**	**−**	**++**	**+**	**−**	**−**
Hwp4 (*Sulfurimonas*)	**++**	**++**	**+**	**+**	**−**	**−**	**++**	**+**	**−**	**−**
Hwp5 (SAR324)	**+++**	**+++**	**++**	**+**	**+**	**−**	**−**	**−**	**−**	**−**
Hwp6 (SAR324)	**+++**	**+++**	**++**	**+**	**+**	**−**	**−**	**−**	**−**	**−**
Hwp7(sup05)	**−**	**−**	**−**	**−**	**−**	**−**	**−**	**−**	**−**	**−**

(+++), (++), (+), and (+/−) indicating the growth capability from strong to weak with hydrocarbon as sole carbon and energy source, measured by optical density at 600 nm, (+++) growth (OD 600 > 0.6) after a 60-day incubation at 10 °C under 30 MPa; (++) growth (0.6 > OD 600 > 0.2) after a 60-day incubation at 10 °C under 30 MPa; (+) growth (OD 600 < 0.2) after a 60-day incubation at 10 °C under 30 MPa, (−) no growth.

All four *Sulfurimonas* isolates exhibited hydrocarbon degradation and degrade *n*-alkanes of C_6_–C_16_ as well as naphthalene and phenanthrene (Table 1). These phylotypes were closely related to the predominant members (>99.7% homology with 450 bps 16S rRNA) in plumes and sulfides *in situ* as well as those in the hydrocarbon-degrading consortia. Based on the OTU sequences and our isolates, a phylogenetic tree was constructed with references of the type strains (Fig. 5). The results showed that the four isolates were affiliated with four species, among which strain hwp4 isolated from the chimney of the new hydrothermal field Deyin in the southern MAR represented a novel species forming a separate cluster with the homologue OTU1193 from the same chimney sample and OTU1172 from the plume also located at Deyin (pink in Fig. 5), in addition to other members identified *in situ* (labeled blue). Moreover, strain hwp2 represented a novel species with 96.6% homology to the full-length 16S of *Sulfurimonas paralvinella*, whereas the strain hwp3 and hwp4 belonged to *Sulfurimonas autotrophica* and *S. paralvinella*, respectively. Despite the significant divergence in phylogeny, all four isolates possessed the same characteristics with respect to hydrocarbon utilization.

Similarly, SAR324 isolates hwp5 and hwp6 also exhibited positive alkane degradation (Table 1). Both were isolated from the Deyin field plume and shared 97.85% homology of the 16S rRNA gene, representing two potential novel species. A phylogenetic tree was constructed based on the 16S rRNA gene sequences of this study and the other four sequences retrieved from GenBank (Fig. 6). The phylogenetic result showed that this group in our study samples was quite diverse. The two isolates correspond to the predominant members represented by OTU2178 in the MAR plume and OTU2943 in the MAR sulfides and hydrocarbon-degrading consortia, respectively (Fig. 6). Among the tested *n*-alkanes from C_6_ to C_32_, both isolates could grow with *n*-hexane, octane, decane, dodecane, and hexadecane (Table 1), while they failed to grow with all tested odd alkanes of C_11_, C_15_, and C_17_, or with long-chain *n*-alkanes above C_20_ (data not shown). These isolates could not grow with any tested PAHs either (Table 1). Interestingly, SUP05 isolate hwp7 was closely related to the predominant members in plumes *in situ*, as well as the hydrocarbon-enrichment consortia, represented by OTU2265 and OTU2477 (Fig. 7); however, this organism was negative for hydrocarbon degradation (Table 1). Our isolate hwp7 together with other OTUs formed an independent cluster in the tree, neighboring with the cluster represented by the previously reported isolate *Candidatus* Thioglobus autotrophicus strain EF1 (Fig. 7). The two isolates showed 97.5% identity in the full-length 16S rRNA gene sequence, indicating they represent two potential novel species of the genus *Thioglobus*.

### Hydrocarbon degradation by heterotrophic isolates under high pressures

A total of 126 heterotrophic strains were isolated from the above hydrocarbon-enrichment consortia, and most could grow with hexadecane or PAH as the sole carbon and energy source. These organisms were affiliated with 16 genera, including *Alcanivorax*, *Acinetobacter*, *Alteromonas*, *Bacillus*, *Citreicella*, *Dietzia*, *Erythrobacter*, *Halomonas*, *Idiomarina*, *Marinobacter*, *Microbacterium*, *Novosphingobium*, *Sphingobium*, *Pseudomonas*, *Pusillimonas*, and *Spongibacter* (Table S6).

Thirteen of the isolates could grow with ^13^C-labeled *n-*hexadecane as the sole energy source under 35 MPa at 10 °C as determined by cell density OD_600_ (Fig. S3B), and the degradation was confirmed by quantification using the ^13^C isotope (Fig. S3A). Based on their 16S rRNA sequences, these isolates were identified as *Alcanivorax dieselolei* strain S19-9, *Alcanivorax* sp. strain YLF38, *Alcanivorax venustensis* strain RY-9, *Bacillus safensis* strain S21-L1, *Halomonas titanicae* strain RY-7, *Marinobacter bryozoorum* strain TG8-3, *Marinobacter hydrocarbonoclasticus* strain S19-1, *Marinobacter segnicrescens* strain S19-13, *Marinobacter vinifirmus* strain S19-10, *Oceanicola marinus* strain 22F16, *Oceanicola nanhaiensis* strain 1F26, *Rhodococcus yunnanensis* strain YLF8, and *Pusillimonas* sp. strain S7-N8 (Fig. S3A, B).

In the PAH assays, eight isolates exhibited noticeable growth under high HP and low temperature within 60 days based on ^13^C_6_-labeled phenanthrene analysis (Fig. S3C), while significant degradation occurred within 120 days under the same conditions (Fig. S3D). These isolates were identified and named as *Erythrobacter* sp. strain S35-N8, *Erythrobacter* sp. strain S21-N3, *Pusillimonas* sp. strain S7-N8, *E. flavus* Y strain LF25, *Marinobacter algicola* strain YLF36, *M. hydrocarbonoclasticus* strain S19-1, *B. safensis* strain S21-L1, and *B. safensis* strain S8-L9.

Interestingly, *Erythrobacter* sp. S21-N3 and *Pusillimonas* sp. S7-N8 exhibited vigorous growth using various PAHs including naphthalene–^13^C_6_, phenanthrene–^13^C_6_, pyrene–^13^C_6_, fluorene–^13^C_6_, and benzo[α]pyrene–^13^C_6_ (Figs. S3E, F, and S4).

## Discussion

Previous studies of microbial vent communities have mainly focused on chemolithoautotrophic organisms. These studies have shown that chemoautotrophic bacteria such as *Sulfurimonas* and clades of SAR324 and SUP05 were ubiquitous in the global deep-sea hydrothermal ecosystem and commonly recognized as the sulfur-oxidizing chemolithoautotrophs responsible for to the primary production of vent ecosystems. Although various hydrocarbons can be generated from the deep subsurface processes, little is known about their contribution to the determination of vent bacterial diversity. During our investigation, it is quite unexpected to find that fundamental chemoautotrophs such as *Sulfurimonas* and the SAR324 clade were capable of degrading hydrocarbons and active under simulated extreme conditions.

Interestingly, although the bacteria of the SUP05 clade were dominant members of all of the plume-derived hydrocarbon-degrading consortia, the purified isolate (strain hwp7) failed to degrade any tested hydrocarbons. However, genome analysis showed that the strain hwp7 genome harbored multiple genes encoding enzymes involved in aromatic or alkane carbon catabolism, including proteins with predicted functions in aromatic ring hydroxylation, alicyclic rings oxidation, catechol degradation, phenylpropionate degradation, fatty aldehydes oxidation, and fatty carboxylic acids oxidation (data not shown). Because it can grow without nutrient supplements such as vitamins and amino acids in medium of thiosulfate oxidization, we speculate that the SUP05 strain hwp7 may utilize the intermediate metabolites of hydrocarbons of other bacteria instead of via syntrophism.

The SAR324 group of delta-proteobacteria is ubiquitous in global oceans [14], and is one of the most abundant microbial groups in deep-sea hydrothermal plumes [15, 25]. To date, none of the bacteria comprising this group have been isolated, although it is well known as a sulfur-oxidizing bacterium [14]. Recently, metagenomic and metatranscriptomic analyses showed that the particulate hydrocarbon monooxygenase (pHMO), which is believed to be responsible for oxidizing short-chain alkanes of C_2_–C_4_, is present and expressed in the Guaymas Basin hydrothermal plume [11]. In the present, SAR324 not only occurred as one of the most abundant bacteria *in situ*, but also constituted the majority of the hydrocarbon SIP consortia from hydrothermal plumes and chimneys. Notably, two SAR324 isolates numbered hwp5 and hwp6 were isolated and 16S rRNA genes showed high similarity (98.8 and 98.3%) to SAR324 from the Guaymas Basin hydrothermal plume. Moreover, they both exhibited degradation activity on *n*-alkanes with chain lengths of C_6_–C_16_, but failed to grow with long chain alkanes of C_20_–C_32_. This degradation capability may be attributed to the genome encoded cytochrome P450, which catalyzes the hydroxylation of medium chain alkanes [26, 27]. We suspect that they can also oxidize short chain alkanes (C_2_–C_4_) because the corresponding gene encoding pHMO was found in their genomes (data not shown). To the best of our knowledge, this is the first report of a SAR324 group delta-proteobacteria being capable of alkane degradation, which highlights their role in alkane oxidation in natural environments. Moreover, the success of a pure culture will help us gain insight into the metabolic mechanisms and environmental interactions of these organisms in global oceans.

Bacteria of the genus *Sulfurimonas* are commonly isolated from sulfidic habitats, and numerous 16S rRNA sequences related to *Sulfurimonas* have been identified in hydrothermal deep-sea vents, marine sediments, pelagic water columns, and terrestrial habitats [28]. Although *Sulfurimonas* species play important roles in chemoautotrophic processes in some habitats [28, 29], some species can grow with organic substrates, such as *Sulfurimonas* sp. CVO, and *Sulfurimonas gotlandica* can use acetate in addition to carbon dioxide and bicarbonate as a carbon source [30, 31]. *Sulfurimonas denitrificans* can grow with a formate, fumarate, yeast extract, and alcohol mix as electron donors, while *S. gotlandica* can grow with formate, acetate, yeast extract, pyruvate, and amino acid mix as electron donors [31]. In this study, four isolates of *Sulfurimonas* could all grow well with C_6_–C_12_ *n*-alkanes as an electron donor and carbon source, and they were capable of degrading naphthalene. To the best of our knowledge, this is the first report of this genus being capable of utilizing hydrocarbons. In addition, these isolates can also oxidize reduced sulfur compounds. The versatility in mixotrophic metabolisms may explain the ubiquity of *Sulfurimonas* in the global hydrothermal vent biosphere. Mixotrophs in vent surroundings are probably common; therefore, investigations of other microbial taxa are needed.

There is very little information is available about the interactions of heterotrophic bacteria with hydrocarbons in deep-sea hydrothermal environments. Prior to this study, two mesophilic Proteobacteria, *Salinisphaera hydrothermalis*, and *Parvibaculum hydrocarboniclasticum* were isolated from deep-sea hydrothermal vents on the EPR and were capable of growth on *n-*alkanes as their sole carbon source [32, 33]. In addition, isolates of *Alcanivorax* and *Marinobacter* were recovered from hydrothermal environment samples of the 9°N EPR and the 37°N MAR [34]. However, their degradation capacity under in situ conditions remains untested. This study confirmed diverse heterotrophic bacteria inhabiting global deep-sea hydrothermal environments, possibly using hydrocarbon degradation as an energy source. By mimicking in situ conditions, we confirmed their capacity of active degradation of various hydrocarbons at HPs (2000–3500 m water depth) and low temperature. *Marinobacter* spp., *Halomonas* spp., and the SAR202 clade predominated bacterial communities in the hydrocarbon-degrading SIP consortia from the SMAR, SWIR, and EPR sulfide chimneys, respectively. *Alcanivorax* spp., *Marinobacter* spp., *Cycloclasticus* spp., and the SAR202 clade dominated the hydrocarbon-degrading SIP community of hydrothermal vent plumes. *Erythrobacter* spp., *Alcanivorax* spp., *Halomonas* spp., and the SAR202 clade were the most predominant members of the PAH-degrading consortia from all hydrothermal sediments, while *Pusillimonas* was the predominant genus in the SIP consortium from SWIR sediments at 49°E (S7). Notably, the above hydrocarbon-degrading bacteria also occurred the dominant members of the indigenous bacterial community from hydrothermal samples, suggesting they interacted with vent effluents and surrounding environments by utilizing hydrocarbons generated from deep subspaces.

SAR202, which is a cluster of uncultured bacteria affiliated with the phylum *Chloroflexi*, are medium-sized, free-living heterotrophic organisms [35–38]. Even in sediments at the bottom of the Challenger Deep of the Mariana Trench, SAR202 is the dominant population of bacterial communities [39]. Recently, Landry and colleagues analyzed five single-amplified genomes of the SAR202 clade and found that they encoded several families of oxidative enzymes such as ring-hydroxylating and ring-cleavage dioxygenases, cytochrome P450 and monooxygenase, which were believed to participate in hydrocarbon degradation [40]. In the present study, the SAR202 clade comprised 1.0–3.5% of the total bacterial 16S rRNA gene abundance in all tested samples. Further, bacteria of the SAR202 clade were the dominant population (about 6.0–14.5%) in all of the aforementioned hydrocarbon-degrading SIP consortia. Remarkably, the SAR202 clade became the dominant member in all five sediment-derived PAHs enrichments from SMAR, SWIR, and EPR (Fig. S5). Although the hydrocarbon metabolism of the SAR202 clade is still unknown, our results indicated that the SAR202 clade can degrade hydrocarbons, especially PAHs, and they might play a role in the degradation of recalcitrant organic matter in deep-sea hydrothermal fields. However, the bacteria of the SAR202 group need further investigation to confirm their ability for hydrocarbon degradation with pure cultures, which have yet to be developed.

Among these heterotrophic bacteria, *Pusillimonas* and *Erythrobacter* are less often reported in hydrocarbon degradation. Indeed, this is the first report of *Pusillimonas* being capable of PAH degradation. The isolate *Pusillimonas* sp. S7-N8 from our sediment enrichments showed only 96.7% similarity of the 16S rRNA gene with that of *Pusillimonas noertemannii* BN9^(T)^ (Table S5). This organism exhibited vigorous growth on alkanes and PAHs under high HPs and low temperature (Fig. S4). This is the first member of the genus *Pusillimonas* as a PAH-degrader. In the present study, *Erythrobacter* bacteria, which typically inhabit marine surface environments, were found to be are one of the predominant bacteria in vent plumes accounting for 6.6–8.5% of the total 16S rRNA gene sequences. Intriguingly, the isolate of *Erythrobacter* showed a wide PAHs degradation range covering 2–5 fused rings, including naphthalene, phenanthrene, pyrene, fluorene, and benzo[α]pyrene. Moreover, the novel species from our enrichments, *Erythrobacter atlantica* sp. S21-N3 [41], could grow well on PAHs at 30 MPa (5 °C).

In summary, various hydrocarbons were found in global deep-sea hydrothermal environments, and they had a unique composition pattern in vent plumes compared to chimney sulfides and sediments. Correspondingly, various hydrocarbon-degrading bacteria were found in these hydrothermal samples. Interestingly, the chemoautotrophic bacteria clade SAR324 and *Sulfurimonas* were capable of degrading either alkanes and/or PAHs. In addition, heterotrophic bacteria belonging to the genus *Alcanivorax*, *Cycloclasticus*, *Marinobacter*, *Halomonas*, *Pusillimonas*, and *Erythrobacter* were found *in situ* as dominant members capable of hydrocarbon oxidation. The results of the present study demonstrate that the deep-sea hydrothermal vent ecosystem fosters unique mixotrophic bacteria, in addition to obligate heterotrophs and chemolithotrophs.

## Supplementary information

Supplementary Materials and Methods

Supplementary Information
