# Supplementary material for: The oxidation of hydrocarbons by diverse heterotrophic and mixotrophic bacteria that inhabit deep-sea hydrothermal ecosystems
Source: ISME J. 2020 Apr 30;14(8):1994–2006. doi: 10.1038/s41396-020-0662-y (PMC7368058; doi:10.1038/s41396-020-0662-y)
Supplement: Supplementary file 1 — Supplementary Materials and Methods [file 41396_2020_662_MOESM1_ESM.docx]

**Supplementary Materials and Methods**

**Deep-sea sampling**

Samples and their descriptions are shown in Table S1. The SMAR hydrothermal plume was sampled using a conductivity, temperature, and depth (CTD) water sampler (SBE-911 plus, Sea-bird Scientific, Bellevue, WA USA) mounted onto a remotely operated vehicle (ROV), Hailong II (Institute of Oceanography, Shanghai Jiaotong University), during voyage DY115-22 (‘Dayang Yihao’ research vessel, 2011). An in-line temperature probe and inlet of an *in situ* mass spectrometry (ISMS) spectrometer [1] were attached to the sampling nozzle to measure temperature and gas concentrations directly at the sample sites. Sample sites were selected based on visible venting, increased temperatures, and elevated gas concentrations, as indicated by ISMS. The hydrothermal rising plume sample (SAP-1_S) was collected from diffuse fluids directly above (1 m, vertical direction) the vent orifice (14.4°W, 13.2°S). The neutrally buoyant plume samples (SAP-2_S to 5_S) were collected above the orifice (approximately 15 m, vertical direction) and in the vicinity of the vent (1–20 m, horizontal direction). Plume water samples were kept at 4°C and 30 MPa (*in situ* HP) using high HP reactors (HHPRs) until reaching the lab (30–40 days). Total DNA from plume water samples was extracted onboard.

Hydrothermal chimneys were sampled from SWIR, SMAR, and EPR using a remote-control robotic arm mounted to the ROV Hailong II and sealable bioboxes during the DY115-21, 22, and 26 cruises, respectively, between 2010 and 2012. The bioboxes prevent mixing with seawater as the submersible moves through the water column. Sulfide chimney samples were collected at three sites (SWIR, SMAR, and EPR, see Table S1), and samples of 300–600 g were collected. Samples were processed upon arrival on deck using sterile chisels and aluminum rock boxes that were specifically designed for sampling seafloor rocks. Subsamples were then placed at 4°C and 20 MPa (*in situ* HP) using HHPRs until further processing at the laboratory. The chimney samples were collected from the exterior chimney surfaces based on their spatial positions. Thus, the hydrothermal chimney deposit samples represent low temperature samples (Table S1), despite the chimneys being hydrothermally active. Hydrothermal sediment push cores were collected from the SWIR, SMAR, and EPR vents using 45 cm long polycarbonate cores with 6.25 cm interior diameters that were mounted on the ROV Hailong II. Push cores were collected at five sites (Table S1), yielding approximately 500–1000 g of sediments that were preserved in sterilized sampling bags at 4°C and 30–35 MPa (*in situ* HP) using HHPRs. The sediment sampling sites were located at the vent vicinity (10–100 m, horizontal direction). Total DNA from hydrothermal chimneys and sediments was also extracted onboard. All of the samples were immediately divided on board the ship for chemical analyses.

**Chemicals and enrichment media**

Non-labeled alkane and PAH substrates, and ^13^C-labeled hydrocarbons including octane-^13^C_2_, decane-^13^C_2_, dodecane-^13^C_4_, hexadecane-^13^C_4_, eicosane-^13^C_6_, tetracosane-^13^C_6_, phenanthrene-^13^C_6_, naphthalene-^13^C_6_, pyrene-^13^C_6_, fluoranthene -^13^C_6_, and benzo[α]pyrene-^13^C_8_, were purchased from Sigma-Aldrich Chemie (Steinheim, Germany). A 16-PAH standard solution was purchased from Fluka (> 99.3% purity), and the 20-alkane standard solution (99.8% purity) was purchased from Sinopharm Chemical Reagent (Shanghai, China).

NH medium was used to enrich alkane and PAH-degrading microorganisms, as well as to assess hydrocarbon biodegradation capacity [2]. In addition, M2 medium was used to further cultivate microorganisms from enriched cultures [3]..

**Hydrocarbon Analysis**

To determine hydrocarbon concentrations in the hydrothermal plume samples, a method that combined a Stir Bar Sorptive Extraction (SBSE), Thermal Desorption-Gas Chromatography-Mass Spectrometry (TD-GC-MS), and the Hydro-CARB^®^ software package (IFP, Rueil-Malmaison, France) was used. This sensitive method allowed for detailed analysis of about 200 individual hydrocarbons at low concentrations. The SBSE methodology is a new, convenient, and contaminant-free technique that permits gas chromatographic analysis of organic compounds from aqueous matrices faster than conventional techniques, thereby circumventing time-costly preparation steps and the use of solvents [4]. The Twister^®^ bars used in this method are magnetic stir bars coated with PolyDiMethylSiloxane (PDMS) [5]. To initiate extraction, 30 mL aliquots of hydrothermal plume samples were transferred from sterile bottles to glass vials, after which the clean Twister^®^ bars were added and used to stir the samples for 60 min. The Twisters® were then removed, dried, and stored in glass vials with helium until analysis with TD-GC-MS. Further details regarding this extraction technique have been published elsewhere [4]. All of the glassware used throughout the process was first combusted at 400°C for 4 h to remove any traces of organic matter.

Hydrothermal sediments and sulfide chimney samples were frozen at -20°C onboard the ship and then transferred to a -80°C freezer upon return to the laboratory. Prior to chemical extraction, the sediments or sulfide deposits were freeze-dried with a trap temperature of −98°C. Next, 10 g of freeze-dried sediments or sulfide deposits were placed in a Soxhlet extractor and extracted with 300 mL of an acetone and cyclohexane solvent mixture (15:85 v/v) at 60°C for 24 h. The extract was then dehydrated using anhydrous sodium sulfate and concentrated to 1–2 mL using a vacuum rotary evaporator. Cleaning of sulfur was conducted according to the United States Environmental Protection Agency (USEPA) method 3660B, while florisil clean-up was conducted using method 3620B (https://www.epa.gov/). Extractions and subsequent quantifications were both conducted in triplicate.

Quantification of alkanes was conducted according to USEPA method 1663, while method 8270D was used for PAH quantification. Identification and quantification of alkane and PAH compounds was conducted on a GC-MS system (Shimadzu QP2010) equipped with a GC column (DB-5ms 30 m long, 0.25 mm internal diameter, 0.25 mm coating). High purity helium was used as the carrier gas for GC-MS analysis with a total flow of 9 mL min^-1^ and an on-column flow of 1.30 mL min^-1^. The split ratio was set at 3.0 and the solvent cut time was 4.0 min. The initial oven temperature was 80°C, but this was increased to 280°C at a rate of 10°C min^-1^ which was then held for 5 min. An electron ionization value of 70 eV was used, while the injector and interface temperatures were maintained at 280°C and 200°C, respectively, and the temperature of the ion source was 250°C. The ion monitoring mode was used for alkane and PAH quantification, while full-scan mode was used for alkane or PAH identification using the GC-MS Postrun Analysis software package (Version 2.10, Shimadzu). Quantification of hydrocarbons was conducted using the external standard method with a series of dilutions for 20 alkane or PAH standard mixtures. Reagent blanks were also analyzed to assess interference caused by condensed solvent. To assess recovery efficiency, parallel experiments were conducted by adding specific concentrations of alkane and PAH mixtures into freeze-dried sediment and sulfide samples, respectively. The recovery ratio of each alkane or PAH was then calculated after Soxhlet extraction and GC-MS quantification.

**Enrichment of hydrocarbon-degrading bacteria at high pressure**

Deep sea-mimicking cultivation was conducted under high and low temperatures in the chamber of a HP vessel (maximum HP 80 MPa, Nantong Feiyu Oil Science and Technology Exploitation, China) (Fig.S6). Flexible, sealed polyethylene terephthalate (PET) plastic bottles were used to contain liquid cultures. All of the PET bottles were repeatedly disinfected with alcohol (75%, v/v) to minimize contamination between experiments. To test for gas leaks and the preservation of medium fluid in the PET cultivation vessels, 250 mL of NH medium containing 5% blue ink and 100 mL of air were placed in 350 mL bottles with about 21 mL of O_2_ and then incubated at 50 MPa for 10, 20, and 40 d at temperatures ranging from 4°C to 30°C. No signs of ink or atmospheric leakage from the bottles occurred, indicating that they were watertight. Enrichment culture pressures were adjusted as follows according to the corresponding water sampling depths: 20 MPa for the plume, 30 MPa for the hydrothermal sulfide deposits, and 35 MPa for the sediment enrichments.

After incubation for 1–2 weeks at 10°C, 50 mL of plume enrichments from the SMAR vent site were transferred into 200 mL of fresh NH medium (with^13^C-labeled octane 0.1 mL, decane 0.1 mL, dodecane 0.1 mL, hexadecane 0.1 mL, eicosane 0.15 g, tetracosane 0.15 g, phenanthrene 0.15 g, naphthalene 0.15 g, pyrene 0.15, fluoranthene 0.15 g, and benzo[α]pyrene 0.15 g as the carbon source) in the PET bottles, then incubated at 10°C under 20 MPa for 20 d. Transfer of enriched cultures (2% v/v) was then repeated twice every 20 d for 60 d total. Controls without inoculum were also conducted in parallel. Five hydrocarbon-degrading consortia were obtained from the enrichments and designated as SAP-1 to SAP-5 (following the hydrothermal plume sample names).

For hydrothermal sulfide deposit enrichments, about 5.0 g of the sample were added to 250 mL NH medium in PET bottles and supplemented with ^13^C-labeled octane 0.1 mL, decane 0.1 mL, dodecane 0.1 mL, hexadecane 0.1 mL, eicosane 0.15 g, tetracosane 0.15 g, phenanthrene 0.15 g, naphthalene 0.15 g, pyrene 0.15 g, fluoranthene 0.15 g, and benzo[α]pyrene 0.15 g, followed by incubation at 30 MPa for 20 d at 10°C. Enrichment cultures were then transferred (2% v/v) every 20 d for a total of 60 d. Three hydrocarbon-degrading consortia were obtained from the sulfide deposit samples collected from each of the three sites (EPR (i.e., EPHCS), SWIR (i.e., WSIP), and SMAR (i.e., SAHCS; Table S1).

PAH was used as the sole carbon and energy source in sediment enrichments because alkanes were not identified in these samples. For each enrichment, about 5.0 g of sediment was added to 250 mL of NH medium in a PET bottle, after which ^13^C-labeled phenanthrene 0.25 g, naphthalene 0.25 g, pyrene 0.25 g, fluoranthene 0.25 g and benzo[α]pyrene 0.25 g were added as the sole carbon and energy sources. The solvents were allowed to evaporate on a rotary shaker for 24 h prior to addition of sediments or inoculum. The basal NH medium was sterilized at 121°C for 20 min, after which trace elements and MgSO_4_ were added separately. Enrichment was conducted under 35 MPa at 10°C for two months. Enrichment cultures were transferred (5% v/v) to fresh medium every two months for a total of six months. Five PAH-degrading consortia were ultimately obtained from the sediment enrichments and designated as S4, S7, S8, S21, and S35, following their respective samples (Table S1). S4 and S21 originated from SMAR, while S7 and S8 originated from SWIR, and S35 originated from EPR.

**Stable-isotope probing experiments**

Stable-isotope probing (SIP) experiments for the above plume, sulfide deposit, and sediment enrichments were performed with ^13^C-labelled alkanes and PAHs, yielding a total of 12 samples. Incubations containing solely ^13^C-unlabeled substrate were run in parallel to act as unlabeled controls. Total DNA from labelled and unlabeled incubations from each of the three SIP experiments was extracted as previously described [6]. Extracted labelled and unlabeled DNA (1.0 to 1.5 µg for each sample) was separated using CsCl gradient ultracentrifugation [7]. To prepare these previously processed SIP samples for 16S rRNA gene sequencing, DNA concentrations were measured using a Qubit 3.0 Fluorometer and a final concentration of 10 ng·µl^-1^ for each sample from the heavy-labeled fraction was used for paired-end Illumina (HiSeq 2000) sequencing.

**Isolation of** **heterotrophic hydrocarbon-degrading bacteria**

Serial dilutions of enrichments were streaked onto M2 agar plates and then incubated at 15°C until the formation of bacterial colonies was observed. Colonies exhibiting unique morphological features were selected and re-streaked onto M2 plates to obtain pure cultures that were then preserved at −20°C for further analyses.

**Cultivation and isolation of chemoautotrophs from** **hydrocarbon-degrading consortia**

Cultures and isolates were identified as previously described [8, 9], with the following modifications. The above hydrocarbon enrichment consortia was diluted to extinction (3 cells/well) in two 96 well Teflon plates (Sonoma Testing, Santa Rosa, CA, USA) using a high throughput culture method with NH medium. One 96-well plate was enriched with 1 mM thiosulfate (S_2_O_3_^2−^) while another that only contained NH medium was used as a control. Growth in each well was measured every 7 d during incubation at 10°C by first transferring 150 μL aliquots of culture into the 96-well polycarbonate plates (Millipore, Billerica, MA, USA) and then staining each well with SYBR Green I (Invitrogen, Carlsbad, CA, USA) diluted in TRIS buffer pH 7.4 at a final concentration of 1/1000. Cell concentrations in 96-well polycarbonate plates were measured using an EasyFlow Guava flow cytometer equipped with a 96-well plate reader (Millipore).

The isolation method used in this work was based on the dilution-to-extinction approach [10-14]. Briefly, isolates were transferred to 250 mL acid-washed polycarbonate flasks (10% HCl) and grown to the early stationary phase (approx. 106 cells/mL), then collected on sterile Supor-200 0.2 μm polyethersulfone filters (Pall, Port Washington, NY, USA). Bacteria were then identified by amplifying and sequencing the 16S rRNA gene with the 27F and 1492R primer set.

**Hydrocarbon degradation by consortia under high hydrostatic pressures and low temperature**

The consortia identified above were inoculated (1.0 mL) in triplicate into 350 mL PET bottles containing 250 mL of NH medium supplemented with alkanes and the PAHs mixture or only the PAHs mixture. Uninoculated control treatments were set in parallel. Importantly, preliminary experimental results indicated that PET bottles could absorb petroleum hydrocarbons, but were not permeable to some components under high hydrostatic pressure and low temperature (data not shown). Cultures were incubated at 10°C in a high-pressure vessel. The residual hydrocarbons were quantified at 0, 20, 40, and 60 d for alkanes+ PAHs enriched consortia, or at 0, 30, 60, and 90 d for PAH-enriched consortia. The culture was then extracted with 100 mL dichloromethane, acidified to pH 2, and then re-extracted using ethyl acetate as the solvent. Neutral and acidic extracts were dried using Na_2_SO_4_, then concentrated to a final volume of 1 mL using a vacuum. Neutral extracts were used to obtain the saturated fraction (SF), aromatic fraction (AF), and total petroleum hydrocarbons (TPHs) prior to GC-MS analysis. Abiotic controls without bacterial inoculation were conducted in parallel, and all treatments were performed in triplicate. The percentages of hydrocarbons removed were normalized to the total extractable matter and abiotic losses removed.

**Hydrocarbon degradation by isolates under high hydrostatic pressures and low temperatures**

To confirm the capacity of bacterial isolates to degrade alkanes under high HPs and low temperatures, approximately 2×10^9^ cells were inoculated into 150 mL NH medium containing 500 μL of ^13^C-labeled hexadecane-1,2-^13^C_4_ in a PET bottle with filter-sterilized air containing 21% O_2_. A single colony of each isolate was then inoculated into 30 mL of M2 liquid medium and incubated at 25°C overnight. The cells were then collected and washed twice with sterile NH medium to remove any remaining carbon sources. After inoculation, incubation was conducted at 10°C under 30 MPa for 20, 40, and 60 d.

Supplementation of ^13^C-labeled PAH (0.5 g) was added to 150 mL of NH medium to assess the activity of PAH-degrading bacterial isolates. The labeled PAHs included phenanthrene-^13^C_6_ for all of the isolates and naphthalene-^13^C_6_, pyrene-^13^C_6_, fluorene-^13^C_6_, and benzo[α]pyrene-^13^C_8_ for strain S21-N3. PAH-activity incubations were performed under 30 MPa and sampled for analysis at 60, 90, and 120 d. Parallel controls without inoculum were also prepared. All of the treatments were performed in triplicate. Growth was detected by measuring optical densities at 600 nm (OD_600_) and by colony counting on plates. Cells were subsequently collected and washed three times with NH medium to remove unincorporated ^13^C-labeled hydrocarbons. Cells were then air-dried and subjected to carbon (δ^13^C) isotopic analysis according to previously described methods [15].

**Genomic DNA extraction and bacterial 16S rRNA gene sequencing**

Total DNA from microbial consortia was extracted using a modified version of previously described methods. A 3 mL subsample of each consortium community was centrifuged, and cell pellets were resuspended in 120 μL 1× TE (10 mM Tris pH 7.5, 1 mM EDTA), then incubated for 1 h. After the addition of 80 μL of 10% sodium dodecyl sulfate (SDS), samples were incubated at 65°C for 1.5 h. DNA extraction was then performed using a QIANamp DNA Mini Kit (QIAGEN, Germany).

To prepare genomic DNA from isolates, cells were grown overnight and harvested by centrifugation, then extracted using an Easypure Bacteria Genomic DNA Kit (TranSgen BioTech, China). Next, the 16S rRNA genes were amplified from the isolate genomic DNA using the universal bacterial PCR primer set 27f (forward; 5′-AGAGTTTGATCCTGGCTCAG-3′) and 1502r (reverse; 5′-ACGGCTACCTTGTTACGACT-3′). PCR thermal cycling parameters comprised a 5 min hot start step at 95°C, followed by 32 cycles with denaturation for 1 min at 94°C, annealing at 55°C for 1 min, extension for 1.5 min at 72°C, and a final extension of 20 min at 72°C. PCR products were then purified using an Omega Cycle-Pure PCR purification kit. Gene sequences were determined from the PCR products using the conserved bacterial 16S rDNA sequencing primers provided by Shanghai Invitrogen and Sanger sequencing on an ABI model 3730 DNA sequencer.

The 16S rDNA sequences obtained were analyzed using the CHIMERA CHECK program (RDPII) [16] to identify and remove chimeric sequences. Non-chimeric sequences were compared against the SILVA 16S rRNA gene database using the SINA web aligner to determine the closest taxonomic neighbor and align the sequences with the most closely related sequences according to the Silva tree server (<http://www.arb-silva.de/>aligner) [17].

**Analysis of bacterial community structures**

Barcoded Illumina paired-end (PE) sequencing (BIPES) was used to obtain high-throughput V3 hypervariable region sequences of 16S rRNA genes by using overlapping PE reads on the Illumina HiSeq 2000 platform, as described previously [18]. Briefly, the 16S rRNA gene V3 tags of each sample were amplified with a barcoded primer, and all of the PCR products from the samples were pooled for PE sequencing. A 10-digit error-correcting barcode was used as described by Zhou et al. [18]. The forward primer was a 25-bp barcode, primer 338f (5’ ACTCCTACGGGAGGCAGCA3’), which included a barcode sequence that was specific to each sample. This was used in conjunction with the reverse primer 806r (5’ GGACTACHVGGGTWTCTAA 3’). A high-fidelity Taq DNA Polymerase (Transgen Biotech, Beijing, China) PCR mixture was used to amplify 16S rRNA gene V3 tags. The PCR procedure included an initial step of 6 min at 95°C, followed by 20 cycles of 1 min at 95°C, 45 s at 65°C, and 1 min at 72°C, with a touchdown of 0.5°C per cycle. This was followed by 16 cycles of 1 min at 95°C, 45 s at 55°C, and 1 min at 72°C. Lastly, a final extension at 72°C was conducted for 10 min.

The barcode-tagged 16S rRNA gene V3-V4 PCR products were pooled and sequenced on a HiSeq 2000 at the Beijing Genomics Institute (Shenzhen, China). The pooled sample was purified using a QIAquick PCR Purification Kit (Qiagen, Hilden, Germany), after which the pooled DNA was end-repaired, A-tails were added, and PE-adapters were ligated using a Paired-end Library Preparation Kit (Illumina, San Diego, CA, USA). Following adapter ligation, the sample was purified and dissolved in 30 μL elution buffer and a 1 mL subsample was used as a template for 15 cycles of PCR amplification. The resultant PCR product was gel purified using the QIAquick Gel Extraction Kit (Qiagen) and sequenced using the 151-bp PE strategy on an Illumina HiSeq 2000 according to the manufacturer’s instructions. The base-calling pipeline provided by the manufacturer (Sequencing Control Software, SCS; Illumina) was used to process raw fluorescent images and identify sequence reads.

**Bioinformatics analyses**

Illumina 16S rRNA gene sequence reads were binned according to index sequences using the PANDA-seq custom algorithm [19]. Overlapping regions of paired-end reads were then aligned to generate contigs. Next, paired-end sequences with mismatches in the assembly were discarded, as were all of the sequences with ambiguous base calls. Chimeras were identified using the ChimeraSlayer algorithm in UCHIME [20], then removed at the OTU clustering step.

All of the sequences (Illumina- and Sanger-based) were assigned taxonomic affiliations using the Naive Bayesian classification as implemented in the RDP classifier [21] with an assignment cutoff threshold of 0.5. Assembled contigs were also subjected to modified single-linkage clustering into operational taxonomic units (OTUs) using CD-HIT [22]. The Good’s coverage estimate [23] was calculated for each of the resulting libraries to estimate the sequence coverage of diversity for the composite Alert library (AT).

**Supplementary References**

1. Wankel SD, Joye SB, Samarkin VA, Shah SR, Friederich G, Melas-Kyriazi J, et al. New constraints on methane fluxes and rates of anaerobic methane oxidation in a Gulf of Mexico brine pool via in situ mass spectrometry. Deep Sea Res Part II. 2010;57: 2022–9.

2. Wang L, Wang W, Lai Q, Shao Z. Gene diversity of CYP153A and AlkB alkane hydroxylases in oil‐degrading bacteria isolated from the Atlantic Ocean. Environmental Microbiology. 2010;12(5):1230-42.

3. Wang B, Lai Q, Cui Z, Tan T, Shao Z. A pyrene‐degrading consortium from deep‐sea sediment of the West Pacific and its key member Cycloclasticus sp. P1. Environmental Microbiology. 2008;10(8):1948-63.

4. Konn C, Charlou J-L, Donval J-P, Holm N, Dehairs F, Bouillon S. Hydrocarbons and oxidized organic compounds in hydrothermal fluids from Rainbow and Lost City ultramafic-hosted vents. Chemical Geology. 2009;258(3):299-314.

5. Baltussen E, Sandra P, David F, Janssen H-G, Cramers C. Study into the equilibrium mechanism between water and poly (dimethylsiloxane) for very apolar solutes: Adsorption or sorption? Analytical Chemistry. 1999;71(22):5213-6.

6. Dombrowski N, Donaho JA, Gutierrez T, Seitz KW, Teske AP, Baker BJ. Reconstructing metabolic pathways of hydrocarbon-degrading bacteria from the Deepwater Horizon oil spill. Nat Microbiol. 2016;1(7):16057.

7. Gutierrez T, Singleton DR, Berry D, Yang T, Aitken MD, Teske A. Hydrocarbon-degrading bacteria enriched by the Deepwater Horizon oil spill identified by cultivation and DNA-SIP. ISME J. 2013;7(11):2091-104.

8. Shah V, Chang BX, Morris RM. Cultivation of a chemoautotroph from the SUP05 clade of marine bacteria that produces nitrite and consumes ammonium. ISME J. 2017;11(1):263-71.

9. Mattes TE, Nunn BL, Marshall KT, Proskurowski G, Kelley DS, Kawka OE, et al. Sulfur oxidizers dominate carbon fixation at a biogeochemical hot spot in the dark ocean. ISME J. 2013;7(12):2349-60.

10. Oueriaghli N, Castro DJ, Llamas I, Béjar V, Martínez-Checa F. Study of bacterial community composition and correlation of environmental variables in Rambla Salada, a hypersaline environment in south-eastern Spain. Frontiers in microbiology. 2018;9.

11. Colin Y, Goñi-Urriza M, Gassie C, Carlier E, Monperrus M, Guyoneaud R. Distribution of sulfate-reducing communities from estuarine to Marine Bay Waters. Microbial ecology. 2017;73(1):39-49.

12. Button D, Schut F, Quang P, Martin R, Robertson BR. Viability and isolation of marine bacteria by dilution culture: theory, procedures, and initial results. Appl Environ Microbiol. 1993;59(3):881-91.

13. Connon SA, Giovannoni SJ. High-throughput methods for culturing microorganisms in very-low-nutrient media yield diverse new marine isolates. Appl Environ Microbiol. 2002;68(8):3878-85.

14. Yang S-J, Kang I, Cho J-C. Expansion of cultured bacterial diversity by large-scale dilution-to-extinction culturing from a single seawater sample. Microbial ecology. 2016;71(1):29-43.

15. Wang W, Shao Z. The long-chain alkane metabolism network of Alcanivorax dieselolei. Nature communications. 2014;5.

16. Cole JR, Chai B, Marsh TL, Farris RJ, Wang Q, Kulam SA, et al. The Ribosomal Database Project (RDP-II): previewing a new autoaligner that allows regular updates and the new prokaryotic taxonomy. . Nucleic acids research. 2003; 31(1):442-3.

17. Pruesse E, Quast C, Knittel K, Fuchs BM, Ludwig W, Peplies J, et al. SILVA: a comprehensive online resource for quality checked and aligned ribosomal RNA sequence data compatible with ARB. Nucl Acids Res. 2007;35:7188–96.

18. Zhou H-W, Li D-F, Tam NF-Y, Jiang X-T, Zhang H, Sheng H-F, et al. BIPES, a cost-effective high-throughput method for assessing microbial diversity. The ISME Journal. 2011;5(4):741-9.

19. Bartram AK, Lynch MD, Stearns JC, Moreno-Hagelsieb G, Neufeld JD. Generation of multimillion-sequence 16S rRNA gene libraries from complex microbial communities by assembling paired-end Illumina reads. Applied and environmental microbiology. 2011;77(11):3846-52.

20. Edgar RC, Haas BJ, Clemente JC, Quince C, R. K. UCHIME improves sensitivity and speed of chimera detection. Bioinformatics. 2011;27(2194–2200).

21. Wang Q, Garrity GM, Tiedje JM, Cole JR. Naive Bayesian classifier for rapid assignment of rRNA sequences into the new bacterial taxonomy. Applied and environmental microbiology. 2007;73(16):5261-7.

22. Li W, Godzik A. Cd-hit: a fast program for clustering and comparing large sets of protein or nucleotide sequences. Bioinformatics. 2006;22(13):1658-9.

23. Good IJ. The population frequencies of species and the estimation of population parameters. Biometrika. 1953;40(3-4):237-64.
