# Supplementary material for: The oxidation of hydrocarbons by diverse heterotrophic and mixotrophic bacteria that inhabit deep-sea hydrothermal ecosystems
Source: ISME J. 2020 Apr 30;14(8):1994–2006. doi: 10.1038/s41396-020-0662-y (PMC7368058; doi:10.1038/s41396-020-0662-y)
Supplement: Supplementary file 2 — Supplementary Information [file 41396_2020_662_MOESM2_ESM.pdf]

## Supplementary Figure legends

**Supplementary Figure 1.** Location of sampling sites of deep-sea hydrothermal vents. Red dots indicate sampling sites; arrow head points to scientific investigation of the sea area.

**Supplementary Figure 2.** The $\delta^{13}$C values of various chemoautotroph isolates grown on $^{13}$C-labeled hydrocarbons including octane-$^{13}C_2$, decane-$^{13}C_2$, dodecane-$^{13}C_4$, hexadecane-$^{13}C_4$, phenanthrene-$^{13}C_6$, naphthalene-$^{13}C_6$, as their sole carbon source under high hydrostatic pressures and low temperatures. Detailed information for the above strains is provided in Table 1.

**Supplementary Figure 3.** Growth of various hydrocarbon-degrading strains under high hydrostatic pressures and low temperatures. The $\delta^{13}$C values (A) and $OD_{600}$ values (B) of various alkane-degrading strains grown on hexadecane-1, 2-$^{13}C_4$ as their sole carbon source. The $\delta^{13}$C value (C) and $OD_{600}$ value (D) of various PAH-degrading strains grown on phenanthrene-$^{13}C_6$ as their sole carbon source. The $\delta^{13}$C value (E) and $OD_{600}$ value (F) of strain S21-N3 grown on naphthalene-$^{13}C_{6,}$ phenanthrene-$^{13}C_{6,}$ pyrene-$^{13}C_{6,}$ fluorene-$^{13}C_6$, and benzo[α]pyrene-$^{13}C_8$ as its sole carbon sources. Detailed information for the above strains is provided in Supplementary Table S5. Data are presented as the mean of three independent experiments. Error bars represent the S.D. Nap, naphthalene-$^{13}C_6$; Phe, phenanthrene-$^{13}C_6$; Pyr, pyrene-$^{13}C_6$; Flu, fluoranthene-$^{13}C_6$; and B[a]P, benzopyrene-$^{13}C_8$.

**Supplementary Figure 4.** The $\delta^{13}$C value (A) and $OD_{600}$ value (B) test of strain S7-N8 grown on naphthalene-$^{13}C_{6}$, phenanthrene-$^{13}C_{6}$, pyrene-$^{13}C_{6}$, fluorene-$^{13}C_{6}$, and benzo[α]pyrene-$^{13}C_{8}$ as the sole carbon sources under high hydrostatic pressures and low temperature, respectively. Data are presented as the mean of three independent experiments. Error bars represent the S.D. Nap,naphthalene-$^{13}C_{6}$; Phe,phenanthrene-$^{13}C_{6}$; Pyr, pyrene-$^{13}C_{6}$; Flu, fluoranthene-$^{13}C_{6}$ ; and B[a]P, benzopyrene-$^{13}C_{8}$ .

**Supplementary Figure 5.** SAR202 distribution among consortia and phylogenetic analysis of 16S rRNA gene phylotypes. Phylogenetic tree showing the diversity of 16S rRNA gene sequences from OTUs of SAR202 dentified in this study. The tree was constructed using neighbor-joining (NJ) methods and the Kimura 2-parameter model, as implemented in the MEGA 5.0 software package. The tree is based on partial 16S rRNA gene sequences from this study and their closest type strains. Only bootstrap values ≥ 50% (based on 1,000 bootstrap replicates) are shown at the nodes. The scale bar represents 0.05 nucleotide changes per site. SAR202 OTU distribution among the different hydrocarbon-enrichment consortia and indigenous consortias. Only OTUs representing > 1% of the communities in at least one sample are included in the visualization. OTUs representative sequence are shown in supplementary materials.

**Supplementary Figure 6.** Schematic illustration of the microbiological cultivation and incubation technique under a high hydrostatic pressure and low temperature.

# Supplementary Figure 1

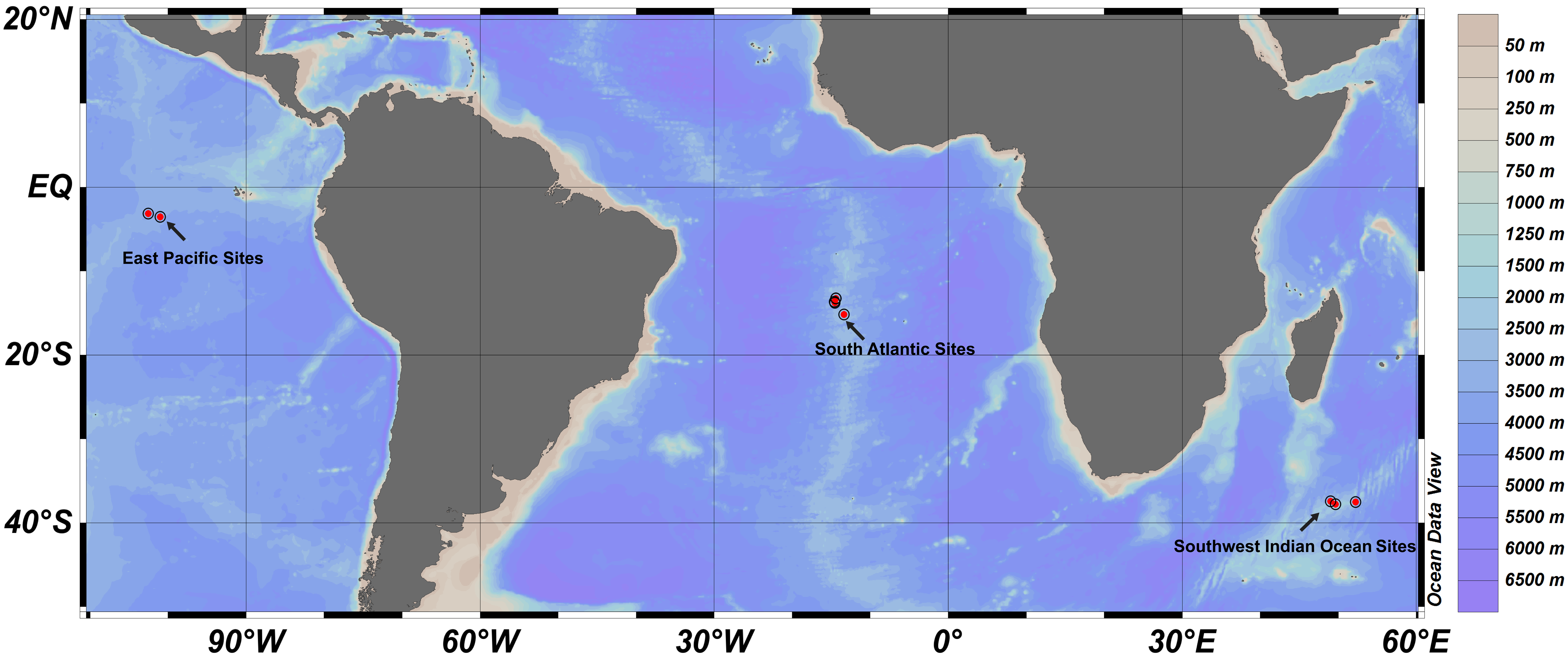

Supplementary Figure 2

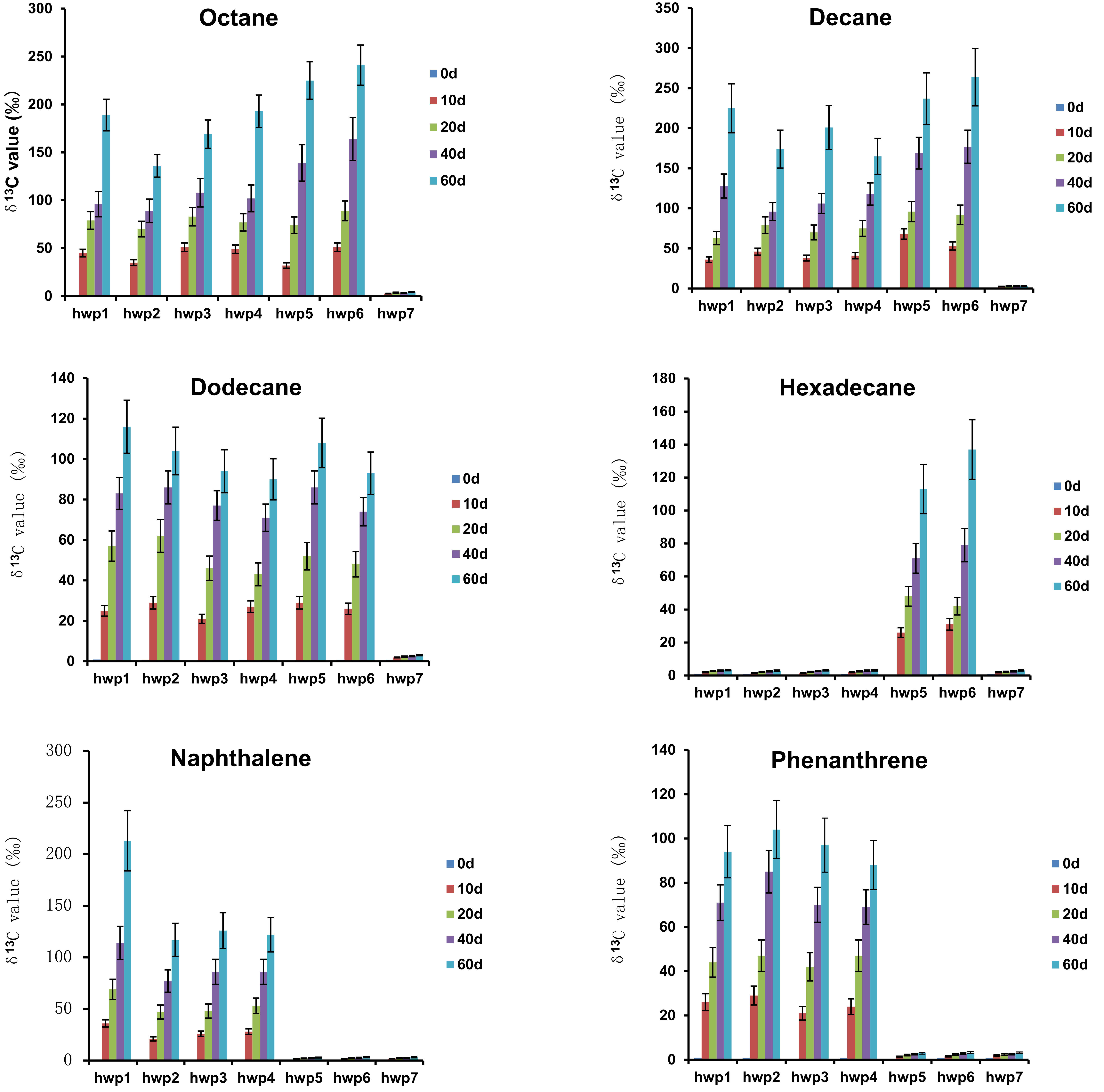

Supplementary Figure 3

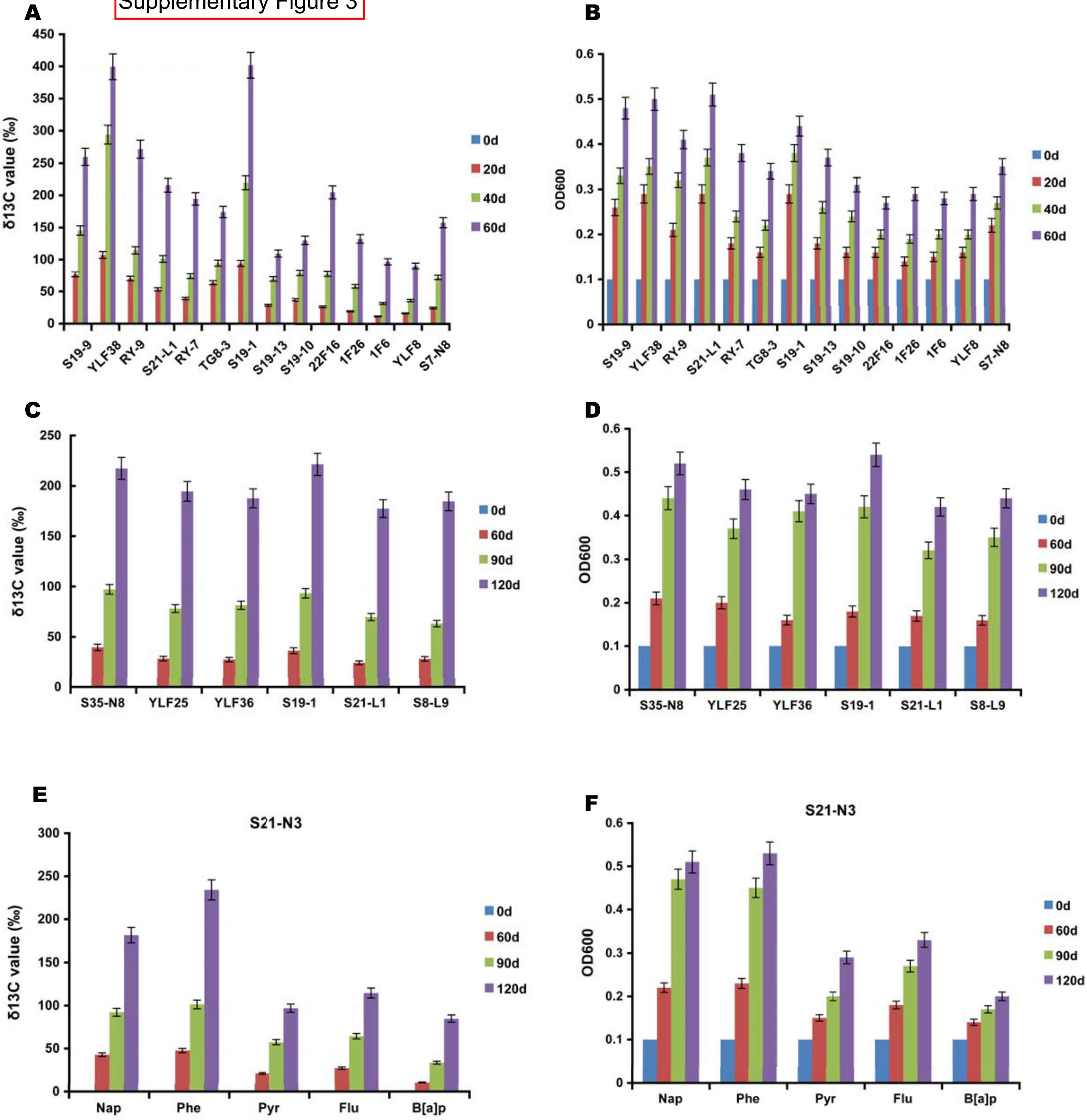

Supplementary Figure 4

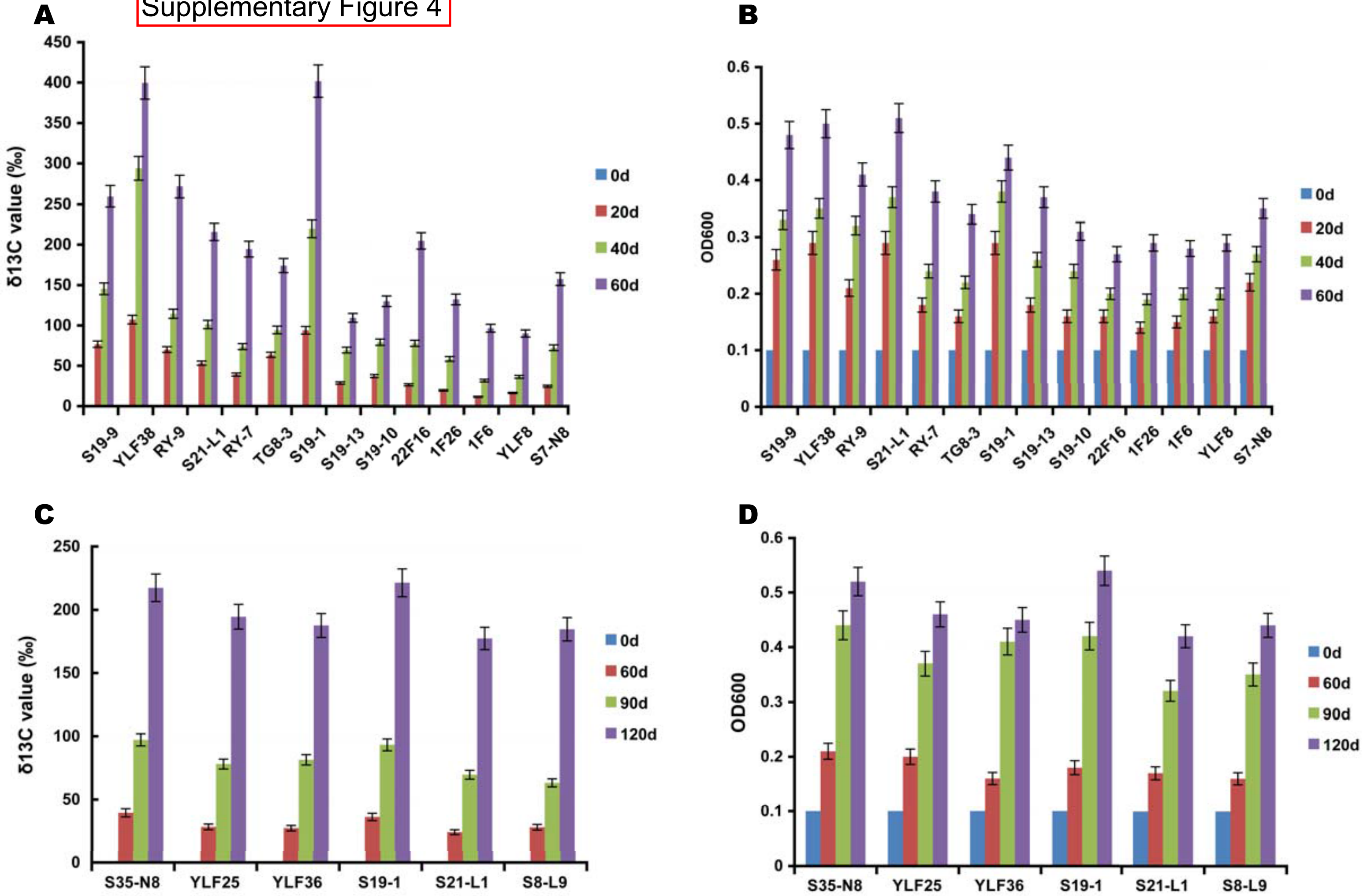

Supplementary Figure 5

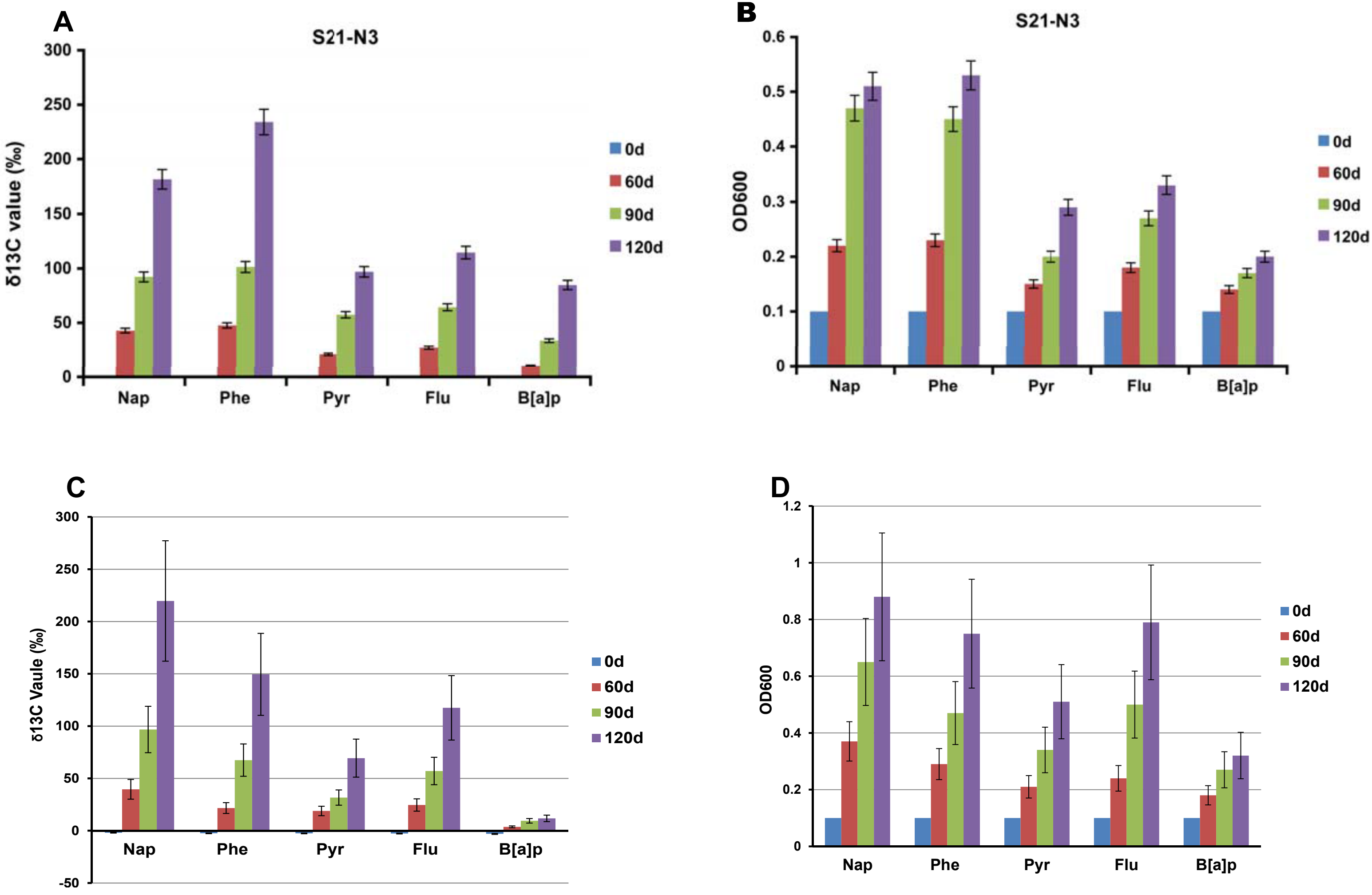

**1.Cultivation flexible sealed polyethylene terephthalate(PET) plastic bottles**

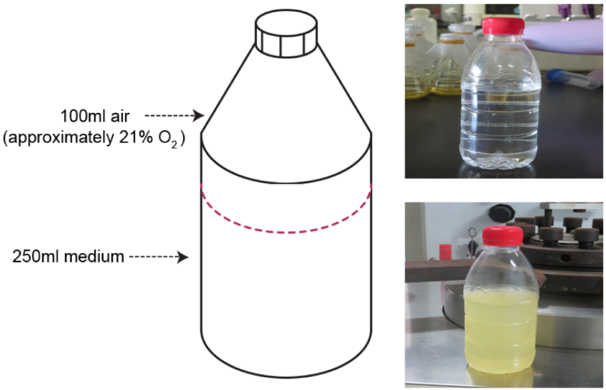


**2.Hydrostatic pressure vessel**

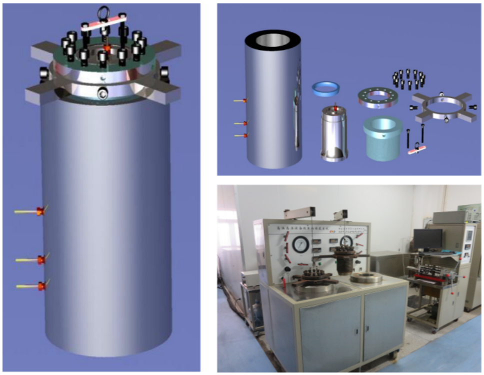

## Deep Sea Bacterial Culture System

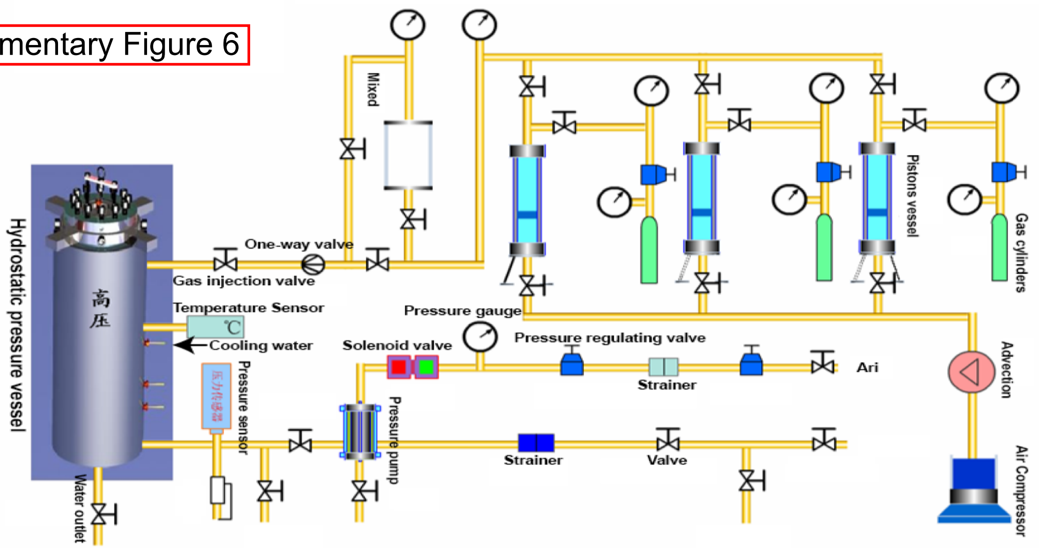


Supplementary Figure 6

**Supplementary Table 1 Features of the samples used in this work**

| *Sample* | *Location* | *Area* | *Sample type* | *Depth(m )* | *Tempera ture ( ℃)* | *Salinity(PSU)* | *Turbidity (FTU)* | *Dissolved oxygen(mg\L)* | *Pressure (db)* |
|---|---|---|---|---|---|---|---|---|---|
| SAP-1_S | 14.4 °(W); 13.2 °(S) | South Atlantic | Hydrothermal rising plume | 2334 | 9.3 | 34.896 | 0.549 | 7.94 | 2332 |
| SAP-2_S | 14.4 °(W); 13.2 °(S) | South Atlantic | Hydrothermal neutrally buoyant plume | 2312 | 8.8 | 34.623 | 0.557 | 7.63 | 2311 |
| SAP-3_S | 14.4 °(W); 13.2 °(S) | South Atlantic | Hydrothermal neutrally buoyant plume | 2293 | 8.5 | 34.726 | 0.567 | 7.81 | 2291 |
| SAP-4_S | 14.4 °(W); 13.2 °(S) | South Atlantic | Hydrothermal neutrally buoyant plume | 2261 | 8.1 | 34.88 | 0.574 | 7.41 | 2260 |
| SAP-5_S | 14.4 °(W); 13.2 °(S) | South Atlantic | Hydrothermal neutrally buoyant plume | 2273 | 7.4 | 34.48 | 0.581 | 7.49 | 2271 |
| SMAR | 14.5 °(W); 13.8 °(S) | South Atlantic | Hydrothermal sulfide (active sites) | 2933 | 9.7 | ND | ND | ND | 2930 |
| EPR | 102.5 °(W); 3.2 °(S) | East Pacific | Hydrothermal sulfide (active sites) | 3494 | 8.6 | ND | ND | ND | 3493 |

| | | | | | | | | | |
|---|---|---|---|---|---|---|---|---|---|
| SWIP | 49.6 °(E); 37.8 °(S) | Southwest Indian | Hydrothermal sulfide(active sites) | 3126 | 4.3 | ND | ND | ND | 3124 |
| S4_S | 14.6 °(W); 13.6 °(S) | South Atlantic | Hydrothermal sediments | 3751 | 4.8 | ND | ND | ND | 3749 |
| S7_S | 49.0 °(E); 37.4 °(S) | Southwest Indian Ocean | Hydrothermal sediments | 3282 | 5.2 | ND | ND | ND | 3280 |
| S8_S | 52.2 °(E); 37.5 °(S) | Southwest Indian Ocean | Hydrothermal sediments | 3928 | 3.0 | ND | ND | ND | 3927 |
| S21_S | 13.3 °(W); 15.2 °(S) | South Atlantic | Hydrothermal sediments | 3271 | 7.7 | ND | ND | ND | 3270 |
| S35_S | 101.0 °(W); 3.5 °(S) | East Pacific Ocean | Hydrothermal sediments | 3486 | 6.3 | ND | ND | ND | 3484 |

ND, not determined.

## Supplementary Table 2 Various Hydrocarbons detected in deep sea vent samples[a]

| Compounds\ Formula | Deep seawater[b] | SAP-1_S[b] | SAP-2_S[b] | SAP-3_S[b] | SAP-4_S[b] | SAP-5_S[b] | SMAR[c] | EPR[c] | SWIR[c] | S8_S[c] | S7_S[c] | S21_S[c] | S4_S[c] | S35_S[c] |
|---|---|---|---|---|---|---|---|---|---|---|---|---|---|---|
| ***n*-alkane** | | | | | | | | | | | | | | |
| **Heptane\ $C_7H_{16}$** | **nd** | **43.6 ± 8.55** | **38.3 ± 8.27** | **31.8 ± 6.30** | **30.6 ± 6.07** | **29.5 ± 5.84** | **nd** | **nd** | **nd** | **nd** | **nd** | **nd** | **nd** | **nd** |
| **Nonane\ $C_9H_{20}$** | **nd** | **44.5 ± 8.68** | **46.2 ± 9.03** | **33.3 ± 5.29** | **32.6 ± 8.63** | **31.0 ± 7.78** | **nd** | **nd** | **nd** | **nd** | **nd** | **nd** | **nd** | **nd** |
| **Decane\ $C_{10}H_{22}$** | **7.6 ± 0.39** | **40.1 ±** 6.67 | **34.5 ± 9.05** | **35.1 ± 8.91** | **32.0 ± 7.45** | **29.9 ± 5.96** | **nd** | **nd** | **nd** | **nd** | **nd** | **nd** | **nd** | **nd** |
| **Undecane\ $C_{11}H_{24}$** | **nd** | **27.3 ± 7.03** | **26.4 ± 7.01** | **28.8 ± 7.45** | **27.5 ± 7.07** | **27.4 ± 5.09** | **nd** | **246.24 ± 38.51** | **nd** | **nd** | **nd** | **nd** | **nd** | **nd** |
| **Dodecane\ $C_{12}H_{26}$** | **nd** | **31.6 ± 8.34** | **30.5 ± 8.23** | **33.7 ± 8.04** | **30.8 ± 6.38** | **34.0 ± 9.04** | **203.04 ± 47.82** | **259.2 ± 52.98** | **nd** | **nd** | **nd** | **nd** | **nd** | **nd** |
| **Tridecane\ $C_{13}H_{28}$** | **nd** | **28.7 ± 5.56** | **22.8 ± 5.60** | **23.2 ± 4.56** | **21.5 ± 3.56** | **19.3 ± 6.22** | **254.88 ± 59.32** | **nd** | **nd** | **nd** | **nd** | **nd** | **nd** | **nd** |
| **Tetradecane** | **nd** | **36.1** | **28.1** | **25.6** | **24.5** | **22.7** | **369.36** | **334.8** | **194.75** | **nd** | **nd** | **nd** | **nd** | **94.34** |

| | | | | | | | | | | | | | | |
|---|---|---|---|---|---|---|---|---|---|---|---|---|---|---|
| $C_{14}H_{30}$ | | ± 6.30 | ± 5.56 | ± 6.33 | ± 5.77 | ± 4.03 | ± 93.31 | ± 76.71 | ± 43.61 | | | | | ± 21.86 |
| Pentadecane\ $C_{15}H_{32}$ | nd | 29.4 ± 6.85 | 23.1 ± 4.66 | 24.3 ± 5.21 | 20.9 ± 4.33 | 19.8 ± 3.81 | 397.44 ± 106.36 | 524.88 ± 134.21 | 246 ± 64.34 | nd | nd | nd | nd | nd |
| Hexadecane\ $C_{16}H_{34}$ | nd | 33.5 ± 6.59 | 27.9 ± 6.35 | 28.3 ± 4.99 | 22.4 ± 5.42 | 22.8 ± 4.88 | 788.4 ± 189.05 | 736.56 ± 135.52 | 485.85 ± 94.67 | nd | nd | nd | nd | nd |
| Heptadecane\ $C_{17}H_{36}$ | nd | 32.8 ± 6.53 | 27.0 ± 7.13 | 26.7 ± 4.84 | 24.3 ± 4.60 | 26.7 ± 5.60 | 624.24 ± 166.97 | 548.64 ± 144.83 | 701.1 ± 152.54 | nd | nd | 82.08 ± 15.14 | nd | nd |
| Octadecane\ $C_{18}H_{38}$ | nd | 21.3 ± 3.72 | 19.5 ± 3.77 | 17.5 ± 4.58 | 19.2 ± 3.53 | 17.6 ± 3.88 | 745.2 ± 190.37 | 730.08 ± 168.65 | 727.75 ± 176.30 | 96.12 ± 22.67 | nd | 145.8 ± 34.38 | nd | 108.12 ±26.47 |
| Nonadecane\ $C_{19}H_{40}$ | nd | 16.2 ± 3.98 | 13.1 ± 3.05 | 10.4 ± 1.85 | 9.8 ± 2.18 | 11.2 ± 2.69 | 639.36 ± 138.69 | 591.84 ± 156.10 | 617.05 ± 116.35 | nd | nd | nd | nd | nd |
| Eicosane\ $C_{20}H_{42}$ | nd | 12.4 ± 3.21 | 13.5 ± 2.77 | 11.5 ± 3.02 | 8.8 ± 1.91 | 7.6 ± 1.65 | 596.16 ± 130.20 | 570.24 ± 130.61 | 606.8 ± 159.94 | 220.32 ± 48.29 | 164.8 ± 42.94 | nd | nd | nd |
| Heneicosane\ $C_{21}H_{44}$ | nd | 15.3 ± 3.21 | 16.2 ± 3.00 | 13.7 ± 3.14 | 10.8 ± 1.97 | 9.9 ± 2.48 | 650.16 ± 153.66 | 613.44 ± 111.11 | 584.25 ± 108.34 | nd | nd | nd | 213.92 ± 50.96 | nd |
| Docosane\ $C_{22}H_{46}$ | nd | 11.3 ± 2.70 | 8.4 ± 2.25 | 8.0 ± 1.37 | 6.5 ± 1.49 | 6.7 ± 1.34 | 615.6 ± 110.76 | 635.04 ± 113.69 | 547.35 ± 99.94 | nd | nd | nd | nd | nd |

| | | | | | | | | | | | | | | |
|---|---|---|---|---|---|---|---|---|---|---|---|---|---|---|
| **Tricosane\ $C_{23}H_{48}$** | **nd** | **7.4 ± 1.38** | **5.3 ± 1.07** | **3.7 ± 0.81** | **nd** | **nd** | **494.64 ± 134.06** | **546.48 ± 93.39** | **535.05 ± 116.77** | **nd** | **nd** | **179.28 ± 38.02** | **nd** | **142.04 ± 36.00** |
| **Tetracosane\ $C_{24}H_{50}$** | **nd** | **6.8 ± 1.45** | **5.5 ± 0.95** | **2.8 ± 0.60** | **nd** | **nd** | **527.04 ± 114.08** | **498.96 ± 97.88** | **549.4 ± 103.68** | **nd** | **nd** | **nd** | **nd** | **nd** |
| **Pentacosane\ $C_{25}H_{52}$** | **nd** | **3.4 ± 0.64** | **1.9 ± 0.50** | **nd** | **nd** | **nd** | **382.32 ± 96.34** | **440.64 ± 102.19** | **487.9 ± 112.94** | **86.4 ± 16.91** | **nd** | **nd** | **211.68 ± 55.57** | **nd** |
| **Hexacosane\ $C_{26}H_{54}$** | **nd** | **3.3 ± 0.65** | **2.7 ± 0.55** | **nd** | **nd** | **nd** | **265.68 ± 64.31** | **347.76 ± 83.90** | **291.1 ± 62.56** | **nd** | **114.33 ± 22.76** | **nd** | **nd** | **75.26 ± 16.84** |
| **Heptacosane\ $C_{27}H_{56}$** | **nd** | **1.4 ± 0.37** | **nd** | **nd** | **nd** | **nd** | **289.44 ± 53.35** | **321.84 ± 85.90** | **350.55 ± 63.15** | **nd** | **nd** | **104.76 ± 19.65** | **nd** | **nd** |
| **Octacosane\ $C_{28}H_{58}$** | **nd** | **2.2 ± 0.45** | **nd** | **nd** | **nd** | **nd** | **356.4 ± 79.80** | **226.8 ± 4 5.16** | **241.9 ± 43.61** | **89.64 ± 19.10** | **nd** | **nd** | **180.32 ± 33.88** | **nd** |
| **Branched alkanes** | | | | | | | | | | | | | | |
| **$C_9H_{20}$** | **nd** | **41.7 ± 7.49** | **37.6 ± 7.01** | **38.3 ± 6.60** | **32.9 ± 5.69** | **34.0 ± 7.31** | **95.04 ± 16.95** | **nd** | **nd** | **nd** | **nd** | **nd** | **nd** | **nd** |
| **$C_{10}H_{22}$** | **nd** | **35.2 ± 8.20** | **33.5 ± 7.91** | **32.6 ± 7.58** | **31.7 ± 7.51** | **32.8 ± 6.02** | **311.04 ± 57.62** | **222.48 ± 53.01** | **nd** | **nd** | **nd** | **nd** | **nd** | **nd** |

| | | | | | | | | | | | | | | |
|---|---|---|---|---|---|---|---|---|---|---|---|---|---|---|
| $C_{11}H_{24}$ | nd | 34.1 ± 7.61 | 29.5 ± 5.73 | 27.4 ± 5.41 | 27.7 ± 5.94 | 22.6 ± 4.34 | 514.08 ± 87.96 | nd | 178.35 ± 43.61 | nd | nd | nd | nd | nd |
| $C_{12}H_{26}$ | nd | 28.4 ± 6.75 | 22.9 ± 4.51 | 25.2 ± 5.80 | 19.6 ± 4.31 | 18.5 ± 4.14 | 267.84 ± 57.88 | nd | nd | nd | nd | nd | nd | nd |
| $C_{13}H_{28}$ | nd | 22.7 ± 4.68 | 17.1 ± 2.91 | 15.8 ± 3.45 | 16.2 ± 2.76 | 11.7 ± 3.13 | 172.8 ± 37.91 | nd | nd | nd | nd | nd | nd | nd |
| $C_{15}H_{32}$ | nd | 13.7 ± 2.47 | 9.4 ± 2.28 | 8.6 ± 2.05 | 7.6 ± 1.50 | nd | 196.56 ± 35.65 | 591.84 ± 142.00 | 235.75 ± 44.59 | nd | 141.11 ± 25.75 | nd | nd | 110.24 ± 25.86 |
| $C_{16}H_{34}$ | nd | 12.1 ± 29.94 | 8.6 ± 2.06 | nd | nd | nd | 520.56 ± 108.41 | 568.08 ± 132.46 | 541.2 ± 131.35 | nd | nd | nd | nd | nd |
| $C_{18}H_{38}$ | nd | 7.7 ± 1.33 | 5.2 ± 1.16 | 5.1 ± 1.23 | nd | nd | 481.68 ± 88.42 | 596.16 ± 158.15 | 510.45 ± 103.47 | nd | nd | 168.48 ± 39.48 | nd | nd |
| $C_{19}H_{40}$ | nd | 8.2 ± 1.47 | 7.5 ± 1.59 | nd | nd | nd | 613.44 ± 163.74 | 663.12 ± 137.63 | 463.3 ± 109.66 | nd | nd | nd | 150.08 ± 31.89 | nd |
| $C_{20}H_{42}$ | nd | 6.7 ± 1.36 | nd | 3.4 ± 0.80 | nd | nd | 289.44 ± 74.73 | 531.36 ± 135.95 | nd | 109.08 ± 20.68 | nd | nd | 192.64 ± 51.18 | 178.08 ± 33.12 |
| **Cycloalkanes** | | | | | | | | | | | | | | |

| | | | | | | | | | | | | | | |
|---|---|---|---|---|---|---|---|---|---|---|---|---|---|---|
| **Cyclohexane\ $C_6H_{12}$** | **nd** | **19.3 ± 4.96** | **14.0 ± 3 .60** | **13.7 ± 3.62** | **11.4 ± 2.13** | **10.5 ± 1.98** | **nd** | **nd** | **nd** | **nd** | **nd** | **nd** | **nd** | **nd** |
| **Cyclohexane, butyl-\ $C_{10}H_{20}$** | **nd** | **13.4 ± 3.37** | **12.4 ± 2.89** | **8.6 ± 2.01** | **5.3 ± 0.97** | **6.1 ± 1.17** | **nd** | **nd** | **584.25 ± 113.36** | **nd** | **90.64 ± 20.25** | **nd** | **nd** | **nd** |
| **Cyclohexane, pentyl-\ $C_{11}H_{22}$** | **nd** | **9.1 ± 2.02** | **7.6 ± 1.86** | **nd** | **nd** | **nd** | **248.4 ± 58.98** | **nd** | **nd** | **nd** | **53.56 ± 9.52** | **147.96 ± 34.91** | **nd** | **nd** |
| **Cyclohexane, hexyl-\ $C_{16}H_{32}$** | **nd** | **3.7 ± 0.99** | **2.9 ± 0.58** | **nd** | **nd** | **nd** | **164.16 ± 30.77** | **144.72 ± 28.06** | **118.9 ± 23.71** | **nd** | **nd** | **115.56 ± 29.86** | **nd** | **nd** |
| **Aromatics** | | | | | | | | | | | | | | |
| **Monocyclic** | | | | | | | | | | | | | | |
| **Phenol\ $C_6H_6O$** | **nd** | **5.5 ± 1.39** | **7.6 ± 1.86** | **4.7 ± 1.19** | **5.8 ± 1.35** | **6.6 ± 1.39** | **360.72 ± 95.82** | **181.44 ± 36.85** | **nd** | **nd** | **24.72 ± 6.36** | **36.72 ± 8.38** | **79.52 ± 20.90** | **nd** |
| **Toluene\ $C_7H_8$** | **nd** | **9.1 ± 1.97** | **3.8 ± 0.68** | **nd** | **nd** | **nd** | **246.24 ± 58.99** | **nd** | **nd** | **241.92 ± 54.54** | **220.42 ± 50.10** | **131.76 ± 30.14** | **106.4 ± 19.64** | **nd** |
| **Styrene\ $C_8H_8$** | **nd** | **7.9 ± 1.81** | **nd** | **nd** | **nd** | **nd** | **183.6 ± 38.08** | **nd** | **nd** | **189 ± 43.69** | **130.81 ± 23.03** | **73.44 ± 17.47** | **54.88 ± 11.82** | **nd** |
| **Benzene, alkyl \$C_8H_{10}$** | **2.7 ±** | **nd** | **nd** | **nd** | **nd** | **nd** | **nd** | **nd** | **nd** | **169.56 ±** | **189.52 ±** | **104.76 ±** | **nd** | **80.56 ±** |

| | | | | | | | | | | | | | | |
|---|---|---|---|---|---|---|---|---|---|---|---|---|---|---|
| | 0.54 | | | | | | | | | 43.64 | 41.09 | 28.17 | | 17.64 |
| **Benzene, alkyl\ $C_9H_{12}$** | nd | nd | nd | nd | nd | nd | nd | nd | nd | 149.04 ± 29.65 | 210.12 ± 49.31 | 112.32 ± 26.91 | nd | 99.64 ± 22.94 |
| **Benzene, alkyl\ $C_{10}H_{14}$** | nd | nd | nd | nd | nd | nd | nd | nd | nd | nd | nd | 91.8 ± 21.08 | nd | 51.94 ± 10.46 |
| **Benzene, alkyl\ $C_{12}H_{18}$** | nd | nd | nd | nd | nd | nd | 112.32 ± 2.98 | 174.96 ± 35.08 | nd | nd | 63.86 ± 15.77 | nd | nd | nd |
| **Bicyclic** | | | | | | | | | | | | | | |
| **Naphtalene \ $C_{10}H_8$** | nd | 5.1 ± 0.96 | 4.4 ± 0.78 | 4.1 ± 0.92 | 2.4 ± 0.46 | 2.7 ± 0.61 | 339.12 ± 76.13 | nd | nd | 133.92 ± 26.38 | 146.26 ± 33.77 | 123.12 ± 24.27 | 181.44 ± 43.11 | 155.82 ± 30.69 |
| **Naphtalene, alkyl\ $C_{11}H_{10}$** | nd | 1.3 ± 0.22 | 1.0 ± 0.17 | 1.4 ± 0.35 | 0.7 ± 0.12 | 0.2 ± 0.05 | nd | nd | nd | 92.88 ± 21.19 | 74.16 ± 17.91 | 115.56 ± 21.83 | 62.72 ± 11.47 | 36.04 ± 7.57 |
| **Naphtalene, alkyl\ $C_{12}H_{12}$** | nd | nd | nd | nd | nd | nd | nd | nd | nd | nd | nd | nd | 50.4 ± 14.61 | nd |
| **Naphtalene, alkyl\ $C_{13}H_{12}$** | nd | nd | nd | nd | nd | nd | 71.28 ± 14.70 | nd | nd | nd | nd | 65.88 ± 11.86 | 62.72 ± 11.47 | nd |
| **PAH** | | | | | | | | | | | | | | |
| **Acenaphthene** | nd | 3.1 | nd | nd | nd | nd | 738.72 | 570.24 | 442.8 | 189 | 130.81 | 264.6 | 133.28 | 82.68 |

| | | | | | | | | | | | | | | |
|---|---|---|---|---|---|---|---|---|---|---|---|---|---|---|
| \ $C_{12}H_{10}$ | | ± 0.59 | | | | | ± 198.06 | ± 137.27 | ± 92.09 | ± 47.74 | ± 23.58 | ± 54.31 | ± 31.77 | ± 20.56 |
| **Fluorene\ $C_{13}H_{10}$** | nd | 1.7 ± 0.40 | nd | nd | nd | nd | 596.16 ± 103.32 | 702 ± 129.30 | 623.2 ± 131.79 | 339.12 ± 63.89 | 223.51 ± 38.71 | 192.24 ± 48.50 | 217.28 ± 44.26 | 190.8 ± 40.25 |
| **Anthracene\ $C_{14}H_{10}$** | nd | nd | nd | nd | nd | nd | 317.52 ± 63.40 | 282.96 ± 68.45 | 524.8 ± 117.39 | 314.28 ± 84.50 | 254.41 ± 50.27 | 336.96 ± 70.16 | 255.36 ± 45.27 | 318 ± 58.84 |
| **Phenanthrene \ $C_{14}H_{10}$** | nd | 2.4 ± 0.61 | 1.5 ± 0.27 | nd | 1.8 ± 0.38 | nd | 1008.72 ± 223.03 | 751.68 ± 180.63 | 902 ± 195.22 | 625.32 ± 147.01 | 374.92 ± 86.34 | 321.84 ± 59.21 | 645.12 ± 142.34 | 403.86 ± 83.32 |
| **Fluoranthene\ $C_{16}H_{10}$** | nd | 1.4 ± 0.30 | 2.1 ± 0.49 | 1.6 ± 0.41 | 1.0 ± 0.20 | 0.8 ± 0.21 | 542.16 ± 106.64 | 533.52 ± 126.57 | 432.55 ± 98.24 | 265.68 ± 57.99 | 386.25 ± 87.05 | 330.48 ± 70.27 | 436.8 ± 112.21 | 322.24 ± 66.48 |
| **Pyrene\ $C_{16}H_{10}$** | nd | nd | nd | nd | nd | nd | 224.64 ± 50.74 | 306.72 ± 77.33 | 241.9 ± 42.54 | 382.32 ± 80.71 | 199.82 ± 48.59 | 403.92 ± 108.53 | 153.44 ± 36.14 | 177.02 ± 33.19 |
| **Chrysene\ $C_{16}H_{10}$** | nd | nd | nd | nd | nd | nd | 116.64 ± 28.51 | nd | 151.7 ± 34.06 | 144.72 ± 30.68 | 130.81 ± 28.61 | 116.64 ± 25.60 | 104.16 ± 20.38 | 120.84 ± 23.79 |
| **Benzo[a]pyrene\ $C_{20}H_{12}$** | nd | nd | nd | nd | nd | nd | 90.72 ± 18.87 | nd | 90.2 ± 18.04 | 104.76 ± 20.54 | 110.21 ± 22.76 | 130.68 ± 27.14 | 164.64 ± 37.73 | 157.94 ± 41.21 |
| **Total** | 10.3 ±2.58 | 742.1 ±192.06 | 619.5 ±163.81 | 548.9 ±96.77 | 486.3 ±126.34 | 462.6 ±115.13 | 17022.96 ±3265. | 14994.72 ±3894.68 | 13208.15 ±2933. | 3943.08 ±1004.00 | 3435.05 ±765.19 | 3896.64 ±1021.7 | 3656.8 ±808.82 | 2905.46 ±522. |

| | | | | | | | | | | | | | | |
|---|---|---|---|---|---|---|---|---|---|---|---|---|---|---|
| | | | | | | | **44** | | **91** | | | **4** | | **04** |

Non detected compounds are referred to nd.

a. left to right: hydrothermal plumes from SAP-1_S, SAP-2_S, SAP-3_S, SAP-4_S, SAP-5_S, SMAR, EPR, SWIR, S4_S, S7_S, S8_S, S21_S, S35_S. The samples collection and description are shown in Supplementary Table 1; b. μg $l^{-1}$; c.ng $g^{-1}$

## Supplementary Table3 Hydrocarbon biodegradation of hydrothermal plume derived consortia [d]

| *Compounds* | *Control* | | | *SAP-1* | | | *SAP-2* | | | *SAP-3* | | | *SAP-4* | | | *SAP-5* | | |
|---|---|---|---|---|---|---|---|---|---|---|---|---|---|---|---|---|---|---|
| | **20[a]** | **40[b]** | **60[c]** | **20[a]** | **40[b]** | **60[c]** | **20[a]** | **40[b]** | **60[c]** | **20[a]** | **40[b]** | **60[c]** | **20[a]** | **40[b]** | **60[c]** | **20[a]** | **40[b]** | **60[c]** |
| **Total *n*-alkanes** (decane, dodecane, hexadecane, eicosane, tetracosane) | 6.34 ± 1.52 | 10.44 ± 1.27 | 11.32 ± 2.01 | 68.73 ± 9.64 | 92.18 ± 15.81 | 95.03 ± 24.03 | 65.41 ± 14.31 | 83.81 ± 18.83 | 91.70 ± 19.26 | 73.00 ± 18.63 | 95.75 ± 24.55 | 96.14 ± 20.56 | 57.97 ± 13.26 | 86.98 ± 21.59 | 94.41 ± 16.22 | 67.12 ± 14.66 | 81.01 ± 14.39 | 96.48 ± 24.44 |
| **Total PAHs** (phenanthrene, naphthalene,pyrene, fluoranthene, benzo[α]pyrene) | 3.09 ± 0.20 | 6.77 ± 0.66 | 9.19 ± 0.83 | 28.95 ± 1.25 | 49.67 ± 3.54 | 75.47 ± 9.28 | 21.32 ± 2.52 | 41.13 ± 5.51 | 83.16 ± 7.03 | 19.18 ± 1.75 | 55.76 ± 3.85 | 81.28 ± 7.14 | 22.00 ± 2.14 | 42.01 ± 4.71 | 74.62 ± 6.21 | 25.00 ± 2.93 | 49.01 ± 4.95 | 84.10 ± 6.27 |

a-c, Hydrocarbon biodegradation of at day 20$^{th}$, 40$^{th}$, and 60$^{th}$ incubated at 10 ℃ under 20 MPa.

d, The percentage of hydrocarbons removed by biodegradation ( %). The results were normalized to the total extractable matter and removed abiotic losses.

**Total *n*-alkanes contained** decane, dodecane, hexadecane, eicosane and tetracosane( see Supplementary Materials and Methods).

**Total PAHs** contained phenanthrene, naphthalene,pyrene, fluoranthene, benzo[α]pyrene (see Supplementary Materials and Methods).

**Supplementary Table 4 Hydrocarbon biodegradation of chimney derived consortia** [d]

| *Compounds* | *Control* | | | *SAHCS* | | | *EPHCS* | | | *WSIP* | | |
|---|---|---|---|---|---|---|---|---|---|---|---|---|
| | **20[a]** | **40[b]** | **60[c]** | **20[a]** | **40[b]** | **60[c]** | **20[a]** | **40[b]** | **60[c]** | **20[a]** | **40[b]** | **60[c]** |
| **Total *n*-alkanes** | 6.34 ± 1.18 | 10.44 ± 2.80 | 14.33 ± 2.03 | 55.31 ± 12.28 | 78.27 ± 16.24 | 92.35 ± 22.07 | 60. 1 ± 12.99 | 77.01 ± 14.13 | 94.23 ± 22.47 | 66.47 ± 16.17 | 88.61 ± 22.07 | 97.20 ± 17.18 |
| **Total PAHs** | 1.09 ± 0.24 | 3.73 ± 0.79 | 5.19 ± 0.98 | 35.39 ± 5.34 | 51.72 ± 8.58 | 78.14 ± 14.52 | 26.11 ± 3.75 | 43.67 ± 8.77 | 75.91 ± 11.94 | 19.78 ± 2.41 | 46.79 ± 3.39 | 78.01 ± 8.94 |

a-c, Hydrocarbon biodegradation of at day 20th, 40th, and 60th incubated at 10 ℃ under 30 MPa.

d, The percentage of hydrocarbons removed by biodegradation ( %). The results were normalized to the total extractable matter and removed abiotic losses.

**Total *n*-alkanes contained** decane, dodecane, hexadecane, eicosane and tetracosane(see Supplementary Materials and Methods).

**Total PAHs** contained phenanthrene, naphthalene,pyrene, fluoranthene, benzo[α]pyrene (see Supplementary Materials and Methods).

Supplementary Table 5 Hydrocarbon biodegradation of hydrothermal sediment derived consortia [d]

| ***Compounds( Formula)*** | ***Control*** | | | ***S8*** | | | ***S7*** | | | ***S21*** | | | ***S4*** | | | ***S35*** | | |
|---|---|---|---|---|---|---|---|---|---|---|---|---|---|---|---|---|---|---|
| | **30[a]** | **60[b]** | **90[c]** | **30[a]** | **60[b]** | **90[c]** | **30[a]** | **60[b]** | **90[c]** | **30[a]** | **60[b]** | **90[c]** | **30[a]** | **60[b]** | **90[c]** | **30[a]** | **60[b]** | **90[c]** |
| **Naphtalene ($C_{10}H_8$)** | 17.53 ± 4.37 | 47.4 ± 11.25 | 65.8 ± 14.69 | 41.79 ± 10.63 | 97.27 ± 18.96 | 100 ± 20.47 | 38.15 ± 6.63 | 96.76 ± 18.30 | 100 ± 25.52 | 43.65 ± 9.74 | 97.40 ± 19.77 | 100 ± 25.69 | 38.94 ± 8.58 | 98.73 ± 25.08 | 99.40 ± 17.06 | 37.56 ± 9.96 | 94.73 ± 21.30 | 98.76 ± 17.69 |
| **Phenanthrene ($C_{14}H_{10}$)** | 1.52 ± 0.30 | 5.03 ± 0.88 | 9.25 ± 1.61 | 21.49 ± 3.67 | 66.72 ± 15.11 | 91.63 ± 17.89 | 19.86 ± 3.61 | 60.82 ± 12.83 | 89.04 ± 22.39 | 21.43 ± 4.84 | 58.43 ± 10.15 | 87.98 ± 21.02 | 24.37 ± 5.24 | 66.14 ± 13.27 | 90.34 ± 18.04 | 27.52 ± 4.94 | 72.46 ± 16.69 | 90.46 ± 20.30 |
| **Fluoranthene($C_{16}H_{10}$)** | 0.75 ± 0.19 | 3.77 ± 0.85 | 6.18 ± 1.29 | 17.33 ± 4.30 | 60.98 ± 14.78 | 89.16 ± 18.58 | 18.97 ± 4.18 | 62.34 ± 10.95 | 87.68 ± 23.40 | 16.75 ± 4.23 | 55.37 ± 12.33 | 86.64 ± 18.40 | 14.50 ± 3.18 | 48.73 ± 11.52 | 81.71 ± 17.53 | 15.64 ± 3.51 | 58.08 ± 11.41 | 82.72 ± 19.69 |
| **Pyrene($C_{16}H_{10}$)** | 0.21 ± 0.04 | 2.54 ± 0.53 | 5.08 ± 1.01 | 12.84 ± 2.79 | 58.65 ± 14.63 | 90.26 ± 16.03 | 14.70 ± 3.52 | 53.67 ± 13.37 | 93.46 ± 23.11 | 27.16 ± 5.08 | 71.96 ± 13.92 | 94.34 ± 20.52 | 17.93 ± 4.48 | 52.82 ± 13.58 | 84.77 ± 15.05 | 11.34 ± 2.71 | 53.75 ± 12.60 | 79.78 ± 18.01 |
| **Benzo[a]pyrene($C_{20}H_{12}$)** | 0.18 ± 0.04 | 0.85 ± 0.17 | 1.79 ± 0.40 | 11.56 ± 2.97 | 22.14 ± 5.83 | 45.07 ± 7.78 | 13.27 ± 2.51 | 27.61 ± 6.33 | 44.34 ± 10.27 | 14.33 ± 2.88 | 32.16 ± 6.27 | 50.20 ± 11.02 | 8.94 ± 1.84 | 27.63 ± 5.73 | 46.51 ± 8.83 | 7.46 ± 1.57 | 11.23 ± 1.91 | 18.14 ± 4.38 |

a-c, Hydrocarbon biodegradation of at day 20th, 40th, and 60th incubated at 10 ℃ under 30 MPa.

d, The percentage of hydrocarbons removed by biodegradation ( %). The results were normalized to the total extractable matter and removed abiotic losses.

**Total *n*-alkanes contained** decane, dodecane, hexadecane, eicosane and tetracosane(see Supplementary Materials and Methods).

**Total PAHs** contained phenanthrene, naphthalene,pyrene, fluoranthene, benzo[α]pyrene (see Supplementary Materials and Methods).

**Supplementary Table6 Bacteria isolated from different deep-sea hydrothermal fields**

| *Isolates* | *16SrRNA Acc. No.* | *Closest type strains* | *16S rRNA Identity (%)* | *sample* | *Hydrocarbons* [a] |
|---|---|---|---|---|---|
| 10F6 | KT581465 | *Acinetobacter venetianus* RAG-1[T] | 99.89 | SAP-4 | ++++ |
| 6F9 | KT581460 | *Aestuariibacter halophilus* JC2043[T] | 98.889 | SAP-3 | - |
| S19-9 | KT581525 | *Alcanivorax dieselolei* B-5[T] | 99.545 | EPHCS | ++++ |
| S8-t8 | KT581515 | *Alcanivorax dieselolei* B-5[T] | 99.531 | S8 | ++++ |
| 22F10 | KT581472 | *Alcanivorax hongdengensis* A-11-3[T] | 97.294 | SAP-1 | ++++ |
| YLF38 | KT581573 | *Alcanivorax hongdengensis* A-11-3[T] | 96.935 | SAP-1 | ++++ |
| S13-9 | KT581518 | *Alcanivorax jadensis* T9[T] | 98.862 | EPHCS | ++++ |
| TG4-4 | KT581559 | *Alcanivorax jadensis* T9[T] | 98.98 | SAP-2 | NT |
| S19-12 | KT581527 | *Alcanivorax venustensis* ISO4[T] | 98.182 | EPHCS | ++++ |
| S19-13 | KT581528 | *Alcanivorax venustensis* ISO4[T] | 97.045 | EPHCS | ++++ |
| RY-9 | KT581486 | *Alcanivorax venustensis* ISO4[T] | 99.518 | WSIP | ++++ |
| S35-N3 | KT581551 | *Alcanivorax venustensis* ISO4[T] | 99.411 | S35 | NT |
| s21-H2 | KT581539 | *Alcanivorax venustensis* ISO4[T] | 98.595 | S21 | NT |
| TG4-1 | KT581557 | *Alcanivorax venustensis* ISO4[T] | 100 | SAHCS | NT |
| S19-17 | KT581531 | *Alteromonas macleodii* DSM 6062[T] | 99.659 | EPHCS | ++++ |
| 22F26 | KT581474 | *Alteromonas marina* SW-47[T] | 99.481 | SAP-1 | - |
| 22F32 | KT581476 | *Arenibacter troitsensis* KMM 3674[T] | 99.881 | SAP-1 | NT |
| S35-t28 | KT581553 | *Bacillus firmus* NCIMB 9366[T] | 99.799 | S35 | NT |
| S7- L4 | KT581500 | *Bacillus firmus* NCIMB 9366[T] | 99.799 | S7 | NT |
| TG2-3 | KT581555 | *Bacillus licheniformis* ATCC 14580[T] | 100 | SAHCS | NT |
| S19-10 | KT581526 | *Bacillus pumilus* ATCC 7061[T] | 100 | EPHCS | NT |
| S7 -L9 | KT581502 | *Bacillus pumilus* ATCC 7061[T] | 98.841 | S7 | NT |
| s35-2 | KT581544 | *Bacillus safensis* FO-036b[T] | 99.539 | S35 | ++ |
| s21 –L2 | KT581541 | *Bacillus safensis* FO-036b[T] | 99.731 | S21 | ++ |
| S8 -L9 | KT581514 | *Bacillus safensis* FO-036b[T] | 98.556 | S8 | ++ |
| S4-N3 | KT581495 | *Chromohalobacter salexigens* DSM 3043[T] | 99.911 | S4 | NT |
| RY-3 | KT581480 | *Citreicella marina* CK-I3-6[T] | 100 | WSIP | + |
| 3F6B | KT581458 | *Citreicella thiooxidans* CHLG 1[T] | 99.67 | SAP-4 | - |
| 10F28 | KT581469 | *Croceicoccus marinus* E4A9[T] | 100 | SAP-4 | - |
| S19-6 | KT581524 | *Croceicoccus marinus* E4A9[T] | 98.409 | EPHCS | ++ |
| YLF16 | KT581568 | *Cyclobacterium marinum* LMG 13164[T] | 100 | SAP-1 | - |
| 10F13 | KT581467 | *Dietzia maris* DSM 43672[T] | 99.892 | SAP-4 | +++ |

| | | | | | |
|---|---|---|---|---|---|
| TG8-H1 | KT581563 | *Dietzia maris* DSM 43672$^{(T)}$ | 100 | SAHCS | ++ |
| S13-6 | KT581516 | *Erythrobacter citreus* RE35F/1$^{(T)}$ | 99.545 | EPHCS | - |
| S19-20 | KT581533 | *Erythrobacter citreus* RE35F/1$^{(T)}$ | 99.545 | EPHCS | - |
| TG8-2 | KT581560 | *Erythrobacter citreus* RE35F/1$^{(T)}$ | 99.55 | SAHCS | +++ |
| YLF25 | KT581569 | *Erythrobacter flavus* SW-46$^{(T)}$ | 99.885 | SAP-1 | +++ |
| S35-N8 | KT581552 | *Erythrobacter gangjinensis* K7-2$^{(T)}$ | 97.12 | S35 | +++ |
| S21-N3 | KT581543 | *Erythrobacter gangjinensis* K7-2$^{(T)}$ | 97.761 | S21 | +++ |
| TG8-7 | KT596769 | *Erythrobacter pelagi* UST081027-248$^{(T)}$ | 99.62 | SAHCS | ++ |
| S7-H3 | KT581497 | *Erythrobacter vulgaris* 022 2-10$^{(T)}$ | 99.699 | S7 | NT |
| S8- H4 | KT581511 | *Halomonas axialensis* Althf1$^{(T)}$ | 99.368 | S8 | NT |
| S21-L1 | KT581540 | *Halomonas boliviensis* LC1$^{(T)}$ | 99.369 | S21 | +++ |
| S4-H3 | KT581490 | *Halomonas janggokensis* M24$^{(T)}$ | 99.377 | S4 | NT |
| S13-15 | KT581521 | *Halomonas meridiana* DSM 5425$^{(T)}$ | 100 | EPHCS | - |
| s35 -L1 | KT581547 | *Halomonas meridiana* DSM 5425$^{(T)}$ | 100 | S35 | NT |
| s4-N2 | KT581494 | *Halomonas meridiana* DSM 5425$^{(T)}$ | 99.55 | S4 | NT |
| TG4-2 | KT581558 | *Halomonas meridiana* DSM 5425$^{(T)}$ | 100 | SAHCS | NT |
| RY-7 | KT581484 | *Halomonas titanicae* BH1$^{(T)}$ | 100 | WSIP | +++ |
| S13-8 | KT581517 | *Hyphomonas jannaschiana* ATCC 33883$^{(T)}$ | 100 | EPHCS | - |
| 22F36 | KT581477 | *Hyphomonas jannaschiana* ATCC 33883$^{(T)}$ | 98.925 | SAP-1 | - |
| RY-5 | KT581482 | *Idiomarina abyssalis* KMM 227$^{(T)}$ | 99.767 | WSIP | - |
| S19-4 | KT581523 | *Idiomarina baltica* OS145$^{(T)}$ | 99.615 | EPHCS | - |
| 10F61 | KT581470 | *Idiomarina fontislapidosi* F23$^{(T)}$ | 100 | SAP-5 | - |
| RY-6 | KT581483 | *Idiomarina loihiensis* L2TR$^{(T)}$ | 100 | WSIP | - |
| S7-N2 | KT596766 | *Janibacter terrae* CS12$^{(T)}$ | 99.8 | S7 | |
| 10F16 | KT581468 | *Leeuwenhoekiella palythoae* KMM 6264$^{(T)}$ | 99.646 | SAP-4 | - |
| S35-H3 | KT581546 | *Marinobacter dokdonensis* DSW-8$^{(T)}$ | 99.642 | S35 | NT |
| s21-L3 | KT581542 | *Marinobacter dokdonensis* DSW-8$^{(T)}$ | 97.42 | S21 | NT |
| S4-L1 | KT581491 | *Marinobacter dokdonensis* DSW-8$^{(T)}$ | 99.548 | S4 | NT |
| YLF36 | KT581572 | *Marinobacter algicola* DG893$^{(T)}$ | 99.644 | SAP-1 | ++++ |
| TG8-3 | KT596768 | *Marinobacter bryozoorum* 50-11$^{(T)}$ | 98.86 | SAHCS | ++ |
| S13-11 | KT581519 | *Marinobacter guineae* M3B$^{(T)}$ | 99.091 | EPHCS | - |
| S19-121 | KT581535 | *Marinobacter guineae* M3B$^{(T)}$ | 99.091 | EPHCS | - |
| TG13-2 | KT581564 | *Marinobacter hydrocarbonoclasticus* ATCC 49840$^{(T)}$ | 99.76 | SAHCS | ++++ |
| S19-1 | KT581522 | *Marinobacter hydrocarbonoclasticu* ATCC 49840$^{(T)}$ | 99.545 | EPHCS | ++++ |
| S19-131 | KT581536 | *Marinobacter segnicrescens* SS011B1-4$^{(T)}$ | 99.114 | EPHCS | +++ |
| S19-101 | KT581534 | *Marinobacter vinifirmus* FB1$^{(T)}$ | 99.659 | EPHCS | ++++ |

| | | | | | |
|---|---|---|---|---|---|
| RY-2 | KT581479 | *Marinobacter vinifirmus* FB1(T) | 99.65 | WSIP | ++ |
| S35-L4 | KT581549 | *Marinobacter vinifirmus* FB1(T) | 99.688 | S35 | NT |
| TG2-1 | KT581554 | *Marinobacter vinifirmus* FB1(T) | 99.55 | SAHCS | NT |
| 10F161 | KT581471 | *Martelella mediterranea* MACL11(T) | 99.893 | SAP-5 | - |
| 6F29 | KT581461 | *Mesonia mobilis* KMM 6059(T) | 99.287 | SAP-3 | - |
| S7-N7 | KT581506 | *Microbacterium azadirachtae* AI-S262(T) | 99.144 | S7 | NT |
| S7-N4 | KT581505 | *Microbacterium ginsengiterrae* DCY37(T) | 99.071 | S7 | NT |
| S7-L2 | KT581498 | *Microbacterium resistens* DMMZ 1710(T) | 99.196 | S7 | NT |
| S7-N2-2 | KT581504 | *Micrococcus flavus* LW4(T) | 97.788 | S7 | NT |
| YLF33 | KT581571 | *Muricauda aquimarina* SW-63(T) | 98.565 | SAP-1 | - |
| S7-N1 | KT581503 | *Muricauda aquimarina* SW-63(T) | 99.087 | S7 | NT |
| S7-H2 | KT581496 | *Muricauda beolgyonensis* BB-My12(T) | 99.365 | S7 | NT |
| S8-H5 | KT581512 | *Novosphingobium indicum* H25(T) | 99.191 | S8 | NT |
| 1F8 | KT581454 | *Novosphingobium panipatense* SM16(T) | 97.851 | SAP-2 | - |
| s4 -1 | KT581487 | *Oceanibaculum indicum* P24(T) | 97.941 | S4 | NT |
| s7-N11 | KT581508 | *Oceanibaculum indicum* P24(T) | 99.353 | S7 | NT |
| 22F16 | KT581473 | *Oceanicola marinus* AZO-C(T) | 96.667 | SAP-1 | ++++ |
| 1F26 | KT581457 | *Oceanicola nanhaiensis* SS011B1-20(T) | 100 | SAP-2 | ++++ |
| S19-19 | KT581532 | *Paracoccus stylophorae* KTW-16(T) | 97.725 | EPHCS | - |
| TG8-10 | KT581562 | *Pseudoalteromonas arabiensis* k53(T) | 100 | SAHCS | NT |
| S19-16 | KT581530 | *Pseudoalteromonas lipolytica* LMEB 39(T) | 98.409 | EPHCS | - |
| YLF10 | KT581567 | *Pseudoalteromonas marina* Mano4(T) | 99.887 | SAP-1 | - |
| S4-L2 | KT581492 | *Pseudomonas balearica* SP1402(T) | 98.663 | S4 | NT |
| RY-4 | KT581481 | *Pseudomonas bauzanensis* BZ93(T) | 99.417 | WSIP | +++ |
| S21-N1 | KT596767 | *Pseudomonas indoloxydans* IPL-1(T) | 98.655 | S21 | NT |
| 9F16 | KT596765 | *Pseudomonas pachastrellae* KMM 330(T) | 99.526 | SAP-5 | +++ |
| S8-H2 | KT581510 | *Pseudomonas pachastrellae* KMM 330(T) | 98.41 | S8 | NT |
| S4-L3 | KT581493 | KMM 330(T) | 99.117 | S4 | NT |
| S19-14 | KT581529 | *Pseudomonas stutzeri* ATCC 17588(T) | 99.355 | EPHCS | NT |
| S35-L2 | KT581548 | *Pseudomonas stutzeri* ATCC 17588(T) | 99.805 | S35 | NT |
| S4-a4 | KT581488 | *Pseudomonas stutzeri* ATCC 17588(T) | 98.3 | S4 | NT |
| S7-L5 | KT581501 | *Pseudomonas stutzeri* ATCC 17588(T) | 98.607 | S7 | NT |
| 1F6A | KT581453 | *Pseudoruegeria aquimaris* SW-255(T) | 97.595 | SAP-2 | - |
| S7-N8 | KT581507 | *Pusillimonas noertemannii* BN9(T) | 96.704 | S7 | +++ |
| 1F6 | KT581452 | *Rhodococcus yunnanensis* YIM 70056(T) | 100 | SAP-5 | +++ |
| YLF8 | KT581566 | *Rhodococcus yunnanensis* YYIM 70056(T) | 100 | SAP-1 | ++++ |
| S35-L41 | KT581550 | *Roseovarius pacificus* 2-81(T) | 98.897 | S35 | NT |
| 22F28 | KT581475 | *Ruegeria mobilis* NBRC 101030(T) | 97.66 | SAP-1 | - |
| 9F28 | KT581463 | *Ruegeria mobilis* NBRC 101030(T) | 100 | SAP-5 | - |
| 3F28 | KT581459 | *Sagittula stellata* E-37(T) | 99.886 | SAP-4 | - |

| | | | | | |
|---|---|---|---|---|---|
| RY-8 | KT581485 | *Salegentibacter mishustinae* KMM 6049$^{(T)}$ | 99.176 | WSIP | + |
| RY-1 | KT581478 | *Salinicola salarius* M27$^{(T)}$ | 97.316 | WSIP | ++ |
| TG2-31 | KT581556 | *Salinicola salariu* M27$^{(T)}$ | 99.55 | SAHCS | NT |
| 9F61 | KT581464 | *Shimia marina* CL-TA03$^{(T)}$ | 96.559 | SAP-5 | - |
| S8-L1 | KT581513 | *Sphingobium xenophagum* BN6$^{(T)}$ | 98.482 | S8 | NT |
| YLF28 | KT581570 | *Spongiibacter tropicus* CL-CB221$^{(T)}$ | 99.887 | SAP-1 | +++ |
| 9F27 | KT581462 | *Stappia indica* B106$^{(T)}$ | 99.891 | SAP-5 | - |
| 1F12 | KT581455 | *Sulfitobacter dubius* KMM 3554$^{(T)}$ | 97.033 | SAP-2 | - |
| YLF6 | KT581565 | *Sulfitobacter pontiacus* DSM 10014$^{(T)}$ | 98.511 | SAP-1 | - |
| 1F18 | KT581456 | *Thalassospira tepidiphila* 1-1B$^{(T)}$ | 99.882 | SAP-2 | - |
| S35-H1 | KT581545 | *Thalassospira xiamenensis* M-5$^{(T)}$ | 99.18 | S35 | ++ |
| S21-2L2 | KT581537 | *Thalassospira xiamenensis* M-5$^{(T)}$ | 99.18 | S21 | NT |
| S7-t6 | KT581509 | *Thalassospira xiamenensis* M-5$^{(T)}$ | 99.089 | S7 | NT |
| S21-H1 | KT581538 | *Thalassospira xianhensis* P-4$^{(T)}$ | 98.831 | S21 | NT |
| S4-H2 | KT581489 | *Thalassospira xianhensis* P-4$^{(T)}$ | 98.831 | S4 | NT |
| S7-L3 | KT581499 | *Thalassospira xianhensis* P-4$^{(T)}$ | 98.921 | S7 | NT |
| 10F7 | KT581466 | *Zunongwangia profunda* SMA-87$^{(T)}$ | 96.86 | SAP-4 | - |
| S13-12 | KT581520 | *Zunongwangia profunda* SMA-87$^{(T)}$ | 99.432 | EPHCS | - |
| TG8-5 | KT581561 | *Zunongwangia profunda* SMA-87$^{(T)}$ | 96.93 | SAHCS | NT |

a. Growth capability with hydrocarbons (octane, decane , dodecane, hexadecane, eicosane tetracosane, phenanthrene, naphthalene, pyrene, fluoranthene, and benzo[α]pyrene, see Supplementary Materials and Methods) in NM medium at high pressure and low temperature conditions. NT not tested, (++++), (+++), (++), (+), and (+/−) indicating the growth capability from strong to weak with crude oil as sole carbon and energy source, measured by optical density at 600 nm, (++++) growth (OD 600 >1) after a 60-day incubation at10 ℃ under 30 MPa; (+++) growth (OD 600 >0.6) after a 60-day incubation at 10 ℃ under 30 MPa ; (++) growth (0.6>OD 600 >0.2) after a 60-day incubation at 10 ℃ under 30 MPa; (+) growth (OD 600 <0.2) after 60-day incubation at 10 ℃ under 30 MPa, (−) no growth.

# Mixtrophic bacteria isolates 16S seqences and OTU representative seqences

## *Sulfurimonas* isolates 16S seqences and OTU representative seqences

> *Sulfurimonas* isolate hwp1

AATGAACGCTGGCGGCGTGCTTAACACATGCAAGTCGAACGGTAACAGATGGTAGCTTGCTACTC
TGCTGACGAGTGGCGCACGGGTGAGTAATATATAGATAATGTGCCCCTTGGACCGGGATAGCCAT
TGGAAACGATGATTAATACCGGATACTCCTTCTTACCATAAGGTAAGTCGGGAAATGGATTCCGC
CAAGGGATCGGTCTATATCCCATCAGCTAGTTGGTAGTGTAAGAGACTACCAAGGCAATGACGGG
TAGCGGGTTTGAGAGGATGATCCGCCACACTGGTACTGAGACACGGACCAGACTCCTACGGGAGG
CAGCAGTGAGGAATATTGCACAATGGGGGAAACCCTGATGCAGCAACGCCGCGTGGAGGATGACG
CATTTCGGTGTGTAAACTCCTTTTATATGTCAAGAAAATGACGGTAGCATATGAATAAGCACCGG
CTAACTCCGTGCCAGCAGCCGCGGTAATACGGAGGGTGCAAGCGTTATTCGGAATCACTGGGCGT
AAAGGACGCGTAGGCGGGTTGGCAAGTCAGATGTGAAATCCTACAGCTTAACTGTAGAACTGCAT
TTGAAACTGCCGACCTAGAGTATGGGAGGGGAGATGGAATTAGTGGTGTAGGGGTAAAATCCGT
AGATATCACTAGGAATACCGAAAGCGAAGGCGATCTCCTGGAACATAACTGACGCTAAGGCGTGA
AAGCGTGGGGAGCAAACAGGATTAGATACCCTGGTAGTCCACGCCCTAAACGATGTTCACTAGTC
GTCGCTCTGCTAGTCAGGGCGGTGATGCACTTAACAGATTAAGTGAACCGCCTGGGGAGTACGGT
CGCAAGATTAAAACTCAAAGGAATAGACGGGGACCCGCACAAGTGGTGGAGCATGTGGTTTAATT
CGAAGATACGCGAAGAACCTTACCTAGCCTTGACATTGAGAGAACCTGCCAGAGATGGCGGGGTG
CCCTTTTGGGAGCTCGAAAACAGGTGCTGCACGGCTGTCGTCAGCTCGTGTCGTGAGATGTTGGG
TTAAGTCCCGCAACGAGCGCAACCCTCGTCCTTAGTTGCTAGCAGGTTAAGCTGAGCACTCTAAG
GAGACTGCCTTCGTAAGGAGGAGGAAGGTGAGGACGACGTCAAGTCATCATGGCCCTTATGGCTA
GGGCTACACACGTGCTACAATGGGGCGTACAGAGTGTTGCGATACCGCGAGGTGGAGCTAATCAC
TTAAAGCGTCTCTCAGTTCGGATTGGAGTCTGCAACTCGACTCCATGAAGCTGGAATCACTAGTA
ATCGTAGATCAGCAATGCTACGGTGAATACGTTCCCGGGTCTTGTACTCACCGCCCGTCACACCA
TGGGAGTTGATTTCACCCGAAATCGGGAAGCCAACCTTCGGGGGGCTACCGCTTACGGTGGAATT
AGCGACTGGGGTGAATGAACGCTGGCGGCGTGCTTAACACATGCAAGTCGAACGGTAACAGATGG
TAGCTTGC

> *Sulfurimonas* isolate hwp2

TCAGAATGAACGCTGGCGGCGTGCTTAACACATGCAAGTCGAACGGTAACAGAAGGAGCTTGCTC
CTTGCTGACGAGTGGCGCACGGGTGAGTAATATATAGGTAATGTGCCCCTTGGACCGGGATAGCC
ACTGGAAACGGTGATTAATACTGGATACTCCTTCTACTCATAAGAGTAGTCGGGAAATGGATTCT
GCCAAGGGATCGGCCTATATCCTATCAGCTAGTTGGTGAGGTAAGAGCTCACCAAGGCGATGACG
GGTAGCGGGTTTGAGAGGATGATCCGCCACACTGGTACTGAGACACGGACCAGACTCCTACGGGA
GGCAGCAGTGAGGAATATTGCACAATGGGGGCAACCCTGATGCAGCAACGCCGCGTGGAGGATGA
CGCATTTCGGTGTGTAAACTCCTTTTATATGTCAAGAAAATGACGGTAGCATATGAATAAGCACC
GGCTAACTCCGTGCCAGCAGCCGCGGTAATACGGAGGGTGCAAGCGTTATTCGGAATCACTGGGC
GTAAAGGACGCGTAGGCGGGGTGGAAAGTCAGGTGTGAAAGCCTACAGCTTAACTGTAGAACTGC

ACTTGAAACTCTCACCCTAGAGTATGGGAGGGGCAGATGGAATTAGTGGTGTAGGGGTAAAATCC
GTAGATATCACTAGGAATACCGAAAGCGAAGGCGATCTGCTGGAACATAACTGACGCTAAGGCGT
GAAAGCGTGGGGAGCAAACAGGATTAGATACCCTGGTAGTCCACGCCCTAAACGATGAACACTAG
TCGTCGGGATGCTAGTCATCTCGGTGATGCACTTAACAGATTAAGTGTTCCGCCTGGGGAGTACG
GTCGCAAGATTAAAACTCAAAGGAATAGACGGGGACCCGCACAAGTGGTGGAGCATGTGGTTTAA
TTCGAAGATACGCGAAGAACCTTACCTAGCCTTGACATTGATAGAACACTTTAGAGATAGAGTGG
TGCCCTTTGGGGAGCTTGAAAACAGGTGCTGCACGGCTGTCGTCAGCTCGTGTCGTGAGATGTTG
GGTTAAGTCCCGCAACGAGCGCAACCCTCGTCCTTAGTTGCTAGCAGTTCGGCTGAGCACTCTAA
GGAGACTGCCTTCGCAAGGAGGAGGAAGGTGAGGACGACGTCAAGTCATCATGGCCCTTATGGCT
AGGGCTACACACGTGCTACAATGGGGCGTACAGAGTGTTGCGATACCGCGAGGTGGAGCTAATCA
CTTAAAGCGTCTCTCAGTTCGGATAGCAGTCTGCAACTCGACTGCTTGAAGCTGGAATCACTAGT
AATCGTAGATCAGCAATGCTACGGTGAATACGTTCCCGGGTCTTGTACTCACCGCCCGTCACACC
ATGGGAGTTGATTTCACCCGAAATCGGGAAGCCAACCTTCGGGGGGCTACCGCTTACGGTGGAAT
TAGCGACTGGGGTG

> *Sulfurimonas* isolate hwp3
AGAGTTTGATCCTGGCTCAGAATGAACGCTGGCGGCGTGCTTAACACATGCAAGTCGAACGGTAA
CAGAGAGAAGCTTGCTTCTCTGCTGATGAGTGGCGCACGGGTGAGTAATATATAGATAATGTGCC
TCGCAGACCGGGATAGCCATTGGAAACGATGATTAATACTGGATACTCCTTTTACTCATAAGAGT
AGTCGGGAAACGGATTCTGCTGCGAGATCGGTCTATATCCCATCAGTTAGTTGGTAAGGTAACGG
CTTACCAAGGCAATGACGGGTAGCGGGTTTGAGAGGATGATCCGCCACACTGGTACTGAGACACG
GACCAGACTCCTACGGGAGGCAGCAGTGAGGAATATTGCACAATGGGGGCAACCCTGATGCAGCA
ACGCCGCGTGGAGGATGACGCATTTCGGTGTGTAAACTCCTTTTATATGTCAAGAAAATGACGGT
AGCATATGAATAAGCACCGGCTAACTCCGTGCCAGCAGCCGCGGTAATACGGAGGGTGCAAGCGT
TATTCGGAATCACTGGGCGTAAAGGACGCGTAGGCGGGTTGGTAAGTCAGATGTGAAATCCTACA
GCTTAACTGTAGAACTGCATTTGAAACTACTAACCTAGAGTATGGGAGGGGCAGATGGAATTAGT
GGTGTAGGGGTAAAATCCGTAGATATCACTAGGAATACCGAAAGCGAAGGCGATCTGCTGGAACA
TAACTGACGCTAAGGCGTGAAAGCGTGGGGAGCAAACAGGATTAGATACCCTGGTAGTCCACGCC
CTAAACGATGAACACTAGTCGTCGGGATGCTAGTCATCTCGGTGATGCACTTAACAGATTAAGTG
TTCCGCCTGGGGAGTACGGTCGCAAGATTAAAACTCAAAGGAATAGACGGGGACCCGCACAAGTG
GTGGAGCATGTGGTTTAATTCGAAGATACGCGAAGAACCTTACCTAGCCTTGACATTGATTGAAC
CTTCCAGAGATGGAGGGGTGCCCTTCGGGGAACATGAAAACAGGTGCTGCACGGCTGTCGTCAGC
TCGTGTCGTGAGATGTTGGGTTAAGTCCCGCAACGAGCGCAACCCTCGTCCTTAGTTGCTAGCAG
TTCGGCTGAGCACTCTAAGGAGACTGCCTTCGCAAGGAGGAGGAAGGTGAGGACGACGTCAAGTC
ATCATGGCCCTTATGGCTAGGGCTACACACGTGCTACAATGGGGCGTACAGAGTGTTGCGATACC
GCGAGGTGGAGCTAATCACTTAAAGCGTCTCTCAGTTCGGATTGGAGTCTGCAACTCGACTCCAT
GAAGCTGGAATCACTAGTAATCGTAGATCAGCAATGCTACGGTGAATACGTTCCCGGGTCTTGTA
CTCACCGCCCGTCACACCATGGGAGTTGATTTCACCCGAAATCGGGAAGCCAACCTTCGGGGGGC
TACCGCTTACGGTGGAATTAGCGACTGGGGTGAAGTCGTAACAAGGTAGCCG

> *Sulfurimonas* isolate hwp4
ATGCAAGTCGAACGGTAACAGGAGGAGCTTGCTCCTTGCTGACGAGTGGCGCACGGGTGAGTAAT
ATATAGTTAATGTGCCCCAAAGACTAGGATAGCCACTGGAAACGGTGATTAATACTGGATAAGCC
TCTAAATCAAAAGATTGCGAGGAAAATGTTTTTTCGCTTTGGGATCAGACTATATCCTATCAGCT

AGTTGGTGAGGTAAGAGCTCACCAAGGCAATGACGGGTAGCGGGTTTGAGAGGATGATCCGCCAC
ACTGGTACTGAGACACGGACCAGACTCCTACGGGAGGCAGCAGTGAGGAATATTGCACAATGGAG
GGAACTCTGATGCAGCAACGCCGCGTGGAGGATGACGCATTTCGGTGTGTAAACTCCTTTTATAT
GTCAAGAAAATGACGGTAGCATATGAATAAGCACCGGCTAACTCCGTGCCAGCAGCCGCGGTAAT
ACGGAGGGTGCAAGCGTTACTCGGAATCACTGGGCGTAAAGGACGCGTAGGCGGTTGGAAAGTC
TGATGTGAAATCCTATGGCTCAACCATAGAACTGCATTGGAAACTTCCAACCTAGAGTATGGGAG
GGGGAGATGGAATTAGTGGTGTAGGGGTAAAATCCGTAGATATCACTAGGAATACCTAAAGCGAA
GGCGATCTCCTGGAACATTACTGACGCTAAGGCGTGAAAGCGTGGGGAGCAAACGGGATTAGAAA
CCCTAGTAGTCCACGCCCTAAACGATGAACACTAGTCGTCGGGATGCTTGTCATCTCGGTGATGC
ACTTAACAGATTAAGTGTTCCGCCTGGGGAGTACGGTCGCAAGATTAAAACTCAAAGGAATAGAC
GGGGACCCGCACAAGTGGTGGAGCATGTGGTTTAATTCGACGATACGCGAAGAACCTTACCTGGC
CTTGACATTGATAGAATACTGTAGAGATACGGTAGTGCCCTTCGGGGAACTAGAAAACAGGTGCT
GCACGGCTGTCGTCAGCTCGTGTCGTGAGATGTTGGGTTAAGTCCCGCAACGAGCGCAACCCTCG
TCCTTAGTTGCCAGCAGTTCGGCTGGGCACTCTAAGGAGACTGCCTTCGTAAGGAGGAGGAAGGT
GAGGACGACGTCAAGTCATCATGGCCCTTACGGCCAGGGCTACACACGTGCTACAATGGGGCGTA
CAGAGAGTTGCGATACCGCGAGGTGGAGCTAATCTCTTAAAGCGTCTCTCAGTTCGGATTGTTCT
CTGCAACTCGAGAGCATGAAGCTGGAATCACTAGTAATCGTAGATCAGCAATGCTACGGTGAATA
CGTTCCCGGGTCTTGTACTCACCGCCCGTCACACCATGGGAGTTGATTTCACCCGAAATCGGAAT
GCCAAACTGGCTACCGC

>OTU958
GTGAGGAATATTGCACAATGGAGGAAACTCTGATGCAGCAACGCCGCGTGGAGGATGACGCATTT
CGGTGTGTAAACTCCTTTTATATGTCAAGAAAATGACGGTAGCATATGAATAAGCACCGGCTAAC
TCCGTGCCAGCAGCCGCGGTAATACGGAGGGTGCAAGCGTTATTCGGAATCACTGGGCGTAAAGG
ACACGTAGGCGGGAAGCCAAGTCTGATGTGAAATCCTATGGCTCAACCATAGAACTGCATTGGAA
ACTGGTTACCTAGAGTATGGGAGGGGGAGATGGAATTAGTGGTGTAGGGGTAAAATCCGTAGATA
TCACTAGGAATACCTAAAGCGAAGGCGATCTCCTGGAACATTACTGACGCTAAGGTGTGAAAGCG
TGGGGAGCAAACGGGATTAGATACCCTAGTAGTCC
>OTU1021
GTGAGGAATATTGCACAATGGAGGAAACTCTGATGCAGCAACGCCGCGTGGAGGATGACGCATTT
CGGTGTGTAAACTCCTTTTATATGTCAAGAAAATGACGGTAGCATATGAATAAGCACCGGCTAAC
TCCGTGCCAGCAGCCGCGGTAATACGGAGGGTGCAAGCGTTATTCGGAATCACTGGGCGTAAAGG
ACACGTAGGCGGGAAGCCAAGTCTGATGTGAAATCCTACAGCTTAACTGTAGAACTGCATTGGAA
ACTGGTTACCTAGAGTATGGGAGGGGGAGATGGAATTAGTGGTGTAGGGGTAAAATCCGTAGATA
TCACTAGGAATACCTAAAGCGAAGGCGATCTCCTGGAACATTACTGACGCTAAGGTGTGAAAGCG
TGGGGAGCAAACGGGATTAGAAACCCCAGTAGTCC
>OTU926
GTGAGGAATATTGCACAATGGGGGGAACCCTGATGCAGCAACGCCGCGTGGAGGATGACGCATTT
CGGTGTGTAAACTCCTTTTATATGTCAAGAAAATGACGGTAGCATATGAATAAGCACCGGCTAAC
TCCGTGCCAGCAGCCGCGGTAATACGGAGGGTGCAAGCGTTACTCGGAATCACTGGGCGTAAAGG
ACGCGTAGGCGGGATGGTAAGTCTGATGTGAAATCCTACAGCTTAACTGTAGAACTGCATTGGAA
ACTACTAACCTAGAGTATGGGAGGGGGAGATGGAATTAGTGGTGTAGGGGTAAAATCCGTAGATA
TCACTAGGAATACCTAAAGCGAAGGCGATCTCCTGGAACAATACTGACGCTAAGGCGTGAAAGCG
TGGGGAGCAAACGGGATTAGAAACCCTAGTAGTCC

>OTU937
GTGAGGAATATTGCACAATGGGGGAAACCCTGATGCAGCAACGCCGCGTGGAGGATGACGCATTT
CGGTGTGTAAACTCCTTTTATATGTCAAGAAAATGACGGTAGCATATGAATAAGCACCGGCTAAC
TCCGTGCCAGCAGCCGCGGTAATACGGAGGGTGCAAGCGTTATTCGGAATCACTGGGCGTAAAGG
ACGCGTAGGCGGGTTGGCAAGTCAGATGTGAAATCCTACAGCTTAACTGTAGAACTGCATTTGAA
ACTGCCGACCTAGAGTATGGGAGGGGGAGATGGAATTAGTGGTGTAGGGGTAAAATCCGTAGATA
TCACTAGGAATACCGAAAGCGAAGGCGATCTCCTGGAACATAACTGACGCTAAGGCGTGAAAGCG
TGGGGAGCAAACAGGATTAGATACCCTAGTAGTCC
>OTU969
GTGAGGAATATTGCACAATGGGGGCAACCCTGATGCAGCAACGCCGCGTGGAGGATGACGCATTT
CGGTGTGTAAACTCCTTTTATATGTCAAGAAAATGACGGTAGCATATGAATAAGCACCGGCTAAC
TCCGTGCCAGCAGCCGCGGTAATACGGAGGGTGCAAGCGTTATTCGGAATCACTGGGCGTAAAGG
ACGCGTAGGCGGGTTGGTAAGTCAGATGTGAAATCCTACAGCTTAACTGTAGAACTGCATTTGAA
ACTACTAACCTAGAGTATGGGAGGGGCAGATGGAATTAGTGGTGTAGGGGTAAAATCCGTAGATA
TCACTAGGAATACCGAAAGCGAAGGCGATCTGCTGGAACATAACTGACGCTAAGGCGTGAAAGCG
TGGGGAGCAAACAGGATTAGAAACCCGAGTAGTCC
>OTU1085
GTGAGGAATATTGCACAATGGAGGAAACTCTGATGCAGCAACGCCGCGTGGAGGATGACGCATTT
CGGTGTGTAAACTCCTTTTATAAGGGAAGAAAATGACGGTACCTTATGAATAAGCACCGGCTAAC
TCCGTGCCAGCAGCCGCGGTAATACGGAGGGTGCAAGCGTTACTCGGAATCACTGGGCGTAAAGG
ACACGTAGGCGGATTGGAAAGTCAGATGTGAAATCCTACAGCTTAACTGTAGAACTGCATTTGAA
ACTTCTAATCTAGAGTATGGGAGGGGGAGATGGAATTAGTGGTGTAGGGGTAAAATCCGTAGATA
TCACTAGGAATACCGAAAGCGAAGGCGATCTCCTGGAACATAACTGACGCTAAGGTGTGAAAGCG
TGGGGAGCAAACGGGATTAGATACCCTAGTAGTCC
>OTU2190
GTGAGGAATATTGCACAATGGAGGAAACTCTGATGCAGCAACGCCGCGTGGAGGATGACGCATTT
CGGTGTGTAAACTCCTTTTATATGTCAAGAAAATGACGGTAGCATATGAATAAGCACCGGCTAAC
TCCGTGCCAGCAGCCGCGGTAATACGGAGGGTGCAAGCGTTACTCGGAATCACTGGGCGTAAAGG
ACGCGTAGGCGGACGGTCAAGTCAGATGTGAAATCCTACAGCTTAACTGTAGAACTGCATTTGAA
ACTAATCGTCTAGAGTATGGGAGGGGGAGATGGAATTAGTGGTGTAGGGGTAAAATCCGTAGATA
TCACTAGGAATACCGAAAGCGAAGGCGATCTCCTGGAACATAACTGACGCTAAGGCGTGAAAGCG
TGGGGAGCAAACAGGATTAGAAACCCTAGTAGTCC

>OTU2220
GTGAGGAATATTGCACAATGGGGGAAACCCTGATGCAGCAACGCCGCGTGGAGGATGACGCATTT
CGGTGTGTAAACTCCTTTTATGAGTCAAGAAAATGACGGTAGCTCATGAATAAGCACCGGCTAAC
TCCGTGCCAGCAGCCGCGGTAATACGGAGGGTGCAAGCGTTACTCGGAATCACTGGGCGTAAAGG
ACGCGTAGGCGGGTTATCAAGTCAGGTGTGAAATCCTATGGCTCAACCATAGAACTGCACTTGAA
ACTGGTAACCTAGAGTATGGGAGGGGCAGATGGAATTAGTAGTGTAGGGGTAAAATCCGTAGATA
TTACTAGGAATACCGAAAGCGAAGGCGATCTGCTGGAACATTACTGACGCTAAGGCGTGAAAGCG
TGGGGAGCAAACGGGATTAGAAACCCTAGTAGTCC

>OTU1193
GTGAGGAATATTGCACAATGGAGGGAACTCTGATGCAGCAACGCCGCGTGGAGGATGACGCATTT

CGGTGTGTAAACTCCTTTTATATGTCAAGAAAATGACGGTAGCATATGAATAAGCACCGGCTAAC
TCCGTGCCAGCAGCCGCGGTAATACGGAGGGTGCAAGCGTTACTCGGAATCACTGGGCGTAAAGG
ACGCGTAGGCGGGTTGGAAAGTCTGATGTGAAATCCTATGGCTCAACCATAGAACTGCATTGGAA
ACTTCCAACCTAGAGTATGGGAGGGGGAGATGGAATTAGTGGTGTAGGGGTAAAATCCGTAGATA
TCACTAGGAATACCTAAAGCGAAGGCGATCTCCTGGAACATTACTGACGCTAAGGCGTGAAAGCG
TGGGGAGCAAACGGGATTAGAAACCCTAGTAGTCC
>OTU1172
GTGAGGAATATTGCACAATGGAGGGAACTCTGATGCAGCAACGCCGCGTGGAGGATGACGCATTT
CGGTGTGTAAACTCCTTTTATATGTCAAGAAAATGACGGTAGCATATGAATAAGCACCGGCTAAC
TCCGTGCCAGCAGCCGCGGTAATACGGAGGGTGCAAGCGTTACTCGGAATCACTGGGCGTAAAGG
ACGCGTAGGCGGGTTGGAAAGTCTGATGTGAAATCCTATGGCTCAACCATAGAACTGCATTGGAA
ACTTCCAACCTAGAGTATGGGAGGGGGAGATGGAATTAGTGGTGTAGGGGTAAAATCCGTAGATA
TCACTAGGAATACCTAAAGCGAAGGCGATCTCCTGGAACATTACTGACGCTAAGGCGTGAAAGCG
TGGGGAGCAAACGGGATTAGATACCCTTGTAGTCC
>OTU1201
GTGAGGAATATTGCACAATGGGGGAAACCCTGATGCAGCAACGCCGCGTGGAGGATGACGCATTT
CGGTGTGTAAACTCCTTTTATATGTCAAGAAAATGACGGTAGCATATGAATAAGCACCGGCTAAC
TCCGTGCCAGCAGCCGCGGTAATACGGAGGGTGCAAGCGTTATTCGGAATCACTGGGCGTAAAGG
ACGCGTAGGCGGGTTGGCAAGTCAGATGTGAAATCCTACAGCTTAACTGTAGAACTGCATTTGAA
ACTGCCGACCTAGAGTATGGGAGGGGGAGATGGAATTAGTGGTGTAGGGGTAAAATCCGTAGATA
TCACTAGGAATACCGAAAGCGAAGGCGATCTCCTGGAACATAACTGACGCTAAGGCGTGAAAGCG
TGGGGAGCAAACAGGATTAGAAACCCTAGTAGTCC
>OTU1212
GTGAGGAATATTGCACAATGGGGGCAACCCTGATGCAGCAACGCCGCGTGGAGGATGACGCATTT
CGGTGTGTAAACTCCTTTTATATGTCAAGAAAATGACGGTAGCATATGAATAAGCACCGGCTAAC
TCCGTGCCAGCAGCCGCGGTAATACGGAGGGTGCAAGCGTTATTCGGAATCACTGGGCGTAAAGG
ACGCGTAGGCGGGGTGGAAAGTCAGGTGTGAAAGCCTACAGCTTAACTGTAGAACTGCACTTGAA
ACTCCCACCCTAGAGTATGGGAGGGGCAGATGGAATTAGTGGTGTAGGGGTAAAATCCGTAGATA
TCACTAGGAATACCGAAAGCGAAGGCGATCTGCTGGAACATAACTGACGCTAAGGCGTGAAAGCG
TGGGGAGCAAACAGGATTAGAAACCCCAGTAGTCC
>OTU1044
GTGAGGAATATTGCACAATGGGGGCAACCCTGATGCAGCAACGCCGCGTGGAGGATGACGCATTT
CGGTGTGTAAACTCCTTTTATATGTCAAGAAAATGACGGTAGCATATGAATAAGCACCGGCTAAC
TCCGTGCCAGCAGCCGCGGTAATACGGAGGGTGCAAGCGTTATTCGGAATCACTGGGCGTAAAGG
ACGCGTAGGCGGGTTGGTAAGTCAGATGTGAAATCCTACAGCTTAACTGTAGAACTGCATTTGAA
ACTACTAACCTAGAGTATGGGAGGGGCAGATGGAATTAGTGGTGTAGGGGTAAAATCCGTAGATA
TCACTAGGAATACCGAAAGCGAAGGCGATCTGCTGGAACATAACTGACGCTAAGGCGTGAAAGCG
TGGGGAGCAAACAGGATTAGAAACCCGTGTAGTCC

**SAR324 isolates 16S seqences and OTU representative seqences**

> SAR324 isolate hwp5

AGTTTGATCCTGGCTCAGGACGAACGCTGGCGGCATGCCTAACACATGCAAGTCGAACGAGAAAG
TTCCTTCGGGAGCAATTAAAGTGGCGCACGGGTGAGTAACGCGTAGACAATCTGCCCTTCAGTCT
GGGACAACTTTTCGAAAGGAGAGCTAATACCGGATAACAATGTTTAACCTAAGTTAAGTATTTGA
AAGCTTTATGTGCTGAAGGAGGGGTCTGCGTCCCATTAGCTAGTTGGTAAGGTAAAGGCTTACCA
AGGCAACGATGGGTAGCGGGTTTGAGAGGACGATCCGCCACACTGGAACTGAGACACGGTCCAGA
CTCCTACGGGAGGCAGCAGTGGGGAATATTGCACAATGGAGGCAACTCTGATGCAGCAATGTCGC
GTGAGTGAAGAAGGCCCTTGGGTCGTAAAGCTCTTTTATGGGGGAAGATGATGACGGTACCCCAA
GAATAAGCACCGGCTAACTATGTGCCAGCAGCCGCGGTAATACATAGGGTGCGAGCGTTGTTCGG
AATTACTGGGCGTAAAGGGCGCGCAGGCGGAATAGTAAGTCGGAGGTGAAAGCCCGGGGCTCAAC
CCCGGAGGGTCTTTCGAAACTGCTAATCTAGAGAGGGTCAGGGGCCGGCAGAATTCCTGGTGTAG
AGGTGAAATTCGTAGATATCAGGAGGAATACCGGTGGCGAAGGCGGCCGGCTGGGGCCACTCTGA
CGCTGAGGCGCGAAAGCGTGGGGAGCAAACAGGATTAGATACCCTGGTAGTCCACGCCGTAAACG
ATGAGCACTGGACGTCCGGAGGGTTCGACCCTTCTGGGTGTCTCAGCTAACGCATTAAGTGCTCC
GCCTGGGGAGTACGGTCGCAAGACTAAAACTCAAAGGAATTGACGGGGGCCCGCACAAGCGGTGG
AACATGTGGTTTAATTCGATGCAACGCGAAGAACCTTACCTGGTCTTGACATCCTCGGACCGCTT
CAGAGATGAAGTTTCCTCTTCGGAGGCCGAGTGACAGGTGCTGCATGGCTGTCGTCAGCTCGTGT
CGTGAGATGTTGGGTTAAGTCCCGCAACGAGCGCAACCCCTACCCTTAATTGCCATCGGGTTATG
CCGGGCACTTTAGGGGGACTGCCGGTGACAAGCCGGAGGAGGGTGGGGATGACGTCAAGTCCTCA
TGGCCTTTATGACCAGGGCTACACACGTGTTACAATGGGAGTTACAGAGGGAAGCTAAACCGCGA
GGTCATGCCAATCCCAGAAAAACTTTCTCAGTTCGGATCGCAGTCTGCAACTCGACTGCGTGAAG
CTGGAATCGCTAGTAATCGCGGATCAGCACGCCGCGGTGAATACGTTCCCGGGCCTTGTACACAC
CGCCCGTCACACCATGGGAGCCGACAAGGGCAGAAGTCGCCGAGCTAACCCAGCACCTTTTTTGG
GTATGGAGCATGAGAATATTATTCA

> SAR324 isolate hwp6

ACGAACGCTGGCGGCATGCCTAACACATGCAAGTCGAACGAGAAAGTCACTTCGGTGGCGATTAA
AGTGGCGCACGGGTGAGTAACGCGTAGACAATCTGCCCTTCAGTCTGGGACAACTTTTCGAAAGG
AGAGCTAATACCGGATAACAATGCATGACACAAGTCATATATTTGAAAGCTTTATGTGCTGAAGG
AGGGGTCTGCGTCCCATTAGCTGGTTGGTGAGGTAAAGGCTCACCAAGGCAACGATGGGTAGCGG
GTTTGAGAGGACGATCCGCCACACTGGAACTGAGACACGGTCCAGACTCCTACGGGAGGCAGCAG
TGGGGAATATTGCACAATGGAGGAAACTCTGATGCAGCAATGCCGCGTGAGTGAAGAAGGCCCTT
GGGTCGTAAAGCTCTTTTATGAGGGAAGATGATGACGGTACCTCAGGAATAAGCACCGGCTAACT
ACGTGCCAGCAGCCGCGGTAATACGTAGGGTGCGAGCGTTGTTCGGAATTACTGGGCGTAAAGGG
CGCGCAGGCGGAATAGCAAGTCGGAGGTGAAAGCCCGGGGCTCAACCCCGGAGGGTCTTTCGAAA
CTGCTAATCTAGAGAGGGTCAGGGGCCGGCAGAATTCCTGGTGTAGAGGTGAAATTCGTAGATAT
CAGGAGGAATACCGGTGGCGAAGGCGGCCGGCTGGGGCCACTCTGACGCTGAGGCGCGAAAGCGT
GGGGAGCAAACAGGATTAGAAACCCCAGTAGTCCACGCCGTAAACGATGAGCACTGGATGTCCGG

AGGGTTCGACCCTTCTGGGTGTCTCAGCTAACGCATTAAGTGCTCCGCCTGGGGAGTACGGTCGC
AAGACTAAAACTCAAAGGAATTGACGGGGGCCCGCACAAGCGGTGGAACATGTGGTTTAATTCGA
TGCAACGCGAAGAACCTTACCTGGTCTTGACATCCTCGGATCGCTTCAGAGATGAAGTTTCCTCT
TCGGAGGCCGAGTGACAGGTGCTGCATGGCTGTCGTCAGCTCGTGTCGTGAGATGTTGGGTTAAG
TCCCGCAACGAGCGCAACCCCTACCCTTAATTGCCATCGGGTTATGCCGGGCACTTTAGGGGGAC
TGCCGGTGACAAGCCGGAGGAGGGTGGGGATGACGTCAAGTCCTCATGGCCTTTATGACCAGGGC
TACACACGTGTTACAATGGGAGTTACAGAGGGAAGCTAAACCGCGAGGTCATGCCAATCCCAGAA
AAACTTTCTCAGTTCGGATCGCAGTCTGCAACTCGACTGCGTGAAGCTGGAATCGCTAGTAATCG
CGGATCAGCACGCCGCGGTGAATACGTTCCCGGGCCTTGTACACACCGCCCGTCACACCATGGGA
GCCGACAAGGGCAGAAGTCGCCGAGCCAACCTTCGGGAAGCAGGCGCCCAAGCTGTTGTTGA

>OTU2178
GTGGGGAATATTGCACAATGGAGGCAACTCTGATGCAGCAATGTCGCGTGAGTGAAGAAGGCCCT
TGGGTCGTAAAGCTCTTTTATGGGGGAAGATGATGACGGTACCCCAAGAATAAGCACCGGCTAAC
TATGTGCCAGCAGCCGCGGTAATACATAGGGTGCGAGCGTTGTTCGGAATTACTGGGCGTAAAGG
GCGCGCAGGCGGAATAGTAAGTCGGAGGTGAAAGCCCGGGGCTCAACCCCGGAGGGTCTTTCGAA
ACTGCTAATCTAGAGAGGGTCAGGGGCCGGCAGAATTCCTGGTGTAGAGGTGAAATTCGTAGATA
TCAGGAGGAATACCGGTGGCGAAGGCGGCCGGCTGGGGCCACTCTGACGCTGAGGCGCGAAAGCG
TGGGGAGCAAACAGGATTAGAAACCCTAGTAGTCC
>OTU2942
GTGGGGAATATTGCACAATGGAGGAAACTCTGATGCAGCAATGCCGCGTGAGTGAAGAAGGCCCT
TGGGTCGTAAAGCTCTTTTATGAGGGAAGATGATGACGGTACCTCAGGAATAAGCACCGGCTAAC
TACGTGCCAGCAGCCGCGGTAATACGTAGGGTGCGAGCGTTGTTCGGAATTACTGGGCGTAAAGG
GCGCGCAGGCGGAATAGCAAGTCGGAGGTGAAAGCCCGGGGCTCAACCCCGGAGGGTCTTTCGAA
ACTGCTAATCTAGAGAGGGTCAGGGGCCGGCAGAATTCCTGGTGTAGAGGTGAAATTCGTAGATA
TCAGGAGGAATACCGGTGGCGAAGGCGGCCGGCTGGGGCCACTCTGACGCTGAGGCGCGAAAGCG
TGGGGAGCAAACAGGATTAGAAACCCCAGTAGTCC
>OTU1989
GTAGGGAATATTGCGCAATGGGGGCAACCCTGACGCAGCAATGCCGCGTGAGTGAAGAAGGCCTT
CGGGTCGTAAAGCTCTTTTATGGGGGAAGATGATGACGGTACCCCATGAATAAGCACCGGCTAAC
TACGTGCCAGCAGCCGCGGTAATACGTAGGGTGCGAGCGTTGTTCGGAATTACTGGGCGTAAAGG
GCGTGCAGGCGGATTGGCAAGCCGGAGGTGAAAGCCCGGGGCTCAACCCCGGAGGGTCTTTCGGA
ACTGCCAGTCTTGAGAGGGTCAGGGGCCAGCGGAATTCCTGGTGTAGAGGTGAAATTCGTAGAGA
TCAGGAGGAACACCGGCGGCGAAAGCGGCTGGCTGGGGCCACTCTGACGCTGAGGCGCGAAAGCG
TGGGGAGCAAACAGGATTAGATACCCGAGTAGTCC
>OTU2181
GTGGGGAATCTTGCGCAATGGGCGCAAGCCTGACGCAGCGATGCCGCGTGGGTGAAGAAGGCCTT
CGGGTCGTAAAGCCCTTTTGGTGGGGAGGATGATGACGTCACCCACCGAATAAGCGCCGGCTAAC
TACGTGCCAGCAGCCGCGGTAATACGTAGGGCGCGAGCGTTGTCCGGAATCACTGGGCGTAAAGG
GCGCGCAGGTGGGTTCGCAAGCGGGGGGTGAAAGGCCGGGGCTCAACCCCGGGACTGCTTCCCGA
ACTGCGGATCTAGAGGGGGTCAGGGGCCAGCGGAATTCCGGGTGGAGAGGTGAAATTCGTAGAGA
TCCGGAGGAACGCCGGCGGCGAAAGCGGCTGGCTGGGGCCGCCCTGACACTGAGGCGCGAGAGCG
TGGGGAGCAAACAGGATTAGAAACCCTTGTAGTCC
>OTU992

GTGGGGAATATTGCGCAATGGGCGAAAGCCTGACGCAGCAATGCCGCGTGAGTGAAGAAGGCTTT
CGGGTCGTAAAGCTCTTTATCTCTGTGAAGATAATGACGGTAGCAGAGGAATAAGCACCGGCTAA
CTACGTGCCAGCAGCCGCGGTAATACGTAGGGTGCGAGCGTTGTTCGGAATTACTGGGCGTAAAG
GGTGCGTAGGCGGAACGGTCAGCTAGAGGTGAAAGCCCGGGGCTCAACCCCGGAATTGCTTCTGG
AACTGCCGATCTAGAGAGGGTCAGAGGTCAGCGGAATTCCTAGTGTAGAGGTGAAATTCGTAGAT
ATTAGGAGGAACACCGGCGGCGAAAGCGGCTGGCTGGGGCCACTCTGACGCTGAGGCACGAAAGC
GTGGGGAGCAAACGGGATTAGAAACCCCAGTAGTCC
>OTU2658
GTGGGGAATCTTGCGCAATGGGCGAAAGCCTGACGCAGCAATGCCGCGTGAGTGAAGAAGGCCTT
AGGGTCGTAAAGCTCTTTATCTCTACGAAGATGATGACGGTAGTAGAGGAATAAGCACCGGCTAA
CTACGTGCCAGCAGCCGCGGTAATACGTAGGGTGCGAGCGTTGTTCGGAATTACTGGGCGTAAAG
GGTGTGTAGGCGGAATGGTCAGCTAGAGGTGAAAGCCCGGGGCTCAACCCCGGAACGGCTTTTAG
AACTGCCATTCTAGAGAGGGTCAGAGGCTAGCGGAATTCCTGGTGTAGAGGTGAAATTCGTAGAT
ATCAGGAGGAACACCGGCGGCGTAAGCGGCTGGCTGGGGCCACTCTGACGCTGAGGCACGAAAGC
GTGGGGAGCAAACAGGATTAGAAACCCCGGTAGTCC
>OTU1347
GTGGGGAATCTTGCGCAATGGGCGAAAGCCTGACGCAGCAATGCCACGTGAGTGAAGAAGGCTTT
CGGGTCGTAAAGCTCTTTATCTCAGGGAAGATAATGACGGTACCTGAGGAATAAGCACCGGCTAA
CTACGTGCCAGCAGCCGCGGTAATACGTAGGGTGCGAGCGTTGTTCGGAATTACTGGGCGTAAAG
GGTGTGTAGGCTGACTAGTCAGCCAGGGGTGAAAGCCCGGAGCTCAACTCCGGAACTGCTTCTGG
AACTGCTAGTCTAGAGAGGGTCAGAGGCCAACGGAATTCCTGGTGTAGAGGTGAAATTCGTAGAT
ATCAGGAGGAACACCGGTGGCGAAAGCGGTTGGCTAGGGCCACTCTGACGCTGAGGCACGAAAGC
GTGGGGAGCAAACAGGATTAGAAACCCTAGTAGTCC
>OTU1187
GTGGGGAATCTTGCGCAATGGACGAAAGTCTGACGCAGCGATGCCGCGTGAGTGAAGAAGGTTTT
CGGATCGTAAAGCTCTTTTCTGAGGGAAGATGATGACGGTACCTCAGGAATAAGCACCGGCTAAC
TACGTGCCAGCAGCCGCGGTAATACGTAGGGTGCGAGCGTTGTTCGGATTTACTGGGCGTAAAGG
GTGCGTAGGCGGACCCATAAGCTAGAGGTGAAAGCCCGGGGCTCAACCCCGGAGGCGCTTTTAGA
ACTGTGGGTCTAGAGAGGGTCAGAGGCTAGCGGAATTCCTGGTGTAGAGGTGAAATTCGTAGATA
TCAGGAGGAACACCGGCGGCGAAAGCGGCTGGCTGGGGCCACTCTGACGCTGAGGCACGAAAGCG
TGGGGAGCGAACGGGATTAGATACCCCAGTAGTCC
>OTU1368
GTGGGGAATCTTGCGCAATGGGCGAAAGCCTGACGCAGCAATGCCGCGTGAGTGAAGAAGGTTTT
CGGATCGTAAAGCTCTTTATCTGAGGGAAGATGATGACGGTACCTCAGGAATAAGCACCGGCTAA
CTACGTGCCAGCAGCCGCGGTAATACGTAGGGTGCGAGCGTTGTTCGGAATTACTGGGCGTAAAG
GGTGCGCAGGCGGAAGGACAAGCTAGGGGTGAAAGCCCGGGGCTCAACCCCGGAGGTGCTCTTAG
AACTGTTCATCTGGAGAGGGTCAGAGGCTAGCGGAATTCCGGGTGTAGAGGTGAAATTCGTAGAT
ATCCGGAGGAACACCGGCGGCGAAAGCGGCTGGCTGGGGCCACTCTGACGCTGAGGCACGAAAGC
GTGGGGAGCAAACGGGATTAGAAACCCTTGTAGTCC
>OTU1767
GTGGGGAATCTTGCGCAATGGGCGAAAGCCTGACGCAGCAATGCCGCGTGAGTGAAGAAGGCCTT
CGGGTCGTAAAGCTCTTTTCTCAGGGAAGATGATGACGGTACCTGAGGAATAAGCACCGGCTAAC
TACGTGCCAGCAGCCGCGGTAATACGTAGGGTGCAAGCGTTGTTCGGAATCACTGGGCGTAAAGG
GCGTGTAGGCGGAATAGTAAGCTAGAGGTGAAAGCCCGGGGCTCAACCCCGGAACTGCTTCTGGA

ACTGCTAATCTAGAGAGGGTCAGAGGTCAGCGGAATTCCTGGTGTAGAGGTGAAATTCGTAGATA
TCAGGAGGAACACCGGTGGCGAAAGCGTCTGACTGGGGCCACTCTGACGCTGAGGCGCGAAAGCG
TGGGGAGCAAACAGGATTAGATACCCGGGTAGTCC

**SUP05 isolates 16S seqences and OTU representative seqences**

> SUP05 isolate hwp7
GAGTTTGATCCTGGCTCAGATTGAACGCTGGCGGTATGCTTAACACATGCAAGTCGAACGGAAAC
GATGGTAGCTTGCTACCAGGCGTCGAGTGGCGGACGGGTGAGTAACGCGTAGGAATCTACCTGAT
AGTGGGGGATAGCCCAGAGAAATCTGGATTAATACCGCATAATCTCTATGGAGTAAAGGAGCCCT
CTTCTTGAAAGGTTTCGCTATCAGATGAGCCTGCGTAAGATTAGCTTGTTGGTGAGGTAAAAGCT
CACCAAGGCGACGATCTTTAGCTGGTCTGAGAGGATGATCAGCCACACTGGAACTGAGACACGGT
CCAGACTCCTACGGGAGGCAGCAGTGGGGAATATTGGACAATGGGCGCAAGCCTGATCCAGCAAT
ACCGCGTGTGTGAAGAAGGCCTGAGGGTTGTAAAGCACTTTCAATTGTGAAGAAAAGCTAATGGC
TAATACCCATTAGCCTTGACGTTAACTTTAGAAGAAGCACCGGCTAACTCCGTGCCAGCAGCCGC
GGTAATACGGAGGGTGCAAGCGTTAATCGGAATTACTGGGCGTAAAGCGTGCGTAGGTGGTTTGT
TAAGTTAGATGTGAAAGCCCTGGGCTCAACCTAGGAACTGCATTTAAAACTGGCAAACTAGAGTA
TAGGAGAGGAAAGTGGAATTTCAGGTGTAGCGGTGAAATGCGTAGATATCTGAAGGAACATCAAT
GGCGAAGGCAGCTTTCTGGACTAATACTGACACTGAGGTACGAAAGCGTGGGTAGCAAACAGGAT
TAGATACCCTGGTAGTCCACGCCGTAAACGATGGCAACTAGCCGTTGGGAGGATTTACCTCTTAG
TGGCGAAGCTAACGCGTTAAGTTGTCCGCCTGGGGAGTACGGCCGCAAGGTTAAAACTCAAAGGA
ATTGACGGGGACCCGCACAAGCGGTGGAGCATGTGGTTTAATTCGATGCAACGCGAAGAACCTTA
CCTGGTCTTGACATACACGGAACTTTCCAGAGATGGATTGGTGCCTTCGGGAACCGTGATACAGG
TGCTGCATGGCTGTCGTCAGCTCGTGTCGTGAGATGTTGGGTTAAGTCCCGTAACGAGCGCAACC
CTTATCCTTATTTGCCAGCACATCATGGTGGGAACTATAAGGAGACTGCCGGTGATAAACCGGAG
GAAGGCGGGGACGACGTCAAGTCATCATGGCCCTTACGACCAGGGCTACACACGTGCTACAATGG
GAAGGACAAAGGGTTGCTAAGCCGCGAGGTGGTGCTAATCTCATAAACCTTTTCGTAGTCCGGAT
CGGAGTCTGCAACTCGACTCCGTGAAGTCGGAATCGCTAGTAATCGTAGATCAGAACGCTACGGT
GAATACGTTCCCGGGTCTTGTACACACCGCCCGTCATACCATGGGAGTGGGTTGCACCAGAAGTA
GCTAGTCTAACTGTTTACAGAGGACGGTTACCACGGTGTGATTCATGACTGGGGTAAAGTCGTAA
CAAGGTAGC
>OTU244
GTGGGGAATATTGGACAATGGGCGCAAGCCTGATCCAGCAATACCGCGTGTGTGAAGAAGGCCTG
AGGGTTGTAAAGCACTTTCAATTGTGAAGAAAAGTTAATGGTTAATACCCATTAGCCTTGACGTT
AACTTTAGAAGAAGCACCGGCTAACTCCGTGCCAGCAGCCGCGGTAATACGGAGGGTGCAAGCGT
TAATCGGAATTACTGGGCGTAAAGCGTGCGTAGGTGGTTTGTTAAGTCAGATGTGAAAGCCCCGG
GCTCAACCTGGGAACTGCATTTGAAACTGGCATACTAGAGTATAGGAGAGGAAAGTGGAATTTCA
GGTGTAGCGGTGAAATGCGTAGATATCTGAAGGAACATCAATGGCGAAGGCAGCTTTCTGGACTA
ATACTGACACTGAGGTACGAAAGCGTGGGTAGCAAACAGGATTAGAAACCCTAGTAGTCC
>OTU884
GTGGGGAATATTGGACAATGGGCGCAAGCCTGATCCAGCAATACCGCGTGTGTGAAGAAGGCCTG
AGGGTTGTAAAGCACTTTCAATTGTGAAGAAAAGTTTAGCGTTAATACCGCTAAACCTTGACGTT
AACTTTAGAAGAAGCACCGGCTAACTCCGTGCCAGCAGCCGCGGTAATACGGAGGGTGCAAGCGT
TAATCGGAATTACTGGGCGTAAAGCGTGCGTAGGTGGTTTGTTTAGTCAGATGTGAAAGCCCTGG

GCTCAACCTAGGAACTGCATTTGAAACTGGCAAACTAGAGTATAGGAGAGGAAAGTGGAATTTCA
GGTGTAGCGGTGAAATGCGTAGATATCTGAAGGAACATCAATGGCGAAGGCAGCTTTCTGGACTA
ATACTGACACTGAGGTACGAAAGCGTGGGTAGCAAACAGGATTAGAAACCCGAGTAGTCC
>OTU2265
GTGGGGAATATTGGACAATGGGCGCAAGCCTGATCCAGCAATACCGCGTGTGTGAAGAAGGCCTG
AGGGTTGTAAAGCACTTTCAATTGTGAAGAAAAGCTAATGGCTAATACCCATTAGCCTTGACGTT
AACTTTAGAAGAAGCACCGGCTAACTCCGTGCCAGCAGCCGCGGTAATACGGAGGGTGCAAGCGT
TAATCGGAATTACTGGGCGTAAAGCGTGCGTAGGTGGTTTGTTAAGTTAGATGTGAAAGCCCTGG
GCTCAACCTAGGAACTGCATTTAAAACTGGCAAACTAGAGTATAGGAGAGGAAAGTGGAATTTCA
GGTGTAGCGGTGAAATGCGTAGATATCTGAAGGAACATCAATGGCGAAGGCAGCTTTCTGGACTA
ATACTGACACTGAGGTACGAAAGCGTGGGTAGCAAACAGGATTAGATACCCGAGTAGTCC
>OTU2477
GTGGGGAATATTGGACAATGGGCGCAAGCCTGATCCAGCAATACCGCGTGTGTGAAGAAGGCCTG
AGGGTTGTAAAGCACTTTCAATTGTGAAGAAAAGCTAATGGCTAATACCCATTAGCCTTGACGTT
AACTTTAGAAGAAGCACCGGCTAACTCCGTGCCAGCAGCCGCGGTAATACGGAGGGTGCAAGCGT
TAATCGGAATTACTGGGCGTAAAGCGTGCGTAGGTGGTTTGTTAAGTTAGATGTGAAAGCCCTGG
GCTCAACCTAGGAACTGCATTTAAAACTGGCAAACTAGAGTATAGGAGAGGAAAGTGGAATTTCA
GGTGTAGCGGTGAAATGCGTAGATATCTGAAGGAACATCAATGGCGAAGGCAGCTTTCTGGACTA
ATACTGACACTGAGGTACGAAAGCGTGGGTAGCAAACAGGATTAGATACCCTAGTAGTCC

**SAR202 OTU representative seqences**
>OTU118
GCAGGGAATCTTCCACAATGGGCGAAAGCCTGATGGAGCGACGCCGCGTGAGGGATGAAGGCTCT
AGGGTCGTAAACCTCTTTTCTCAGGGAAGAGCAAGGACGGTACCTGAGGAATAAGTGACGGCTAA
CTACGTGCCAGCAGCCGCGGTAATACGTAGGTCACAAACGTTGTCCGGATTTATTGGGCGTAAAG
GGTCCGTAGGCGGTTCGGTAAGTCTCCTGTGAAATCTTCAGGCTCAACTTGAAGAGGTCGGGGGA
TACTGCCGGACTTGAGACTGTTAGAGGCAAGTGGAATTCCCGGTGTAGTGGTGAAATGCGTAGAT
ATCGGGAGGAACACCAGTGGCGAAAGCGGCTTGCTGGGACAGTTCTGACGCTGAGGGACGAAAGC
GTGGGTAGCAAACCGGATTAGAAACCCTAGTAGTCC
>OTU164
GCAGGGAATTTTGGGCAATGGACGAAAGTCTGACCCAGCGACGTCGCGTGGGGGATGAAGGCTCT
AGGGCTGTAAACCCCTTTTCCAAGGGAAGAGGAAGGACGGTACCTTGGGAATAAGCTCCGGCTAA
CTACGTGCCAGCAGCCGCGGTAATACGTAGGGAGCGAGCGTTACCCGGAATTACTGGGCGTAAAG
AGCACGTAGGCGGCTGGATAAGTCTTCTGTGAAAGCTCCCGGCTTAACTGGGAGAGGTCAGGGGA
TACTATTCGGCTTGAGGGCAGTAGAGGTAAGCGGAATTCCCGGTGTAGCGGTGAAATGCGTAGAT
ATCGGGAGGAACACCAGCGGCGAAAGCGGCTTACTGGGCTGTGCCTGACGCTGAGGTGCGAGAGC
GTGGGGAGCAAACTGGATTAGAAACCCCAGTAGTCC
>OTU254
GCAGGGAATCTTGCGCAATGGGCGAAAGCCTGACGCAGCGACACCGCGTGGGGGAAGAAGGCTTT
CGGGTTGTAAACCCCTTTTCTCGGGGAAGAGAACGGACGGTACCCGAGGAATAAGGTTCGGCTAA
CTACGTGCCAGCAGCCGCGGTAACACGTAGGAACCGAGCGTTGTCCGGATTTACTGGGCGTAAAG
GGCGAGTAGGCGGCCTGCTAAGTCTCATGTGAAATCTCCCGGCTCAACTGGGAGGGGTCATGGGA

AACTGGCAAGCTTGAGGGCAGTAGAGGAAAGTGGAATTCCCGGAGTAGTGGTGGAATGCGTAGAT
ACCGGGAGGAACACCAGTGGCGAAAGCGACTTTCTGGGCTGCACCTGACGCTGAGGCGCGAAAGC
ATGGGGAGCAAACCGGATTAGATACCCTAGTAGTCC

>OTU334
GCAGGGAATTTTGGGCAATGGGCGAAAGCCTGACCCAGCGACGTCGCGTGGGGGATGAAGGCCTT
AGGGCTGTAAACCCCTTTTCCGGGGGAAGAGGAAGGACGGTACTTCGGGAATAAGCTCCGGCTAA
CTACGTGCCAGCAGCCGCGGTAATACGTAGGGAGCAAGCGTTACCCGGAATCACTGGGCGTAAAG
AGATCGTAGGCGGCTAGGTAAGTCTCCTGTGAAAGCTCCCGGCTTAACTGGGAGAGGTCAGGGGA
TACTGCTTGGCTTGAGGGCAGTAGAGGAAGGCGGAATTCCCGGTGTAGCGGTGAAATGCGTAGAT
ATCGGGAGGAACACCAGTGGCGAAAGCGGCCTTCTGGGCTGTGCCTGACGCTGAGGATCGAGAGC
GTGGGGAGCAAACAGGATTAGATACCCTAGTAGTCC
>OTU457
GCAGGGAATCTTGCGCAATGGGCGAAAGCCTGACGCAGCGACGCCGCGTGGGGGAAGAAGGCCCT
CGGGTCGTAAACCCCTTTTCTGGGGGAAGAGTAAGGACGGTACCCCAGGAATAAGCCTCGGCTAA
CTACGTGCCAGCAGCCGCGGTAATACGTAGGAGGCGAGCGTTGTCCGGATTTACTGGGCGTAAAG
GGCGTGTAGGCGGCTATGGAAGTCTTTGGTGAAATCTCCCGGCTCAACTGGGAGGGGTCCAGGGA
TACTCCATGGCTAGAGGGCAGTAGAGGAGAGTGGAATTCCCGGTGTAGTGGTGAAATGCGTAGAT
ATCGGGAGGAACACCAGTGGCGAAAGCGGCTCTCTGGGCTGTACCTGACGCTGAGGCGCGAAAGC
GTGGGGAGCAAACCGGATTAGATACCCTAGTAGTCC
>OTU482
GCAGGGAATTTTGGGCAATGGGCGAAAGCCTGACCCAGCGACGTCGCGTGGGGGACGAAGGCCTT
AGGGCTGTAAACCCCTTTTCCAAGGGATGAGAAAGGACAGTACCTTGGGAATAAGCTCCGGCTAA
CTACGTGCCAGCAGCCGCGGTAATACGTAGGGAGCAAGCGTTACCCGGAATCACTGGGCGTAAAG
AGCGCGTAGGCGGCTGGATAAGTCTTTTGTGAAAGCTCCCGGCTAAACTGGGAGAGGTCAAGAGA
TACTATTCGGCTAGAGGGCAGTAGAGGAAGGCGGAATTCCCGGTGTAGCGGTGAAATGCGTAGAT
ATCGGGAGGAACACCAGTGGCGAAAGCGGCCTTCTGGGCTGCGCCTGACGCTGAGGCGCGAGAGC
GTGGGGAGCAAACTGGATTAGATACCCTAGTAGTCC
>OTU499
GCAGGGAATCTTGCGCAATGGGCGAAAGCCTGACGCAGCGACACCGCGTGGGGGATGAAGGTCTT
AGGATTGTAAACCCCTTTTGTGAGGGAAGAGCAAGGACGGTACCTCACGAATAAGGATCGGCTAA
CTACGTGCCAGCAGCCGCGGTAATACGTAGGATTCGAGCGTTGTCCGGATTTATTGGGCGTAAAG
GGTGTGTAGGCGGCATGGCAAGTCTCATGTGAAATCTCCCGGCTCAACTGGGAGGGGTCATGGGA
AACTGCCAAGCTGGAGGGCAGCAGAGGAAAGCGGAATTCCGGGAGTAGTGGTGAAATGCGTAGAT
ACCCGGAGGAACACCCGAGGCGAAGGCGGCTTTCTGGGCTGTACCTGACGCTGAGACACGAAAGC
GTGGGGAGCAAACCGGATTAGAAACCCTAGTAGTCC
>OTU550
GCAGGGAATATTGCGCAATGGGCGAAAGCCTGACGCAGCGACACCGCGTGGGGGAAGAAGGCTTT
CGGGTTGTAAACCCCTTTTCTCGGGGAAGAGGAAGGACTGTACCCGAGGAATAAGGTTCGGCTAA
CTACGTGCCAGCAGCCGCGGTAATACGTAGGAACCGAGCGTTGTCCGGATTTACTGGGCGTAAAG
GGCGAGTAGGCGGCCTATTAAGTCTCATGTGAAATCTCCCGGCTTAACTGGGAGGGGTCATGGGA
AACTGGCAGGCTCGAGGGCAGCAGAGGAAAGCGGAATTCCCGGAGTAGTGGTGAAATGCGTAGAT
ACCGGGAGGAACACCAGTGGCGAAGGCGGCTTTCTGGGCTGTACCTGACGCTGAGGCGCGAAAGC
GTGGGGAGCAAACCGGATTAGATACCCCAGTAGTCC

>OTU567
GCAGGGAATCTTGCACAATGGGCGAAAGCCTGATGCAGCGACGCCGCGTGAGGGATGAAGGCTCT
AGGGTTGTAAACCTCTTTTCTCAGGGAAGAGTAAGGACGGTACCTGAGGAATAAGCCACGGCTAA
CTACGTGCCAGCAGCCGCGGTAATACGTAGGTGGCGAGCGTTGTCCGGAATTACTGGGCGTAAAG
GGTCCGTAGGCGGCCTGGAAAGTCCTTTGTGAAATCTCCCGGCTTAACTGGGAGAGGTCGAGGGA
TACTTCCAGGCTTGAGGACGTCAGAGGGAGACGGAATTCCCGGTGTAGTGGTGGTATGCGTAGAT
ATCGGGAGGAACACCAGTGGCGAAGGCGGTCTCCTGGGGCGCTCCTGACGCTGAGGGACGAAAGC
GTGGGTAGCAAACCGGATTAGATACCCTAGTAGTCC
>OTU608
GCAGGGAATCTTGCACAATGGGCGAAAGCCTGATGCAGCGACGCCGCGTGAGGGATGAAGGCTCT
AGGGTCGTAAACCTCTTTTCTCAGGGAAGAGCAAGGACGGTACCTGAGGAATAAGCCACGGCTAA
CTACGTGCCAGCAGCCGCGGTAATACGTAGGTGGCAAACGTTGTCCGGAATTACTGGGCGTAAAG
GGCCCGTAGGCGGACCAACAAGTCGTTTGTGAAATCTCCCGGCTCAACCGGGAGTGGTCGAGCGA
AACTGTTGGACTTGAGGATGTCAGAGGAAGACGGAATTCCCGGTGTAGTGGTGAAATGCGTAGAT
ATCGGGAGGAACACCAGTGGCGAAAGCGGTCTTCTGGGGCATTTCTGACGCTGAGGGGCGAAAGC
GTGGGGAGCAAACCGGATTAGAAACCCCAGTAGTCC
>OTU620
GCAGGGAATTTTGCGCAATGGGCGAAAGCCTGACGCAGCGACGCCGCGTGGGGGAAGAAGGCCTT
AGGGTTGTAAACCCCTTTTCTGGGGGAAGAGAGAGGACGGTACCCCAGGAATAAGCCCCGGCTAA
CTACGTGCCAGCAGCCGCGGTAATACGTAGGGGGCGAGCGTTGTCCGGAATTACTGGGCGTAAAG
GGCGCGTAGGCGGCCCAGGAAGTCTCCGGTGAAATCTCCCGGCTCAACTGGGAGGGGTCCGGGGA
AACTCCTGGGCTTGAGGGCAGCAGAGGAGGGTGGAATTCCCGGTGTAGTGGTGAAATGCGTAGAT
ATCGGGAGGAACACCAGTGGCGAAGGCGGCCCTCTGGGCTGTACCTGACGCTGAGGCGCGAAAGC
GTGGGGAGCGAACCGGATTAGAAACCCTAGTAGTCC
>OTU621
GCAGGGAATTTTGGGCAATGGGCGAAAGCCTGACCCAGCGACGTCGCGTGGGGGATGAAGGTCTT
TGGGCTGTAAACCCCTTTTCCAAGGGAAGAGGAAGGACGGTACCTTGGGAATAAGCTCCGGCTAA
CTACGTGCCAGCAGCCGCGGTAATACGTAGGGAGCGAGCGTTACCCGGAATTACTGGGCGTAAAG
AGCGCGTAGGCGGCTGGGTAAGTCTCTTGTGAAAGCTCCCGGCTTAACTGGGAGAGGTCAAGGGA
TACTACTCGGCTTGAGGGCAGTAGAGGAAGGCGGAATTCCCGGTGTAGCGGTGAAATGCGTAGAT
ATCGGGAGGAACACCAGTGGCGAAAGCGGCCTTCTGGACTGTGCCTGACGCTGAGGCGCGAGAGC
GTGGGGAGCAAACTGGATTAGATACCCTAGTAGTCC
>OTU1080
GCAGGGAATTTTGGGCAATGGGCGAAAGCCTGACCCAGCGACGTCGCGTGAGGGATGAAGGCCTT
AGGGCTGTAAACCTCTTTTCCAAGGGAAGAGGAAGGACGGTACTTTGGGAATAAGCTCCGGCTAA
CTACGTGCCAGCAGCCGCGGTAATACGTAGGGAGCTAGCGTTACCCGGAATTACTGGGCGTAAAG
GGCGCGTAGGCGGCTTGGTAAGTCTCTTGTGAAAGCTCCCGGCTTAACTGGGAGAGGTCAAGGGA
TACTACCAGGCTCGAGGGCAGTAGAGGTAAGCGGAATTCCCGGTGTAGCGGTGAAATGCGTAGAT
ATCGGGAGGAACACCAGTGGCGAAAGCGGCTTACTGGGCTGTGCCTGACGCTGAGGCGCGAGAGC
GTGGGGAGCAAACAGGATTAGATACCCTAGTAGTCC
>OTU1196
GCAGGGGATCTTGCGCAATGGGCGAAAGCCTGACGCAGCGACGCCGCGTGGGGGAAGAAGGCCTT
CGGGTTGTAAACCCCTTTTCTTGGGGATGAGGAAGGACGGTACCCGAGGAATAAGCCCCGGCTAA
CTACGTGCCAGCAGCCGCGGTAAGACGTAGGGGGCGAGCGTTGTCCGGATTTACTGGGCGTAAAG

GGCGCGTAGGCGGTGTAGTATGTCCCGGGTGAAAGCCCCTGGCTTAACTGGGGGAGAGCCTGGGA
AACGGCTGCGCTAGAGGACGGCAGAGGCGAGTGGAATTCCCGGTGTAGTGGTGATATGCGTAGAG
ATCGGGAGGAACACCCGTGGCGAAGGCGGCTCGCTGGGCCGTACCTGACGCTAAGGCGCGAAAGC
GTGGGGAGCGAACCGGATTAGAAACCCGTGTAGTCC

>OTU1214
GCAGGGAATCTTGCGCAATGGGCGAAAGCCTGACGCAGCGACACCGCGTGGGGGAAGAAGGCCTT
CGGGTCGTAAACCCCTTTTCTCGGGGAAGAGCAAGGACGGTACCCGAGGAATAAGGTTCGGCTAA
CTACGTGCCAGCAGCCGCGGTAATACGTAGGAACCGAGCGTTGTCCGGAATTATTGGGCGTAAAG
GGCGCGTAGGCGGCTCGTTAAGTCTCATGTGAAATCTCCCGGCTCAACCGGGAGGGGTCATGGGA
TACTGGCGGGCTTGAGGGCAGTAGAGGAAAGCGGAATTCCCGGAGTAGTGGTGATATGCGTAGAT
ACCGGGAGGAACACCAGAGGCGAAGGCGGCTTTCTGGGCTGCTCCTGACGCTGAGGCGCGAAAGC
ATGGGTAGCAAACCGGATTAGAAACCCCTGTAGTCC
>OTU2325
GCAGGGGATCTTGCGCAATGGGCGAAAGCCTGACGCAGCGACGCCGCGTGGGGGAAGAAGGCCTT
CGGGTTGTAAACTCCTTTTGTGGAGGAAGAGAAAGGACGGTACTCCACGAATAAGCCCCGGCTAA
CTACGTGCCAGCAGCCGCGGTAATACGTAGGGGGCGAGCGTTGTCCGGATTTACTGGGCGTAAAG
AGCGCGTAGGCGGTTCAGTGCGTCTCCGGTGAAAGCTCCCGGCTCAACCGGGAGAGGTTCGGAGA
TACGGCTGAGCTAGAGGGCAGTAGAGGAGAGTGGAATTCCCGGTGGAGTGGTGAAATGCGTAGAT
ATCGGGAGGAACACCAGTAGCGAAAGCGGCTCTCTGGGCTGTACCTGACGCTGAGGCGCGAGAGC
GTGGGGAGCAAACCGGATTAGATACCCCTGTAGTCC
>OTU2758
GCAGGGAATCTTGCGCAATGGGCGAAAGCCTGACGCAGCGACACCGCGTGGGGGAAGAAGGCCCT
AGGGTTGTAAACCCCTTTTCTCGGGGAAGAGAACGGACGGTACCCGAGGAATAAGGATCGGCTAA
CTACGTGCCAGCAGCCGCGGTAATACGTAGGATCCGAGCGTTGTCCGGATTTACTGGGCGTAAAG
GGCGCGTAGGCGGCATGTTAAGTCTCATGTGAAATCTCCCGGCTCAACTGGGAGGGGTCATGGGA
AACTGGCAAGCTTGAGGGCAGTAGAGGAAAGCGGAATTCCCGGAGTAGTGGTGAAATGCGTAGAT
ACCGGGAGGAACACCAGAGGCGAAAGCGGCTTTCTGGGCTGTCCCTGACGCTGAGGCGCGAAAGC
ATGGGGAGCAAACCGGATTAGAAACCCTAGTAGTCC
>OTU2764
GCAGGGAATCTTGCGCAATGGGCGAAAGCCTGACGCAGCGACACCGCGTGGGGGAAGAAGGCTCT
AGGGTTGTAAACCCCTTTTCTCGGGGAAGAGTAAGGACGGTACCCGAGGAATAAGGTTCGGCTAA
CTACGTGCCAGCAGCCGCGGTAATACGTAGGAACCGAGCGTTGTCCGGATTTACTGGGCGTAAAG
GGCGCGTAGGCGGCCCATCAAGTCTCATGTGAAATCTCCCGGCTCAACTGGGAGGGGTCATGGGA
AACTGGTAGGCTCGAGGGCAGTAGAGGAAAGCGGAATTCCCGGAGTAGTGGTGATATGCGTAGAT
ACCGGGAGGAACACCAGAGGCGAAGGCGGCTTTCTGGACTGTACCTGACGCTGAGGCGCGAGAGC
ATGGGGAGCAAACCGGATTAGATACCCTAGTAGTCC
>OTU3012
GCAGGGAATCTTGCACAATGGGCGAAAGCCTGATGCAGCGACGCCGCGTGGGGGATGAAGGCTCT
AGGGTCGTAAACCCCTTTTCTCAGGGAAGAGCAAGGACGGTACCTGAGGAATAAGCCACGGCTAA
CTACGTGCCAGCAGCCGCGGTAATACGTAGGTGGCAAGCGTTGTCCGGAATTACTGGGCGTAAAG
GGTCCGTAGGCGGTTCGGTAAGTCTCCTGTGAAATCTCCAGGCTTAACTTGGAGCGGTCGGGGGA
TACTGCCGGACTTGAGACTGTCAGAGGCAAGCGGAATTCCCGGTGTAGTGGTGAAATGCGTAGAT
ATCGGGAGGAACACCAGTGGCGAAGGCGGCTTGCTGGGGCAGTTCTGACGCTGAGGGACGAAAGC

GTGGGTAGCAAACCGGATTAGATACCCTAGTAGTCC
>OTU3081
GCAGGGAATTTTGCGCAATGGGCTAACGCCTGACGCAGCGACGCCGCGTGGGGGAAGAAGGTCTT
AGGGCTGTAAACCCCTTTTCTGGGGGAAGAGAGAGGACGGTACCCCAGGAATAAGCCCCGGCTAA
CTACGTGCCAGCAGCCGCGGTAATACGTAGGGGGCGAGCGTTGTCCGGAGTTACTGGGCGTAAAG
GGCGCGTAGGCGGTCCAGGAAGTCTCTGGTGAAATCTCCCGGCTTAACTGGGAGGGGTCCGGGGA
AACTCTTGGGCTTGAGGACAGCAGAGGAGGGTGGAATTCCCGGTGTAGTGGTGAAATGCGTAGAT
ATCGGGAGGAACACCAGTGGCGAAGGCGGCCCTCTGGGCTGTGCCTGACGCTGAGGCGCGAAAGC
GTGGGGAGCGAACCGGATTAGATACCCCTGTAGTCC
>OTU3250
GCAGGGAATCTTGCGCAATGGACGAAAGTCTGACGCAGCGACACCGCGTGGAGGATGAAGGCCTT
AGGGTTGTAAACTCCTTTTATAGGGGAAGAGAACGGACGGTACCTTATGAATAAGGATCGGCTAA
CTACGTGCCAGCAGCCGCGGTAATACGTAGGATCCGAGCGTTGTCCGGATTTACTGGGCGTAAAG
GGTGCGTAGGCGGTTTGTCAAGTCTTATGTGAAATCTCCCGGCTCAACTGGGAGGGGTCATAGGA
AACTGGCAGACTTGAGGGCAGTAGAGGAGAGCGGAATTCCTGGAGTAGCGGTGATATGCGTAGAT
ACCAGGAGGAACACCAGAGGCGAAAGCGGCTCTCTGGGCTGTACCTGACGCTGAGGCACGAAAGC
GTGGGGAGCAAACCGGATTAGAAACCCTAGTAGTCC
>OTU3729
GCAGGGAATTTTGCGCAATGGGCGAAAGCCTGACGCAGCGACGCCGCGTGAGGGAAGAAGGCCTT
CGGGTTGTAAACCTCTTTTCTGGGGGAAGAGCAAGGACGGTACCCCAGGAATAAGCCCCGGCTAA
CTACGTGCCAGCAGCCGCGGTAATACGTAGGGGGCGAGCGTTGTCCGGAATTACTGGGCGTAAAG
GGCGCGTAGGCGGCCCAATAAGTCTCCGGTGAAATCTCCCGGCTTAACTGGGAGCGGTCCGGGGA
AACTATTGGGCTTGAGGACAGCAGAGGAGAGTGGAATTCCCGGTGTAGTGGTGAAATGCGTAGAT
ATCGGGAGGAACACCAGTGGCGAAGGCGGCTCTCTGGGCTGTACCTGACGCTGAGGCGCGAAAGC
GTGGGGAGCAAACCGGATTAGAAACCCTAGTAGTCC
>OTU4047
GCAGGGAATTTTGGGCAATGGACGAAAGTCTGACCCAGCGACGTCGCGTGGGGGATGAAGGCTCT
AGGGCTGTAAACCCCTTTTCCGAGGGAAGAGGAAGGACGGTACCTCGGGAATAAGCTCCGGCTAA
CTACGTGCCAGCAGCCGCGGTAATACGTAGGGAGCGAGCGTTACCCGGAATTACTGGGCGTAAAG
AGCACGTAGGCGGTTGGATAAGTCTTCTGTGAAAGCTCCCGGCTCAACTGGGAGAGGTCAGGTGA
TACTATTCGACTTGAGGGCTGTAGAGGTAAGCGGAATTCCCGGTGTAGCGGTGAAATGCGTAGAT
ATCGGGAGGAACACCAGCGGCGAAAGCGGCTTACTGGGCAGTGCCTGACGCTGAGGTGCGAGAGC
GTGGGGAGCAAACTGGATTAGAAACCCTAGTAGTCC
>OTU4134
GCAGGGAATCTTGCGCAATGGGCGAAAGCCTGACGCAGCGACACCGCGTGGGGGAAGAAGGCCTT
CGGGTTGTAAACCCCTTTTCTGGGGGAAGAGGAAGGACGGTACCCCAGGAAGAAGCCTCGGCTAA
CTACGTGCCAGCAGCCGCGGTAAGACGTAGGAGGCGAGCGTTGTCCGGATTTACTGGGCGTAAAG
AGCGCGTAGGCGGTGAAGGAAGTCCACGGTGAAAGTTCCCGGCTCAACTGGGAAAGGTCCGAGGA
TACTCCTTCACTTGAGGGCAGCAGAGGCGGGTGGAATTCCCGGCGTAGCGGTGAAATGCGTAGAA
TTCGGGAGGAACACCAGAGGCGAAGGCGGCCCGCTGGGCTGCACCTGACGCTGAGGCGCGATAGC
GTGGGGAGCAAACCGGATTAGAAACCCCAGTAGTCC
>OTU4183
GCAGGGAATCTTGCGCAATGGGCGAAAGCCTGACGCAGCGACGTCGCGTGGGGGAAGAAGGTCTT
CGGGTCGTAAACCCCTTTTCTGGGGGAAGAGTAAGGACGGTACCCCAGGAATAAGCCTCGGCTAA

CTACGTGCCAGCAGCCGCGGTAATACGTAGGAGGCGAGCGTTGTCCGGATTTACTGGGCGTAAAG
GGCGTGTAGGCGGTTCGGGAAGTCCCTGGTGAAATCTCCCAGCTCAACTGGGAGCGGTCTGGGGA
TACTCTCGGACTTGAGGGCAGTAGAGGAGAGTGGAATTCCCGGTGGAGTGGTGAAATGCGTAGAT
ATCGGGAGGAACACCCGTGGCGAAAGCGGCTCTCTGGGCTGTCCCTGACGCTGAGGCGCGAAAGC
GTGGGGAGCAAACCGGATTAGATACCCTAGTAGTCC
>OTU4705
GCAGGGAATCTTGCGCAATGGGCGAAAGCCTGACGCAGCGACACCGCGTGGGGGAAGAAGGCCTT
CGGGTTGTAAACCCCTTTTCTCGGGGAAGAGGAAGGACGGTACCCGAGGAATAAGGTTCGGCTAA
CTACGTGCCAGCAGCCGCGGTAATACGTAGGAACCGAGCGTTGTCCGGATTTATTGGGCGTAAAG
GGCGCGTAGGCGGCCCGTTAAGTCTCATGTGAAATCTCCCGGCTCAACTGGGAGCGGTCATGGGA
AACTGACAGGCTCGAGGGCAGTAGAGGAAAGTGGAATTCCCGGAGTAGTGGTGAAATGCGTAGAT
ACCGGGAGGAACACCAGAGGCGAAGGCGACTTTCTGGGCTGTACCTGACGCTGAGGCGCGAGAGC
GTGGGGAGCAAACCGGATTAGAAACCCGAGTAGTCC
>OTU4304
GCAGGGAATCTTGCGCAATGGGCGAAAGCCTGACGCAGCGACACCGCGTGAAGGAAGAATGCCTT
CGGGTTGTAAACTTCTTTTATCAGGGAAGAGATAGGACTGTACCTGATGAATAAGCCCCGGCTAA
CTACGTGCCAGCAGCCGCGGTAATACGTAGGGGGCGAGCGTTGTCCGGAATTATTGGGCGTAAAG
GGCGAGTAGGCGGCACGTCAAGTCCCATGTGAAATCTCCCGGCTTAACCGGGAGCGGTCATGGGA
AACTGGCGAGCTTGAGGACAGCAGAGGAGAGCGGAATTCCCGGAGTAGTGGTGAAATGCGTAGAT
ACCGGGAGGAACACCAGTGGCGAAAGCGGCTCTCTGGGCTGCCCCTGACGCTGAGGCGCGAAAGC
ATGGGGAGCAAACCGGATTAGAAACCCCAGTAGTCC
>OTU4337
GCAGGGAATCTTGCACAATGGGCGAAAGCCTGATGCAGCGACGCCGCGTGAGGGATGAAGGCTTT
CGAGTTGTAAACCTCTTTTCTCAGGGAAGAGTAAGGACGGTACCTGAGGAATAAGCCACGGCTAA
CTACGTGCCAGCAGCCGCGGTAATACGTAGGTGGCGAGCGTTGTCCGGAATTACTGGGCGTAAAG
GGCCCGTAGGCGGTCTGGAAAGTCTTCTGTGAAATCTCCCGGCTCAACTGGGAGAGGTCGGGAGA
AACTTCCAGACTTGAGGGCGTCAGAGGGAGACGGAATTCCCGGTGTAGTGGTGAAATGCGTAGAT
ATCGGGAGGAACACCAGTGGCGAAGGCGGTCTCCTGGGGCGCCCCTGACGCTGAGGGGCGAAAGC
GTGGGGAGCAAACCGGATTAGAAACCCCAGTAGTCC
>OTU5274
GCAGGGAATTTTGGGCAATGGACGAAAGTCTGACCCAGCGACGTCGCGTGGGGGATGAAGGCCTT
AGGGCTGTAAACCCCTTTTCCGGGGGAAGAGGAAGGACGGTACTTCGGGAATAAGCTCCGGCTAA
CTACGTGCCAGCAGCCGCGGTAATACGTAGGGAGCAAGCGTTACCCGGAATCACTGGGCGTAAAG
AGATCGTAGGCGGCTAGGTAAGTCTCCTGTGAAAGCTCCCGGCTTAACTGGGAGAGGTCAGGGGA
TACTGCTTGGCTTGAGGGCAGTAGAGGAAGGCGGAATTCCCGGTGTAGCGGTGAAATGCGTAGAT
ATCGGGAGGAACACCAGTGGCGAAAGCGGCCTTCTGGGCTGTGCCTGACGCTGAGGATCGAGAGC
GTGGGGAGCAAACAGGATTAGAAACCCTAGTAGTCC
>OTU5700
GCAGGGAATTTTGGGCAATGGGCGAAAGCCTGACCCAGCGACGTCGCGTGGGGGATGAAGGCCTT
AGGGCTGTAAACCCCTTTTCCGAGGGAAGAGGAAGGACGGTACCTCGGGAATAAGCTCCGGCTAA
CTACGTGCCAGCAGCCGCGGTAATACGTAGGGAGCTAGCGTTACCCGGAATTACTGGGCGTAAAG
GGCGCGTAGGCGGCTGGATAAGTCTCTTGTGAAAGCTCCCGGCTTAACTGGGAGAGGTCAAGGGA
TACTATTCGGCTTGAGGGCAGTAGAGGAAGGCGGAATTCCCGGTGTAGCGGTGAAATGCGTAGAT
ATCGGGAGGAACACCAGTGGCGAAAGCGGCCTTCTGGGCTGTGCCTGACGCTGAGGCGCGAGAGC

GTGGGGAGCAAACTGGATTAGAAACCCCAGTAGTCC
>OTU6587
GCAGGGAATCTTGCGCAATGGGCGAAAGCCTGACGCAGCGACGCCGCGTGGGGGAAGAAGGCTTT
CGGGTTGTAAACCCCTTTTCTGGGGGAAGAGTAAGGACGGTACCCCAGGAATAAGCCTCGGCTAA
CTACGTGCCAGCAGCCGCGGTAACACGTAGGAGGCAAGCGTTGTCCGGATTTACTGGGCGTAAAG
GGCGTGTAGGCGGTTTGGGAAGTCTTTGGTGAAATCTCCCGGCTCAACTGGGAGGGGTCCAGAGA
TACTCCCAGACTAGAGGGCAGTAGAGGAGAGTGGAATTCCCGGTGTAGTGGTGAAATGCGTAGAT
ATCGGGAGGAACACCAGTGGCGAAAGCGGCTCTCTGGGCTGTACCTGACGCTGAGGCGCGAAAGC
GTGGGGAGCGAACCGGATTAGATACCCCAGTAGTCC
>OTU7241
GCAGGGAATCTTGCGCAATGGGCGAAAGCCTGACGCAGCGACGCCGCGTGGGGGAAGAAGGTCTT
CGGGCTGTAAACCTCTTTTCTGGGGGAAGAGTAAGGACGGTACCCCAGGAATAAGCCTCGGCTAA
CTACGTGCCAGCAGCCGCGGTAATACGTAGGAGGCGAGCGTTGTCCGGATTTACTGGGCGTAAAG
GGCGTGTAGGCGGTCTGGAAAGTCTTTGGTGAAATCTCCCGGCTCAACCGGGAGGGGTCCAGGGA
TACTTCCAGACTAGAGGGCAGTAGAGGAGAGTGGAATTCCCGGTGTAGTGGTGAAATGCGTAGAT
ATCGGGAGGAACACCAGTGGCGAAAGCGGCTCTCTGGGCTGTACCTGACGCTGAGGCGCGAAAGC
GTGGGGAGCAAACCGGATTAGAAACCCCAGTAGTCC
>OTU2525
GCAGGGAATCTTCCACAATGGGCGAAAGCCTGATGGAGCGACGCCGCGTGAGGGATGAAGGCTCT
AGGGTCGTAAACCTCTTTTCTCAGGGAAGAGCAAGGACGGTACCTGAGGAATAAGTGACGGCTAA
CTACGTGCCAGCAGCCGCGGTAATACGTAGGTCACAAACGTTGTCCGGATTTATTGGGCGTAAAG
GGTCCGTAGGCGGTTCGGTAAGTCTCCTGTGAAATCTTCAGGCTCAACTTGAAGAGGTCGGGGGA
TACTGCCGGACTTGAGACTGTTAGAGGCAAGTGGAATTCCCGGTGTAGTGGTGAAATGCGTAGAT
ATCGGGAGGAACACCAGTGGCGAAAGCGGCTTGCTGGGACAGTTCTGACGCTGAGGGACGAAAGC
GTGGGTAGCAAACCGGATTAGAAACCCTAGTAGTCC
>OTU2622
GCAGGGAATCTTCCACAATGGGCGAAAGCCTGATGGAGCGACGCCGCGTGAGGGATGAAGGCTTT
AGGGTCGTAAACCTCTTTTCTCAGGGAAGAGCAAGGACGGTACCTGAGGAATAAGTGACGGCTAA
CTACGTGCCAGCAGCCGCGGTAATACGTAGGTCACAAACGTTGTCCGGAATTATTGGGCGTAAAG
GGTCCGTAGGCGGTTCGGTAAGTCTCCTGTGAAATCTTCAGGCTCAACTTGAAGAGGTCGGGGGA
TACTGCTGGACTAGAGACTGACAGAGGCAAGTGGAATTCCCGGTGTAGTGGTGAAATGCGTAGAT
ATCGGGAGGAACACCAGTGGCGAAAGCGGCTTGCTGGGTCAGTTCTGACGCTGAGGGACGAAAGC
GTGGGTAGCAAACCGGATTAGAAACCCTAGTAGTCC
>OTU2505
GCAGGGAATCTTGCGCAATGGGCGAAAGCCTGACGCAGCGACACCGCGTGGGGGATGAAGGCCTT
AGGGTTGTAAACCCCTTTTCTCGGGGAAGAGGACGGACGGTACCCGAGGAATAAGGATCGGCTAA
CTACGTGCCAGCAGCCGCGGTAATACGTAGGATCCGAGCGTTGTCCGGATTTACTGGGCGTAAAG
GGTGCGTAGGCGGCGTGGTAAGTCTCATGTGAAATCTCCCGGCTCAACTGGGAGGGGTCATGGGA
TACTGCCATGCTCGAGGGCAGTAGAGGAGAGCGGAATTCCGGGAGTAGCGGTGATATGCGTAGAT
ACCCGGAGGAACACCCGTGGCGAAAGCGGCTCTCTGGACTGTACCTGACGCTAAGGCACGAAAGC
GTGGGGAGCAAACCGGATTAGAAACCCTAGTAGTCC
>OTU2492
GCAGGGAATTTTGGGCAATGGACGAAAGTCTGACCCAGCGACGTCGCGTGGGGGATGAAGGCTCT
AGGGCTGTAAACCCCTTTTCCGAGGGAAGAGGAAGGACGGTACCTCGGGAATAAGCTCCGGCTAA

CTACGTGCCAGCAGCCGCGGTAATACGTAGGGAGCGAGCGTTACCCGGAATTACTGGGCGTAAAG
AGCACGTAGGCGGTTGGATAAGTCTTCTGTGAAAGCTCCCGGCTTAACTGGGAGAGGTCAGGTGA
TACTATTCGACTTGAGGGCTGTAGAGGTAAGCGGAATTCCCGGTGTAGCGGTGAAATGCGTAGAT
ATCGGGAGGAACACCAGCGGCGAAAGCGGCTTACTGGGCAGTGCCTGACGCTGAGGTGCGAGAGC
GTGGGGAGCAAACAGGATTAGAAACCCGAGTAGTCC
>OTU2478
GCAGGGAATCTTGCGCAATGGGCGAAAGCCTGACGCAGCGACACCGCGTGGGGGAAGAAGGCCTT
CGGGTCGTAAACCCCTTTTCCCAGGGAGGAGAATGGACAGTACCTGGGGAATAAGGATCGGCTAA
CTACGTGCCAGCAGCCGCGGTAATACGTAGGATCCGAGCGTTGTCCGAATTTACTGGGCGTAAAG
GGCGCGTAGGCGGCCCGCTAAGTCTCATGTGAAATCTCCCGGCTCAACTGGGAGGGGTCATGGGA
AACTGGCAGGCTTGAGGGCAGCAGAGGGAAGCGGAATTCCGGGAGTAGTGGTGAAATGCGTAGAT
ACCCGGAGGAACACCAGTGGCGAAAGCGGCTTCCTGGACTGTACCTGACGCTGAGGCGCGAAAGC
GTGGGGAGCAAACCGGATTAGAAACCCCAGTAGTCC
>OTU2493
GCAGGGAATCTTGCGCAATGGGCGAAAGCCTGACGCAGCGACACCGCGTGGAGGATGAAGGCCTT
AGGGTTGTAAACTCCTTTTATTGGGGAAGAGAACGGACGGTACCTAATGAATAAGGATCGGCTAA
CTACGTGCCAGCAGCCGCGGTAATACGTAGGATCCGAGCGTTGTCCGGATTTACTGGGCGTAAAG
GGTGCGTAGGCGGTTTGTTAAGTCTCATGTGAAATCTCCCGGCTCAACTGGGAGGGGTCATGGGA
TACTGGCAGACTTGAGGGCAGTAGAGGAGAGCGGAATTCCTGGAGTAGCGGTGATATGCGTAGAT
ACCAGGAGGAACACCAGAGGCGAAAGCGGCTCTCTGGGCTGTACCTGACGCTGAGGCACGAAAGC
GTGGGGAGCAAACCGGATTAGAAACCCGAGTAGTCC
>OTU2512
GCAGGGAATCTTCCACAATGGGCGAAAGCCTGATGGAGCGACACCGCGTGGGGGATGAAGGCCTT
AGGGTCGTAAACCCCTTTTCTCAGGGAAGAGCAAGGACGGTACCTGAGGAATAAGCATCGGCTAA
CTACGTGCCAGCAGCCGCGGTAATACGTGGGATGCAAGCGTTGTCCGGAATTACTGGGCGTAAAG
GGTCCGTAGGCGGTTTGGTAAGTCTCCTGTGAAAACTCCGGGCTTAACCCGGAGAGGTCGGGGGA
TACTGCTGAACTTGAGACTGTCAGAGGCAAGCGGAATTCCCGGTGTAGTGGTGAAATGCGTAGAT
ATCGGGAGGAACTCCAGTGGCGAAAGCGGCTTGCTGGGACAGTTCTGACGCTGAGGGACGAAAGC
GTGGGTAGCAAACCGGATTAGAAACCCTAGTAGTCC
>OTU2883
GCAGGGAATCTTGCGCAATGGGCGAAAGCCTGACGCAGCGACACCGCGTGAAGGAAGAATGCCTT
CGGGTTGTAAACTTCTTTTATCAGGGAAGAGATAGGACTGTACCTGGTGAATAAGCCCCGGCTAA
CTACGTGCCAGCAGCCGCGGTAATACGTAGGGGGCGAGCGTTGTCCGGATTTATTGGGCGTAAAG
GGCGAGTAGGCGGCACGCCAAGTCCCGTGTGAAATCTCCCGGCTTAACTGGGAGAGGTCATGGGA
AACTGGCGAGCTTGAGGACAGCAGAGGAGAGCGGAATTCCCGGAGTAGTGGTGAAATGCGTAGAT
ACCGGGAGGAACACCAGAGGCGAAAGCGGCTCTCTGGGCTGCACCTGACGCTGAGGCGCGAAAGC
ATGGGGAGCAAACCGGATTAGAAACCCCGGTAGTCC
>OTU3153
GCAGGGAATTTTGCGCAATGGGCGAAAGCCTGACGCAGCGACGCCGCGTGGGGGAAGAAGGCCTT
CGGGTTGTAAACTCCTTTTCTGGGGGAAGAGATAGGACGGTACCCCAGGAATAAGCCCCGGCTAA
CTACGTGCCAGCAGCCGCGGTAATACGTAGGGGGCGAGCGTTGTCCGGAATTACTGGGCGTAAAG
AGCGCGTAGGCGGCCCAGGAAGTCCTTGGTGAAATCTCCCGGCTCAACTGGGAGGGGTCCGAGGA
AACTCCTGGGCTAGAGGATGGCAGAGGAGGGTGGAATTCCCGGTGTAGTGGTGAAATGCGTAGAT
ATCGGGAGGAACACCAGTGGCGAAGGCGGCCCTCTGGGCTGTACCTGACGCTGAGGCGCGAAAGC

GTGGGGAGCAAACCGGATTAGAAACCCTAGTAGTCC
>OTU3051
GCAGGGAATTTTGCGCAATGGGCGAAAGCCTGACGCAGCGACGCCGCGTGGGGGAAGAAGGCCTT
AGGGTTGTAAACTCCTTTTCTGGGGGAAGAGCAAGGACGGTACCCCAGGAATAAGCCCCGGCTAA
CTACGTGCCAGCAGCCGCGGTAATACGTAGGGGGCGAGCGTTGTCCGGAATTACTGGGCGTAAAG
GGCGCGTAGGCGGCCCAAGAAGTCTCCGGTGAAATCTCCCGGCTTAACTGGGAGGGGTCCGGGGA
AACTCTTGGGCTTGAGGACAGCAGAGGAGGGTGGAATTCCCGGTGTAGTGGTGAAATGCGTAGAT
ATCGGGAGGAACACCAGTGGCGAAGGCGGCCCTCTGGGCTGTACCTGACGCTGAGGCGCGAAAGC
GTGGGTAGCAAACCGGATTAGAAACCCTTGTAGTCC

>OTU2606
GCAGGGAATCTTGCGCAATGGGCGAAAGCCTGACGCAGCGACACCGCGTGGGGGAAGAATGCCCT
AGGGTTGTAAACCCCTTTTCTCGGGGAAGAGAACGGACGGTACCCGAGGAATAAGGATCGGCTAA
CTACGTGCCAGCAGCCGCGGTAACACGTAGGATTCGAGCGTTGTCCGGAATTACTGGGCGTAAAG
GGCGCGTAGGTGGCATGTTAAGTCTCATGTGAAATCTCCCGGCTCAACTGGGAGGGGTCATGGGA
AACTAACAAGCTTGAGGGCAGTAGAGGAAAGCGGAATTCCCGGAGTAGTGGTGAAATGCGTAGAT
ACCGGGAGGAACACCAGAGGCGAAAGCGGCTTTCTGGGCTGTCCCTGACACTGAGGCGCGAAAGC
ATGGGGAGCAAACCGGATTAGAAACCCCAGTAGTCC
>OTU2973
GCAGGGAATCTTGCGCAATGGGCGAAAGCCTGACGCAGCGACACCGCGTGGGGGAAGAAGGCCTT
CGGGTCGTAAACCCCTTTTCTCAGGGAAGAGCAAGGACGGTACTTGAGGAATAAGGTTCGGCTAA
CTACGTGCCAGCAGCCGCGGTAATACGTAGGAACCGAGCGTTGTCCGGAATTATTGGGCGTAAAG
GGCGCGTAGGCGGCCCGTTAAGTCTCATGTGAAATCTCCCGGCTCAACTGGGAGGGGTCATGGGA
AACTGGCAGGCTCGAGGGCAGTAGAGGAAAGTGGAATTCCCGGAGTAGTGGTGATATGCGTAGAT
ACCGGGAGGAACACCAGTGGCGAAGGCGGCTTTCTGGGCTGCTCCTGACGCTGAGGCGCGAAAGC
ATGGGTAGCAAACCGGATTAGAAACCCCTGTAGTCC
>OTU3000
GCAGGGAATCTTGCGCAATGGGCGAAAGCCTGACGCAGCGACGCCGCGTGGAGGAAGAAGGCCTT
CGGGTTGTAAACTCCTTTTCTGGGGGAAGAGAACGGACGGTACCCCAGGAATAAGCCTCGGCTAA
CTACGTGCCAGCAGCCGCGGTAATACGTAGGAGGCAAGCGTTGTCCGGATTTACTGGGCGTAAAG
GGCGTGTAGGCGGCATGGGAAGTCTTTGGTGAAATCTCCCGGCTCAACCGGGAGGGGTCCAGGGA
AACTCCCAAGCTAGAGGGCAGTAGAGGAGAGTGGAATTCCCGGTGTAGTGGTGAAATGCGTAGAT
ATCGGGAGGAACACCAGTGGCGAAAGCGGCTCTCTGGGCTGTACCTGACGCTGAGGCGCGAAAGC
GTGGGTAGCGAACCGGATTAGATACCCTAGTAGTCC
>OTU1674
GCAGGGAATCTTGGGCAATGGGCGAAAGCCTGACCCAGCGACGCCGCGTGGGGGACGAAGGCCTT
CGGGTTGTAAACCCCTTTTCCAGGGGAAGAGGAAGGACGGTACCCTGGGAATAAGCCTCGGCTAA
CTACGTGCCAGCAGCCGCGGTAATACGTAGGAGGCGAGCGTTGTCCGGATTTACTGGGCGTAAAG
GGCGTGTAGGCGGCCGAGCAAGTCTTCTGTGAAATCTCCCGGCTTAACCGGGAAAGGTCAGAGGA
TACTACTCGGCTTGAGGGCAGCAGAGGAGGGTGGAATTCCCGGTGTAGCGGTGGTATGCGTAGAT
ATCGGGAGGAACACCAGTGGCGAAGGCGGCCCTCTGGGCTGTCCCTGACGCTGAGGCGCGAAAGC
GTGGGGAGCGAACCGGATTAGATACCCTAGTAGTCC
>OTU2955
GCAGGGAATCTTGCGCAATGGGCGAAAGCCTGACGCAGCGACACCGCGTGGGGGAAGAAAGCTTT

CGAGTTGTAAACCCCTTTTCCCGGGGAAGAGAACGGACGGTACCCGGGGAATAAGGTTCGGCTAA
CTACGTGCCAGCAGCCGCGGTAATACGTAGGAACCGAGCGTTGTCCGGATTTACTGGGCGTAAAG
TGCGCGTAGGCGGTCTGTTAAGTCTCATGTGAAATCTCCCGGCTCAACTGGGAGGGGTCATGGGA
AACTGGCAGGCTTGAGGGCAGCAGAGGAGAGTGGAATTCCCGGAGTAGTGGTGAAATGCGTAGAT
ACCGGGAGGAACACCAGAGGCGAAGGCGACTCTCTGGGCTGTACCTGACGCTGAGGCGCGAAAGC
GTGGGGAGCAAACCGGATTAGAAACCCGAGTAGTCC
